# Inhibition of the mTOR pathway and reprogramming of protein synthesis by MDM4 reduce ovarian cancer metastatic properties

**DOI:** 10.1038/s41419-021-03828-z

**Published:** 2021-05-29

**Authors:** Rossella Lucà, Maria Rita Assenza, Fabio Maiullari, Luisa Pieroni, Silvia Maiullari, Giulia Federici, Federica Marini, Roberto Rizzi, Andrea Urbani, Silvia Soddu, Fabiola Moretti

**Affiliations:** 1 Institute of Biochemistry and Cell Biology, National Research Council of Italy (CNR), Monterotondo, Italy; 2 Gemelli Molise SpA, Campobasso, Italy; 3 National Institute of Molecular Genetics, Milan, Italy; 4 Proteomic and Metabolomic Unit, IRCCS-S.Lucia Foundation, Rome, Italy; 5 Catholic University of Sacred Heart, Rome, Italy; 6 Unit of Cellular Networks and Molecular Therapeutic Targets, Department of Research and Advanced Technologies, IRCCS Regina Elena National Cancer Institute, Rome, Italy; 7 Institute of Biomedical Technologies, CNR, Segrate, Italy; 8 Fondazione Policlinico Universitario Agostino Gemelli IRCCS, Rome, Italy; 9 IRCCS Regina Elena National Cancer Institute, Rome, Italy

**Keywords:** Metastasis, Mechanisms of disease

## Abstract

Epithelial ovarian cancer (EOC) is a highly heterogeneous disease with a high death rate mainly due to the metastatic spread. The expression of MDM4, a well-known p53-inhibitor, is positively associated with chemotherapy response and overall survival (OS) in EOC. However, the basis of this association remains elusive. We show that in vivo MDM4 reduces intraperitoneal dissemination of EOC cells, independently of p53 and an immune-competent background. By 2D and 3D assays, MDM4 impairs the early steps of the metastatic process. A 3D-bioprinting system, ad hoc developed by co-culturing EOC spheroids and endothelial cells, showed reduced dissemination and intravasation into vessel-like structures of MDM4-expressing cells. Consistent with these data, high *MDM4* levels protect mice from ovarian cancer-related death and, importantly, correlate with increased 15 y OS probability in large data set analysis of 1656 patients. Proteomic analysis of EOC 3D-spheroids revealed decreased protein synthesis and mTOR signaling, upon MDM4 expression. Accordingly, MDM4 does not further inhibit cell migration when its activity towards mTOR is blocked by genetic or pharmacological approaches. Importantly, high levels of MDM4 reduced the efficacy of mTOR inhibitors in constraining cell migration. Overall, these data demonstrate that MDM4 impairs EOC metastatic process by inhibiting mTOR activity and suggest the usefulness of MDM4 assessment for the tailored application of mTOR-targeted therapy.

## Introduction

Epithelial ovarian carcinoma (EOC) is the gynecological tumor with the highest death rate^1,2^. One important factor concurring with this aggressiveness is the rapid and silent dissemination of ovarian cancer cells to the peritoneal cavity, with up to 75% of patients showing transcoelomic metastasis at presentation^2^.

EOC metastasization occurs through exfoliation of malignant cells from the fimbriae to the ovarian surface and by passive movement of single cells or cell aggregates through the peritoneal fluid or ascites to colonize the peritoneal wall^3^. Moreover, hematogenous dissemination has been identified, occurring mainly in highly advanced stage disease^4,5^. All these routes of EOC metastasization are associated with enhanced survival of metastatic cells to various external stimuli, including increased resistance to chemotherapy^6^.

Considering the impact of metastatic disease in ovarian cancer-related deaths, in-depth understanding of the cellular and molecular aspects of EOC metastasis is crucial to overcome this life-threatening disease. Despite the recognition of different histotypes and subgroups, a signature predictive of EOC tumor progression has not yet been identified. This is attributed to the high heterogeneity of the disease with at least 15 oncogenes and 168 epigenetic alterations implicated, making targeted therapy for ovarian cancer highly challenging^2,7,8^.

Recently, in high-grade serous ovarian carcinoma (HGSOC), the most frequent ovarian cancer histotype carrying p53 mutation in 96% of cases, animal and clinical data have indicated the ability of MDM4 to confer cell sensitivity to platinum, the elective treatment for EOC, and the association of its subcellular localization with clinical prognosis^9^. MDM4 is a well-known inhibitor of p53 and based on this function, MDM4 gene amplification or protein overexpression has been found in human tumors with wild-type p53^10^.

However, this protein has demonstrated additional functions also independently of p53. Specifically, it can promote cell apoptosis upon genotoxic stress^11–17^ or ferroptosis^18^. Moreover, two recent reports showed the ability of MDM4 to counteract cell metabolism^19,20^ and to reduce the therapeutic value of mTOR inhibitors on breast tumor spheroid formation, independently of p53^19^. In agreement with these findings, a significant reduction of MDM4 protein has been associated with worse clinicopathological features in breast cancers, independently of p53 background^21^.

In this work, we investigated the activity of MDM4 in EOC. Particularly, since in EOC, the metastatic potential is tightly linked to chemoresistance, we investigated whether MDM4 affects ovarian cancer progression by impairing metastatic properties.

## Materials and methods

### Mice

Animal studies obtained ethical approval by the Ministry of Health (Protocol No. 774/2017-PR) and conducted conforming to the institutional guidelines in compliance with Italian laws (DL N116, GU, suppl 40, 18-2-1992), international laws and policies (European Community Council Directive 86/609, OJa L 358, 12 December 1987; National Institutes of Health Guide for the Care and Use of Laboratory Animals, US National Research Council, 1996).

Animals were housed with food and water ad libitum at 21°C and with a 12 hours light/dark cycle. Eight–10 weeks old C57BL/6 J female mice were obtained from CNR EMMA-INFRAFRONTIER-IMPC, ‘A. Buzzati-Traverso’ International Campus, Monterotondo Scalo. 8 weeks old NSG female mice were obtained from Charles River (Charles River Laboratories Italia). Lentivirus-infected ID8 (5 × 10^6^ cells) or SK-OV-3 (5 × 10^6^) cells overexpressing MDM4 or control (Empty Vector) were resuspended in 200 µl of phosphate buffered saline (PBS) and intraperitoneally (IP) injected, respectively, in C57BL/6 or NSG mice. For ID8 the experiment was conducted using 6 animals for *Empty Vector* and 7 for *MDM4*. The experiment was repeated with *n* = 10 mice for both conditions and metastasis counted blindly by two independent observers. For SK-OV-3 *Empty Vector* or *MDM4* IP injection, six animals per condition were used.

### Multicellular tumor spheroids (MCTSs) three-dimensional invasion assay

ID8 and SK-OV-3 cells were resuspended in DMEM-F12 and 2 × 10^3^ cells/spheroid were suspended in 20% methylcellulose in culture media plus EGF and grown as hanging drops (HD) according to ref. ^22^. Briefly, HD cultures were incubated for 18 h and the resulting cellular aggregates were embedded into a three-dimensional collagen gel. A collagen solution was prepared to consist of 1.5 mg/ml collagen type I (Millipore, rat tail), 2 mg/ml bicarbonate in methylcellulose plus 40% fetal bovine serum, and pH controlled. Five hundred microliters of this solution were added to 24-well plates and spheroids were implanted into the gel using a large pipette tip. After gelation at 37°C in a humidified atmosphere with 5% CO_2_ for 20 min, the gel was overlaid with 500 μl of culture media supplemented with EGF. Tumor cell spheroids were fixed with 4% paraformaldehyde (PFA) and cell invasion was evaluated using the Fiji software (RRID:SCR_002285). The invading area was calculated as follows: total MCTS area at the indicated time point minus MCTS body area at t0.

### Wound-healing assay

Confluent A-2780 (siRNA-CTRL or -MDM4), and *Empty Vector* or *MDM4-*SK-OV-3 or ID8 cells were scratched using a plastic pipette tip. The migration of the cells at the edge of the scratch was analyzed at 0, 24, and 48 hours, using time-lapse imaging (EVOS M5000, Thermo fisher). The images were analyzed by Fiji (RRID:SCR_002285). For the cell velocity analysis, a mixed population of *GFP-Empty Vector* and *mCherry-MDM4*-labeled cells were subjected to wound healing and cell velocity analyzed using the TrackMate Plug-in^23^. For wound-healing assay with rapamycin treatment, *Empty Vector* and *MDM4*-SK-OV-3 confluent cells were scratched using a plastic pipette tip and treated with Rapamycin 100 nM. Time-lapse image acquisition was performed every 45 minutes with a ×4 objective through the IncuCyte S3 Live-Cell System (Sartorius). Cell migration of rapamycin-treated cells was calculated using the Cell Profiler 3.1.9 (CellProfiler Image Analysis Software, RRID:SCR_00735) using the cell migration pipeline.

### Co-immunoprecipitation and western blot

For co-immunoprecipitation (Co-IP), cells were lysed in CHAPS lysis buffer (40 mM Hepes pH 7.4, 120 mM NaCl, 2 mM ethylenediaminetetraacetic acid (EDTA), 0.3% CHAPS) containing a mix of protease inhibitors, 5 mM NaF, 10 mM glycerophosphate, and 1 mM Na_3_VO_4_. Lysates were preincubated with protein G-Agarose (Pierce) and then with anti-MDM4 antibody (Sigma), under gentle rocking at +4°C overnight.

Cells and tumor lesions were lysed in radioimmunoprecipitation assay (RIPA) buffer (50 mM Tris-HCl pH 7.4, 150 mM NaCl, 1%, DOC, 1% NP-40) supplemented with 5 mM β-glycerophosphate, 0.5 mM Na_3_VO_4_, 10 μl ml^−1^ Protease inhibitor cocktail (PIC, Sigma). Membranes were incubated using specific antibodies for: MDM4 (1:1000, Bethyl, Cat # A700-000, RRID:AB_2631881), mouse MDM4 (1:600, Sigma-Aldrich Cat# M0445, RRID:AB_532256), rabbit mTOR (1:1000, Cell Signaling Technology Cat# 2972, RRID:AB_330978) rabbit phospho p70S6k (Thr 389) (1:1000, Cell Signaling Technology cat #9205, RRID:AB_330944), rabbit p70S6k (1:1000, Cell Signaling Technology Cat# 9202, RRID:AB_331676), mouse Vinculin (1:2000, Millipore Cat# CP74, RRID:AB_2214490), mouse GAPDH (1:10,000, Thermo Fisher Scientific Cat# MA5-15738, RRID:AB_10977387), mouse a-Tubulin (1:4000, Sigma-Aldrich Cat# T9026, RRID:AB_477593). Mouse actin (1:4000, Sigma-Aldrich Cat# A4700, RRID:AB_476730), mouse puromycin (1:500, DSHB Cat# PMY-2A4, RRID:AB_2619605). Membranes were developed using the enhanced chemiluminescence (ECL Amersham, Little Chalfont, UK and Cyanagen, Bologna, IT) by the chemiluminescence imaging system Alliance 2.7 (UVitec Cambridge, UK) and quantified by the software Alliance V_1607.

### Proteomic analysis

Three biological replicates of human SK-OV-3 spheroids and a pool of three biological replicates of murine ID8 spheroids (*Empty Vector* and *MDM4*) were processed. Thirty microgram of proteins of whole-cell extracts lysed in RIPA buffer, for each sample were transferred to a Microcon-10 Centrifugal filter with 10 kDa cutoff (Merck Millipore Ltd.) and followed by FASP digestion protocol^24^. In brief, proteins denatured upon filter aided urea buffer (UB, 8 M urea, 100 mM Tris-HCl, pH=8.5) exchange were reduced by 8 mM DTT (15 min, 56°C) in UB and subsequently alkylated by 0.05 M IAA in UB (20 min, RT, dark). All the samples underwent proteolytic digestion by Trypsin enzyme in 0.05 M ammonium bicarbonate solution, using a protease: protein ratio, 1:50 (w/w), at 37°C overnight. Digested peptides recovered from the filter and blocked by formic acid solution (FA, 0.2% v/v) were concentrated by SpeedVac and resuspended in 0.1% FA. In all, 0.250 µg of each sample was spiked by 500 fmol of Hi3 Rabbit Phosphorylase B Digestion Standard (Hi3 Phos B, Waters Corp.) and run in three technical replicates.

The liquid chromatography-mass spectrometry (LC-MS) analysis was performed on an ACQUITY MClass System directly coupled with a Synapt G2-S*i* mass spectrometer (Waters Corp.). Data have been acquired in High Definition MS^E^ (HDMS^E^), a data-independent acquisition protocol where ion mobility separation has been integrated into LC-MS^E^ workflow, as previously described^25^.

Continuum LC-MS data from three replicate runs for each sample have been processed for qualitative and quantitative analysis using the software ProteinLynx Global Server v. 3.0.3 (PLGS, Waters Corp.). The qualitative identification of proteins has been obtained by searching in the UniProt KB database release of May23_2018, restricted to *Homo Sapiens* or *Mus Musculus* Taxonomy, for SK-OV-3 and ID8 cells respectively, to which the sequence of Hi3 Phosphorylase B from Rabbit (UniProtKB/Swiss-Prot AC: P00489) was appended.

Label-free quantitative analysis was obtained with the protein expression analysis mode integrated into PLGS software (ProteinLynx Global Server, RRID:SCR_016664), using the internal standard for normalization. Filtered tables were generated to include proteins found in at least one of three technical replicates and to exclude proteins showing <30% change (corresponding to a ratio of ±1.3) and those showing no statistical significance according to the PLGS software.

The biofunctional and pathway analyses of the proteins differentially regulated in presence of MDM4 were generated through ingenuity pathway analysis software (IPA) (Ingenuity^®^ Systems, www.ingenuity.com, RRID:SCR_008653).

### Immunofluorescence assays

MCTS/HUVEC (Human Umbilical Vein Endothelial cells)-laden constructs were washed with 1× PBS for 10 min and fixed in 4% PFA for 2 hours. The samples were rinsed with PBS for 1 h, permeabilized in 0.3% TRITON X-100 (Sigma) for 20 min at RT, and incubated in a blocking solution containing 5% bovine serum albumin (BSA, Sigma) for 1 h. The constructs were incubated with primary antibodies diluted 1:100 in 0.5% BSA solution overnight at 4 °C. The primary antibodies used were: mouse monoclonal antibody against MDM4 (OriGene Cat# TA505706, RRID:AB_2623305), sheep monoclonal antibody against Von Willebrand Factor (Abcam Cat# ab11713, RRID:AB_298501). Fluorescein isothiocyanate, tetramethylrhodamine (Jackson Immunoresearch), or Alexa 647 conjugated secondary antibodies were used at 1:150 dilution. Nuclei were stained by 4′,6-diamidino-2-phenylindole. Laser scanning confocal microscopy (Leica Microsystem) was performed to obtain sequential images of labeled structures.

### SUnSET assay

Basal protein translation was measured by using the SUnSET assay as previously described^26^. In brief, ID8 and SK-OV-3 cancer cells were treated with puromycin (10 μg/ml) for 5 or 10 min and then lysed in RIPA buffer (150 mM NaCl, 50 mM Tris-HCl pH 7.4, 1% Triton X-100) containing PIC. Twenty μg of protein were subjected to sodium dodecyl sulphate-polyacrylamide gel electrophoresis and analyzed by western blotting using an anti-puromycin antibody (1:500, DSHB Cat# PMY-2A4, RRID:AB_2619605).

### Statistical analyses

Statistical analysis was carried out using GraphPad (GraphPad Prism, RRID:SCR_002798). Comparisons between the two groups were performed using unpaired Student’s *t* test or one-sample *t* test. One-way or two-way analysis of variance followed by a post hoc Newman–Keuls or Bonferroni’s multiple comparison tests were used when more than two groups were compared. Distributions were analyzed using the Pearson’s chi-square (*χ*^2^) test. Results were presented as mean ± standard deviation of the mean from the indicated number of independent experiments. *P* values > 0.05 were regarded as not significant. The overall survival (OS) of patients with high or low levels of MDM4 or MDM2 was calculated using the KM Plotter (https://kmplot.com). using Affymetrix ID: “205655_at” for MDM4 and “217542_at” for MDM2. In all, 15 y OS in a cohort of 1656 patients with ovarian cancer was derived by the following data sets: GSE14764,GSE15622,GSE18520,GSE19829, GSE23554, GSE26193, GSE26712, GSE27651, GSE30161, GSE3149, GSE51373, GSE62885, GSE65986, GSE9891, TCGA. The patient’s samples were split into two groups using the “auto-select best cutoff” tool. Hazard ratio with 95% confidence interval and log-rank *p* value were calculated, and survival curves were displayed on the webpage.

### Bioprinting assay

Two different methacrylate gelatin (GelMA)—based bioinks were used to generate the bioprinted constructs composed by HUVEC and MCTSs. The first bioink was obtained by dissolving 5% (w/v) GelMA, 4% (w/v) alginate (Alg) in 25 mM Hepes buffer, and resuspending 10 × 10^6^ HUVEC cell/ml. The second bioink was composed of 7% (w/v) GelMA, 4% Alg in 25 mM Hepes buffer, and resuspending 2000 MCTSs/ml. MCTSs were formed using the HD method. Both bioinks were filtered (0.22 μm) before cells encapsulation to guarantee sterile conditions. Irgacure 2959 was added to the bioink as a radical photoinitiator (1 mg/ml) immediately before use. Constructs, characterized by overall dimensions of 8 × 8 × 1 mm^3^, composed of four layers of MCTSs overlaid to four layers of HUVEC, were generated throughout the bioprinting process. The cell-laden scaffolds were bioprinted using a custom microfluidic printing head^27^. The two bioinks and calcium chloride solution (0.3 M) were loaded in three different sterile 1 ml Hamilton glass syringes connected to a dispensing co-axial nozzle system. NE-1000 Single Syringe Pumps (NewEra PumpSystems Inc.) were used to flow the bioinks and the CaCl_2_crosslinking solution (printing speed = 180 mm/min, Q_bioink_ = 5.4 μl/min, Q_CaCl2_ = 5.2 μl/min) obtaining 100 μm diameter fibers (0–90° fiber orientation, 50 μm distance between fibers in the X-Y plane). After bioprinting, the obtained three-dimensional structures were crosslinked by low UV penetration (365 nm, 4–5 mW/cm^2^) for 5 min and washed twice in 4 mM EDTA before cultured in the growth medium.

## Results

### MDM4 reduces EOC cell dissemination

**Fig. 1:**
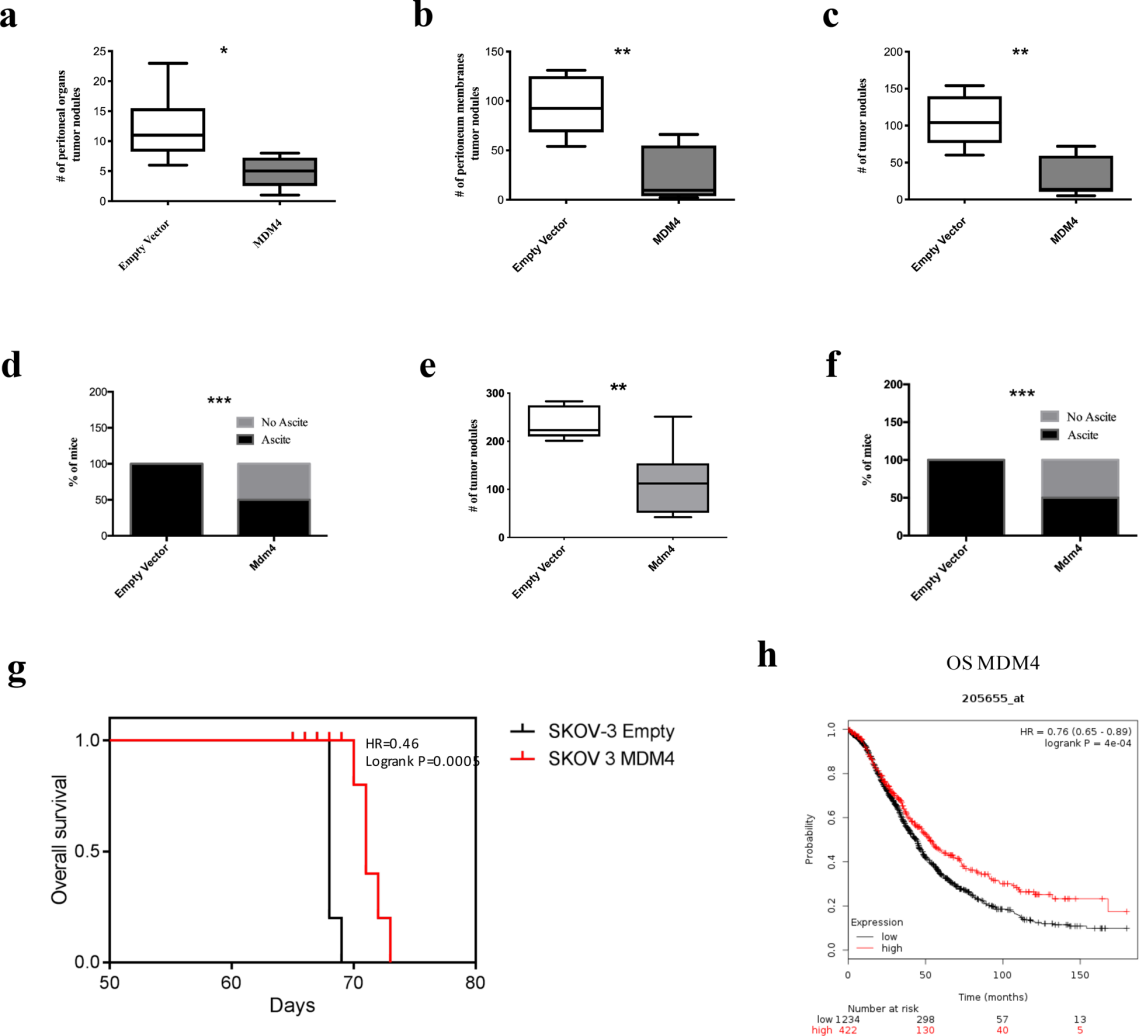
MDM4 decreases ovarian cancer nodules dissemination and improves EOC patients’ overall survival probability. **a** Number of nodules in peritoneal organs of Empty Vector or MDM4-SK-OV-3-injected mice (*n* = 6, two-tailed Student’s *t* test, p = 0.018). **b** Number of peritoneum membranes nodules as in **c** (*n* = 6, two-tailed Student’s *t* test, *p* = 0.0016). **c** Total number of nodules present in both peritoneal organs and peritoneum membranes (*n* = 6, two-tailed Student’s *t* test, *p* = 0.0016). **d** Percentage of mice showing ascites at the final time point (*n* = 6, *χ*^2^ test, *p* < 0.0001). **e** Total number of nodules in *Empty Vector* or *Mdm4*-ID8 cells (*n* = 6 for *Empty Vector*, *n* = 7 for *Mdm4*, two-tailed Student’s *t* test, *p* = 0.0042). Nodules were counted blindly by two independent observers. **f** ID8 ascites analysis as in **d** (*n* = 15, *χ*^2^ test *p* < 0.0001). **g** OS in mice injected with Empty Vector or MDM4-SK-OV-3 cells (*n* = 5, log-rank test, *p* < 0.001). **h** Correlation of MDM4 expression with OS in 1656 patients with epithelial ovarian cancer followed for 15 years (log-rank test, *p* = 0.0004).

To ascertain the function of MDM4 in EOC progression, we analyzed the dissemination of EOC cells by IP injection of the human ovarian cancer cell line SK-OV-3. SK-OV-3 cells^28^, derived from an ovarian adenocarcinoma, do not express p53 and are considered a reliable model to study the metastatic features of EOC cells^29,30^. SK-OV-3 cells were engineered to express human *MDM4* or *Empty Vector* as control (Fig. S1a) and the mCherry marker to visualize in vivo tumor nodules. Cells were IP injected in the NOD/*scid*/gamma (NSG) mice, and 68 days after injection, mice were killed to evaluate the presence of disseminated tumor nodules in peritoneal organs and peritoneum membranes, i.e., on the diaphragm and peritoneal wall. At the necropsy, all mice injected with SK-OV-3 cells showed tumor nodules independently of MDM4 expression (Fig. S1b). However, peritoneal organs showed an average of 12.17 nodules/mouse in *Empty Vector*-SK-OV-3 compared to 4,8 nodules in *MDM4*-expressing mice (Fig. 1a) (60.5% overall decrease). Considering the peritoneum membranes, an average of 94.33 ± 12.03 nodules was found in mice injected with *Empty Vector*-SK-OV-3 cells compared with 23.67 ± 11.29 in those with *MDM4-*SK-OV-3 cells (Fig. 1b, Fig. S1b left panel, black arrows) with a 75% significant decrease. The total number of nodules present in both the peritoneal organs and peritoneum membranes, was on average 106.5 ± 14.11 in *Empty Vector*, and 28.50 ± 7.73 in animals injected with *MDM4-*SK-OV-3 cells (73.2% overall decrease) (Fig. 1c, Fig. S1b right panel, black arrows). Hemorrhagic ascites were observed in 100% of *Empty Vector* and only in 50% of *MDM4*-SK-OV-3 injected mice (Fig. 1d).

Since the presence of an active immune system has been associated with improved clinical outcomes in OC patients^31^, to ascertain the function of MDM4 in an immune-competent background, we utilized allograft of the murine ovarian cancer cell line ID8 in the syngeneic murine background C57BL/6^32^. Similar to SK-OV-3, ID8 cells were engineered to express murine *Mdm4 or Empty Vector* (Fig. S1c) and were IP injected in syngeneic mice. Two months after injection, all mice carrying *Empty Vector-*ID8 or *Mdm4-*ID8 cells showed disseminated tumor nodules (Fig. S1d, black arrows). However, an average of 235.8 ± 13.86 nodules was found in the *Empty Vector* compared with 118.4 ± 27.64 in the *Mdm4*-injected mice (Fig. 1e) with a 49.8% decrease in the number of nodules. Hemorrhagic ascites were found in 15/15 *Empty Vector* and 6/15 *Mmd4* expressing mice (Fig. 1f, Fig. S1d left panel).

Overall, these data demonstrate that high levels of MDM4 significantly reduce the intraperitoneal dissemination of ovarian cancer cells, independently of p53 and an immune-competent background, thus in a cell-autonomous way.

### High *MDM4* levels correlate with increased OS in patients

Since in vivo data demonstrate that MDM4 reduces EOC metastatic potential, we investigated whether this MDM4 activity reflects in disease progression by analyzing the survival of NSG mice implanted with *Empty Vector*- or *MDM4*-SK-OV-3 cells. Indeed, mice injected with *MDM4* showed a significant increase in the OS compared with *Empty Vector* (Fig. 1g). To evaluate in humans whether MDM4 levels impact the survival of EOC patients, we assessed the correlation between OS and *MDM4* mRNA levels in publicly available data sets^33^. To avoid the interference of shorter oncogenic forms of MDM4 also described in ovarian cancer^11,34,35^, we analyzed a probe validated on full-length *MDM4* mRNA (205655_at). Analysis of 1656 patients with EOC showed that 15 years OS probability is significantly increased in patients with high levels of *MDM4* compared to low expressing patients (Fig. 1h). Indeed, the OS probability is doubled in patients that express high levels of MDM4 starting from 6 years after tumor appearance (Fig. 1h). In comparison, the mRNA levels of the *MDM4* analog, *MDM2* do not affect the OS probability of EOC patients (Fig. S1e), confirming the independent positive correlation of MDM4 expression with clinical outcome in patients with EOC.

These data confirm the negative association of MDM4 levels with EOC progression, supporting a potential clinical value of MDM4 expression in this cancer progression.

### MDM4 inhibits cell spreading

**Fig. 2:**
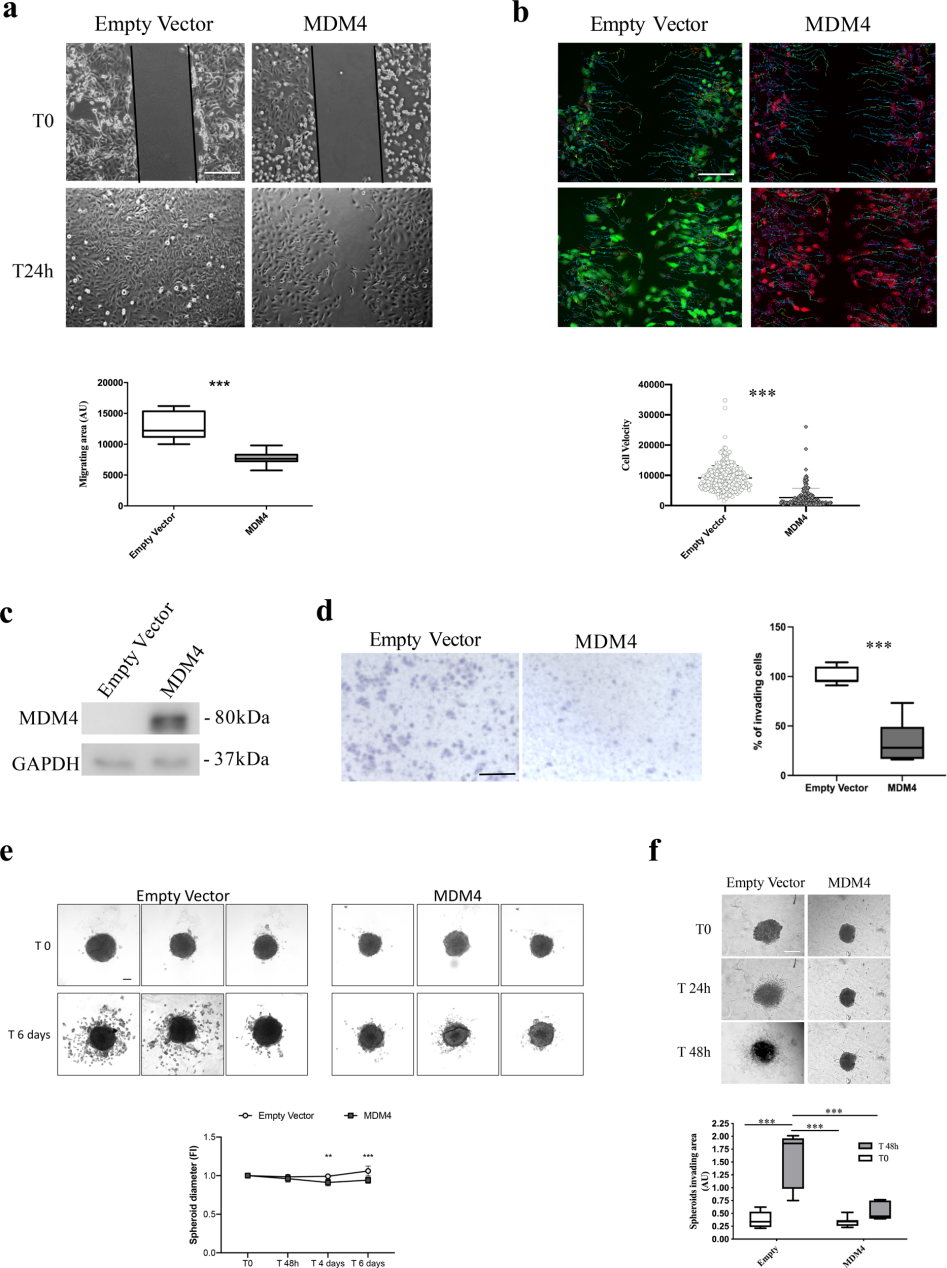
Cell migration and invasion are impaired by high levels of MDM4. **a** Migration of Empty Vector and MDM4-SK-OV-3 cells evaluated through wound-healing assay. Pictures were taken at time 0 and 24 h after the scratch. Pictures are representative of four biological replicates (*n* = 4, two-tailed Student’s *t* test *p* < 0.0001); scale bar = 200 μM. **b** Representative time-lapse micrographs of cell velocity of a mixed population of *Empty Vector* and *MDM4-SK-OV-3* cells labeled with GFP or mCherry, respectively, in a wound-healing migration assay (*n* = 331 cells for Empty Vector and *n* = 177 cells for MDM4, two-tailed Student’s *t* test, *p* < 0,0001); scale bar = 200 μM. **c** Representative WB of MDM4 levels following overexpression in OVCAR-3 cells. **d** Micrographs of transwell cell invasion assay through matrigel by *Empty Vector* or *MDM4-* OVCAR-3 cells scale bar = 50 μM. Right panel shows the quantification (mean ± SD, *n* = 3, two-tailed Student’s *t* test, *p* < 0.0001). **e** Representative images of invading Empty Vector- or MDM4-SK-OV-3 multicellular tumor spheroids (MCTSs) at time 0 and after 6 days of culture. Lower panel, spheroid diameter extrapolated by spheroid area calculated by Visual ImageJ and correlated to t0 area set to 1 (*n* = 7, two-tailed Student *t* test, **p = 0.0041 ***p < 0.0001). **f** Representative time-lapse images of invading Empty Vector- or MDM4-SK-OV-3 MCTSs. The invading area was calculated as follows: total MCTS area at 48 h minus MCTS body area at t0 (*n* = 5, Two-way ANOVA for multiple comparisons, *p* = 0.001); scale bar = 100 μM.

To ascertain the pro-active function of MDM4 in EOC metastatic process, we investigated the activity of MDM4 towards the acquisition of metastatic properties. The metastatic process consists of different steps with cell migration being an early feature of cancer cells to escape from the primary tumor^36^. To understand whether MDM4 affects cell migration, both SK-OV-3 and ID8 cells were subjected to wound-healing assay, a bona fide test to measure migration ability. In fact, both SK-OV-3 (Fig. 2a) and ID8 cells (Fig. S2a) showed reduced migration of *MDM4-* compared with *Empty Vector-*expressing cells. Conversely, silencing of MDM4 in A-2780 (Fig. S2b), an EOC cell line that expresses high levels of MDM4 (Fig. S2b), increased the number of migrating cells supporting the activity of MDM4 in cell migration (Fig. S2c). To understand whether increased cell migration is linked to increased proliferation or reflects intrinsic cell movement ability, we analyzed the velocity of migrating single cells. Indeed, cell migration has been associated with increased cell velocity during their transit and reduced surface friction of the metastatic cells^37^. By using a migration assay with a mixed population of *GFP-Empty Vector* and *mCherry-*MDM4-SK-OV-3 cells grown in the same plate, we calculated the velocity of cells to the wound using the TrackMate Plug-in (Fiji). This parameter was significantly decreased in cells expressing *MDM4* compared with *Empty* Vector-SK-OV-3 cells (Fig. 2b), indicating the ability of MDM4 to reduce cell movement independently of cell proliferation.

Another important characteristic of the EOC metastatic process is the ability to shed from the primary tumor and invade the extracellular matrix. To this purpose, we analyzed cell invasion through the matrigel of *Empty Vector-* and *MDM4-*expressing cells by transwell assay using both ID8 and OVCAR-3 cells another human EOC cell line derived from high-grade serous adenocarcinoma with mutant p53^28^. Cell invasion was significantly impaired in *MDM4*- compared to the *Empty Vector-*expressing OVCAR-3 cells (Fig. 2c, d) and ID8 cells (Fig. S3a). To further confirm these data, we analyzed the ability of EOC cells to form invading cancer cells spheroids, which mimic the ability of EOC to survive and diffuse in the peritoneal fluid and to invade the peritoneal wall^3^. SK-OV-3 and ID8 MCTSs embedded in a collagen Type I matrix formed small and compact MCTSs. Analysis of spheroid diameter indicated an impaired growth of MDM4- compared to Empty vector-expressing MCTS (Fig. 2e). Moreover, MCTS failed to form invading protrusions when expressing *MDM4* whereas, *Empty Vector*-MCTSs formed cell aggregates with invading protrusions in the 3D matrix (Fig. 2e, f and Fig. S3b). These data expand previous findings and confirm that cell invasion is impaired by high levels of MDM4.

**Fig. 3:**
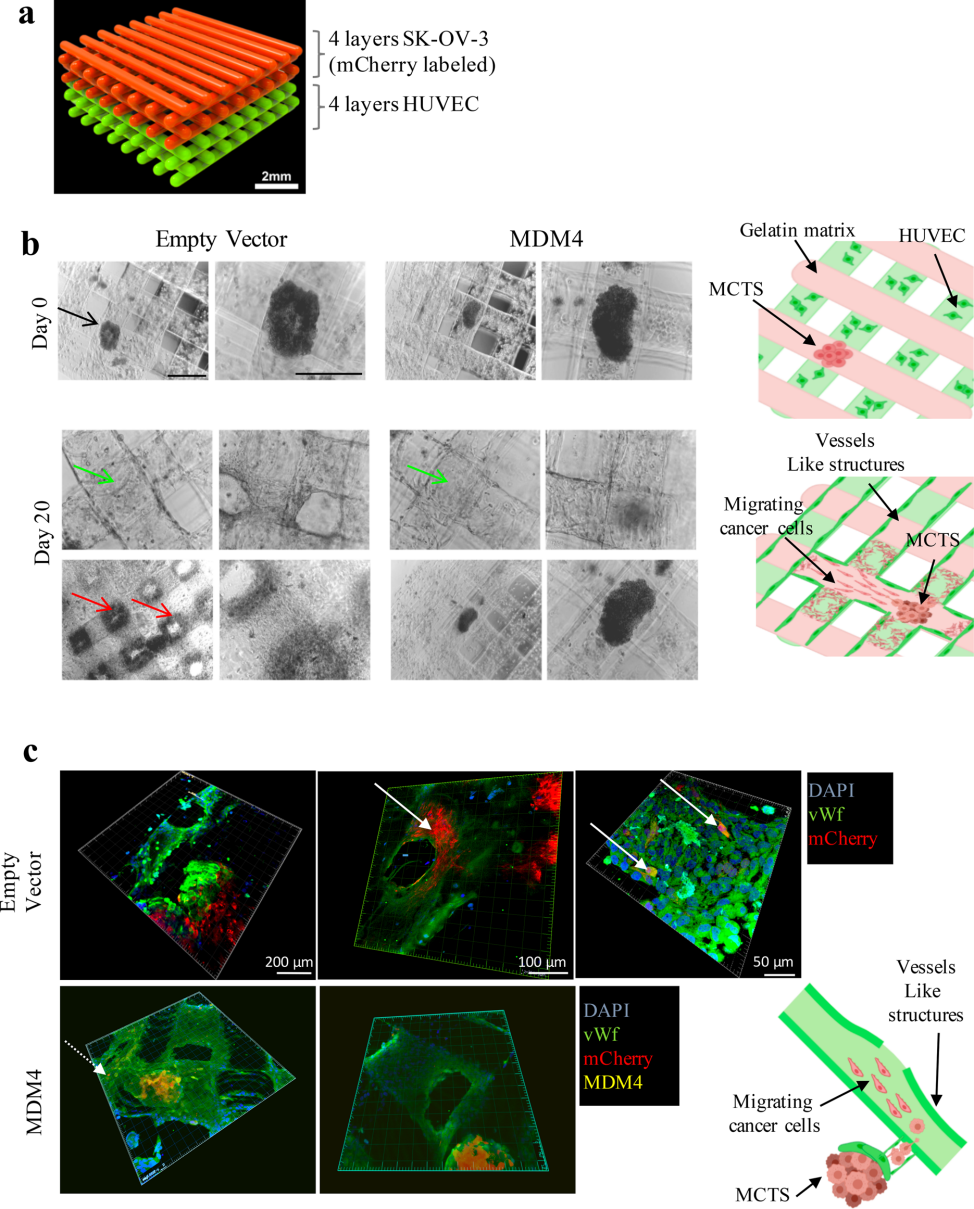
Cell spreading is impaired by MDM4 in a 3D-bioprinting assay. **a** Rendering of the 3D-bioprinting geometry used. **b** Representative pictures of bioprinted constructs carrying MCTS and HUVEC at day 0 (upper panels, black arrow points to the MCTS) and day 20 after bioprinting (lower panels); red and green arrows point to cancer cells spreading and to vessels-like structures, respectively. Right panels show the rendering of day 0 bioprinting strategy (upper panel) and cell invasion at day 20 (lower panel); scale bars = 200 mM. **c** Confocal microscopy of constructs showing the vessels-like structures (stained with sheep anti-Von Willebrand Factor AbCam Cat# ab11713, green signal), cancer cells expressing mCherry (red signal), and MDM4 (stained with Mouse anti-MDM4 Ab OriGene Cat# TA505706, yellow signal), DAPI stains nuclei. White arrows point to migrating cancer cells. The drawing shows the rendering of cancer cells escaping from the MCTS and entering vessels-like structures.

Recent reports indicated that EOC metastatization can occur also through the hematic route, mainly in highly advanced stage disease^4,5^. In particular, SK-OV-3 cells metastasize through both routes in murine models^5^ To test the effects of MDM4 on intravasation ability of EOC cells, we developed an ad hoc 3D-bioprinting assay composed of SK-OV-3 cells in co-culture with HUVEC. Bioprinting assay has been amply used as in vitro model for intravasation of normal and cancer cells^38–40^ with the use of HUVEC as they form an endothelial layer with multiple branches when bioprinted^41^. Specifically, we generated a model whereby MCTSs, and no single cells, were extrusion-bioprinted in a gelatin-alginate hydrogel. Moreover, MCTSs were layered in fibers different from those of HUVEC, to mimic more closely the in vivo environment in which cancer cells escape from MCTSs, disseminate through the matrix, and intravasate in HUVEC-formed vessels to spread to distant sites. Scaffolds (8 × 8 × 1 mm^3^) were generated with four layers of MCTSs overlaid to four layers of HUVEC cells in a 4:4 geometry (Fig. 3a). On day 0, MCTSs formed by *Empty Vector-* or *MDM4*-SK-OV-3 cells labeled with mCherry, were present at the top layers where they have been bioprinted (Fig. 3b, day 0). Analysis of constructs at day 20, revealed the formation by HUVEC of tubular structures, although incomplete, resembling vessels formation (Fig. 3b, day 20 green arrows, and Fig. 3c, green-labeled structures). Of note, *mCherry-Empty Vector-*SK-OV-3 cells showed a wide-spreading all over the scaffolds (Fig. 3b, day 20 red arrows, and Fig. 3c, *Empty Vector* white arrows). Conversely, only one *mCherry-MDM4* MCTS showed few cells spreading (Fig. 3c, MDM4 white dotted arrow), whereas all other MCTS remained compact. Notably, some *Empty Vector*-SK-OV-3 cells were observed inside the lumen of the tubular structures (Fig. 3c, upper panels, white arrows), indicating their ability to spread through vessels analogously to in vivo cancer cell intravasation. No mCherry*-MDM4*-SK-OV-3 cells were observed inside the lumen of tubular structures (Fig. 3c, right lower panel).

This 3D-bioprinting assay confirmed the ability of *MDM4* to reduce EOC cell migration and dissemination and also to impair intravasation. Taken together, these data demonstrate that high expression of MDM4 inhibits ovarian cancer cell spreading by affecting the early features of the EOC metastatic cells, migration, and invasion.

### mRNA translation is impaired in the presence of high levels of MDM4

To understand the molecular mechanism underneath the activity of MDM4 in EOC progression, we performed a whole proteomic analysis of the human and murine EOC cell lines. Since 3D culture better represents the in vivo metastatic niche compared with 2D cell culture, *Empty Vector-* and *MDM4*-SK-OV-3 or -ID8 cells were grown as pending drops to form MCTSs. After 18 hours, MCTSs were collected and subjected to quantitative proteomic analysis using a comparative label-free shotgun proteomic approach.

**Fig. 4:**
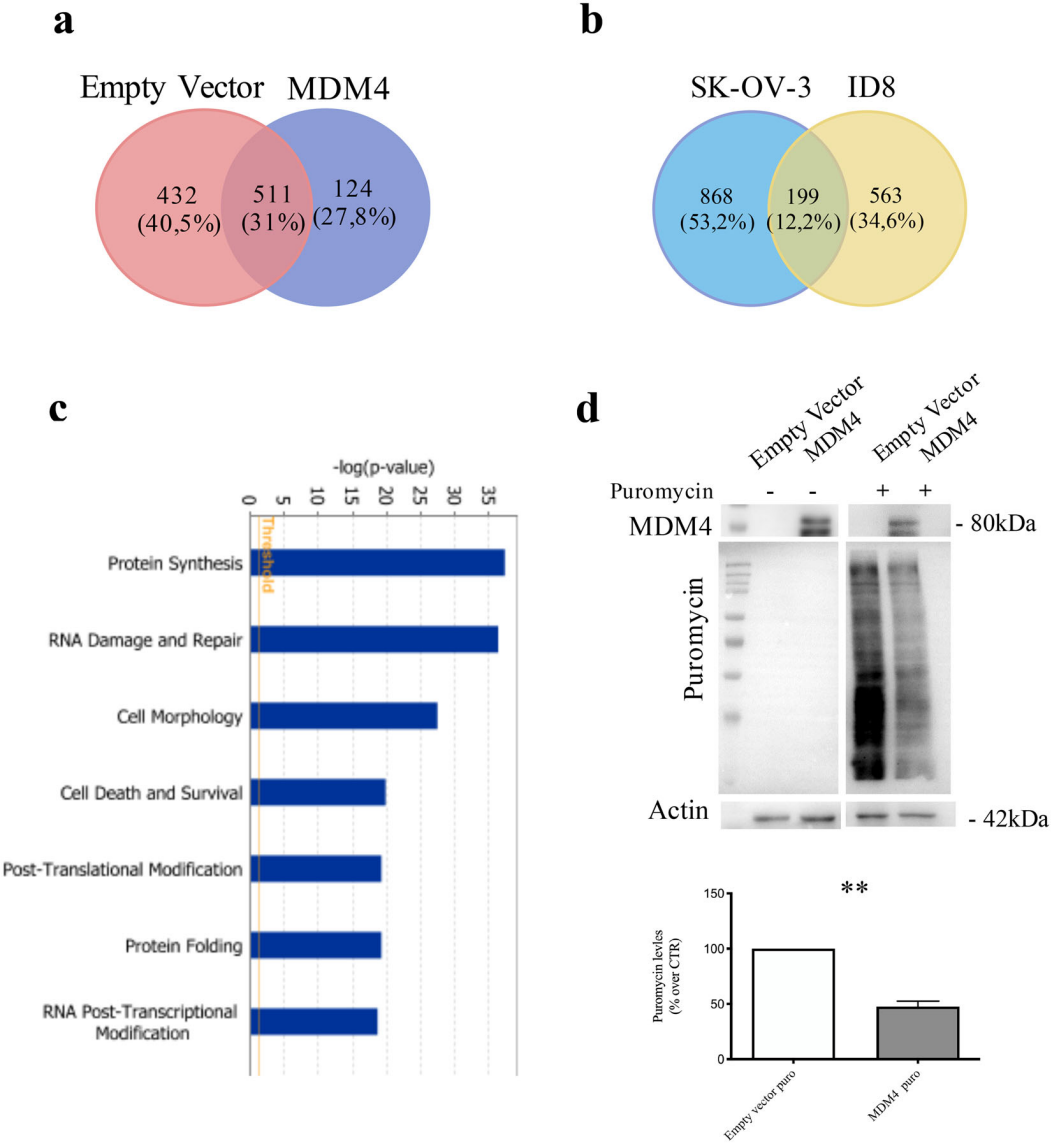
MDM4 overexpression leads to a decreased generalized protein translation. **a** Venn diagram showing the overlap of protein expressed in Empty Vector- and MDM4-SK-OV-3 cells. **b** Venn diagram showing common proteins between SK-OV-3 and ID8 cells overexpressing MDM4. **c** IPA biofunctional analysis of the proteins differently expressed in MDM4-SK-OV-3 cells compared to Empty Vector. **d** SunSet assay of Empty Vector- and MDM4-SK-OV-3 cells; representative WB analysis of indicated proteins following indicated treatments (upper panel), quantification of three independent assays (lower panel) (*n* = 3, one-sample *t* test *p* = 0.009).

A total of 1741 proteins were identified in *Empty Vector-* and *MDM4*-SK-OV-3 cells with 1067 proteins differently regulated upon *MDM4* overexpression (Fig. 4a, Table S1). In ID8 cells a total of 1615 proteins were identified with 762 proteins significantly modulated in *MDM4* vs *Empty* Vector-expressing cells (Fig. S4a, Table S2). More than 10% of proteins were common in two cell lines (Fig. 4b), suggesting a shared signature underlying MDM4 activity. Indeed, biofunctional analysis using IPA revealed that the main bio-function significantly affected by the expression of MDM4 was the “Protein synthesis” in both SK-OV-3 and ID8 cell lines (Fig. 4c and Fig. S4b), in agreement with a recent report model highlighting altered control of mRNA translational as a critical factor in cancer development and progression, also in OC^42,43^.

To validate these data and test the translation status of ovarian cancer cell lines expressing different levels of *MDM4*, we performed a SunSet assay^26^, an alternative method to classical radioactive labeling to study mRNA translation. Incorporation of puromycin, a structural analog of aminoacyl tRNA, into nascent polypeptides results in the termination of peptide elongation, reflecting rate of mRNA translation. Noteworthy, we observed a significant reduction in protein synthesis in puromycin-treated *MDM4-* compared with *Empty Vector*-SK-OV-3 cells (Fig. 4d), confirmed also in the ID8 cell line (Fig. S4c).

These data indicate that MDM4 represses protein translation, and suggest that this regulation can underlie the reduced metastatic ability conferred to EOC cells by high *MDM4* levels.

### MDM4 affects the mTOR-regulated pathway

**Fig. 5:**
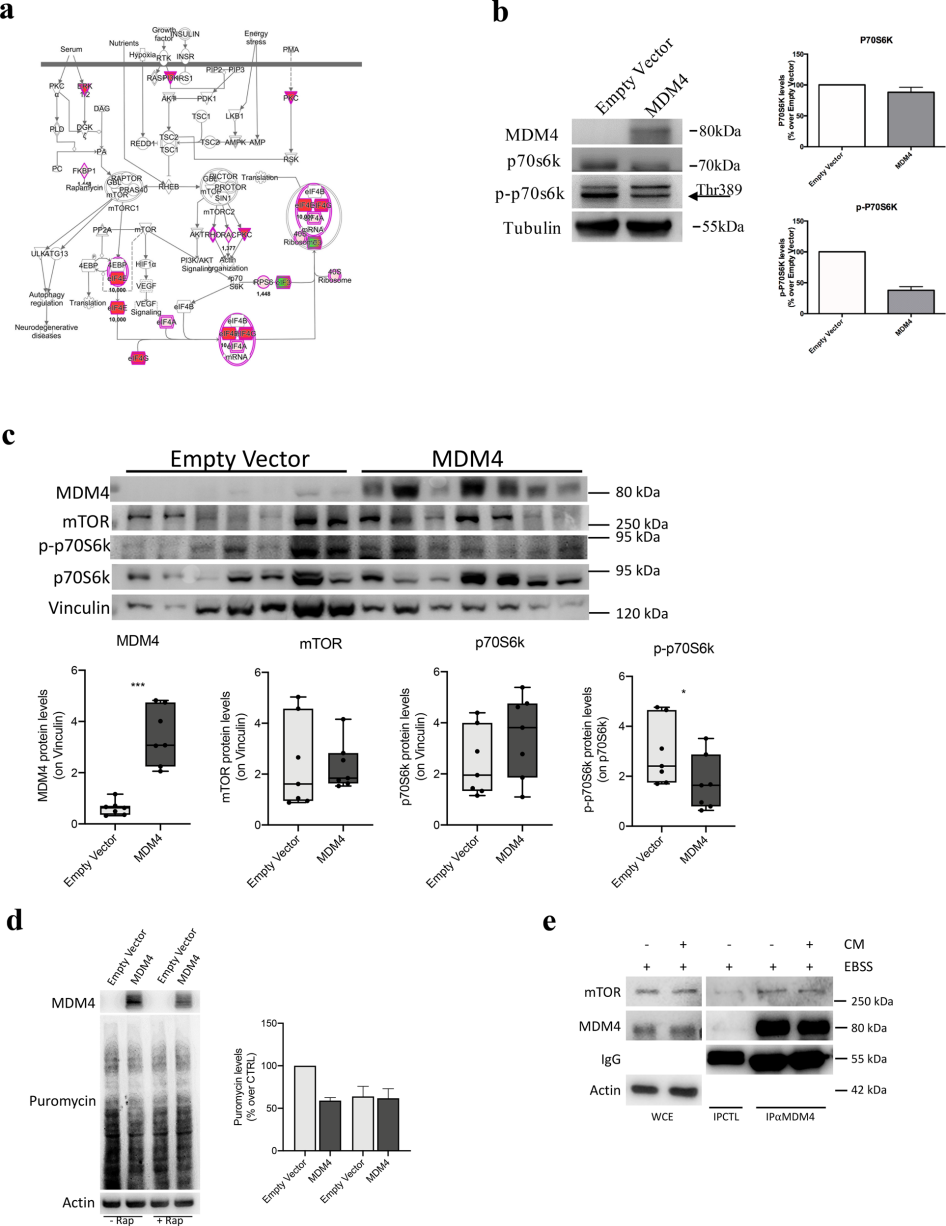
MDM4 expression affects mTOR activation. **a** Pathway analysis of mTOR signaling in SK-OV-3 cells. Red and green colors underlie proteins differently expressed in MDM4- compared with Empty vector-SK-OV-3 cells (red = upregulated, green = downregulated proteins). **b** WB of Empty Vector or MDM4-SK-OV-3 cells showing S6K1 and p70S6K1^T389^ expression (left panel) and quantification (right panel) (*n* = 3, one-sample *t* test *p* < 0.001). **c** WB of indicated proteins in tumor nodules (one per mouse) generated by IP injection of Empty Vector or MDM4-SK-OV-3 cells in mice; lower panels show quantification (*n* = 7 mice, two-sided Student’s *t* test, ***p < 0.001, *p = 0.048). **d** SunSet assay of Empty Vector- and MDM4-SK-OV-3 cells treated with rapamycin for 6 hours; quantification of two independent assays (right panel). **e** WB of indicated prot**e**ins in whole-cell extract (WCE) and co-immunocomplex from SK-OV-3 cells grown in EBSS for 1 hour and then in absence or presence of complete medium for additional 1 hour (CM). 1 mg of WCE was immunoprecipitated with anti-MDM4 antibody (IPαMDM4) or control Ig (IPCTL). Right panel shows the analysis of 1/100 of WCE.

To understand how global repression of protein synthesis by MDM4 triggers reduction of metastatic properties, we analyzed the molecules that underlie the altered bio-function previously shown. IPA analysis revealed that among the main molecular pathways perturbed upon high expression of MDM4 there were “mTOR Signaling” and “Regulation of eIF4 and p70S6K Signaling” besides the “EIF2 signaling” (Table S3). As the mTOR pathway is an important regulator of translation initiation^44^ (Fig. 5a, Fig. S4d) that also converges in cell migration^45^, we hypothesized that mTOR inactivity underlies the repression of metastatic features by MDM4. Furthermore, the serine/threonine kinase p70 ribosomal S6 kinase 1 (S6K1), a major mTOR target, has a crucial role in the metastatic process^46^ also in ovarian cancer^47^. To confirm and expand proteomic data, the activation of the mTOR/S6K1 pathway was evaluated analyzing the levels of phosphorylated p70S6K1^T389^ in the EOC cell line. Indeed, we found a significant reduction of p70S6K1^T389^ in presence of MDM4 compared to Empty Vector in SK-OV-3 cells (Fig. 5b). Importantly, these data were confirmed by analysis of in vivo metastases derived from different *Empty Vector-* and *MDM4*-SK-OV-3 injected mice (Fig. 5c). Conversely, mTOR levels were not affected (Fig. 5c), supporting the impairment of its activity by MDM4. To confirm that MDM4-mediated inhibition of protein synthesis is mediated by mTOR, we repeated the SunSet assay in the presence of the mTOR inhibitor, rapamycin. To avoid a strong block of protein synthesis, cells were analyzed 6 hours after the treatment. Rapamycin reduced protein synthesis in agreement with its inhibitory activity (Fig. 5d). Of interest, in the presence of this drug, MDM4 did not further reduce puromycin signals compared to Empty vector, supporting that MDM4 effects on protein synthesis are mediated by mTOR function (Fig. 5d). Given our previous finding that MDM4 inhibits mTOR by binding to it^19^, we analyzed these two proteins’ association. Indeed, MDM4 co-immunoprecipitates endogenous mTOR in these cells too independently of cell growth conditions, further supporting the functional relationship between mTOR and MDM4 (Fig. 5e).

**Fig. 6:**
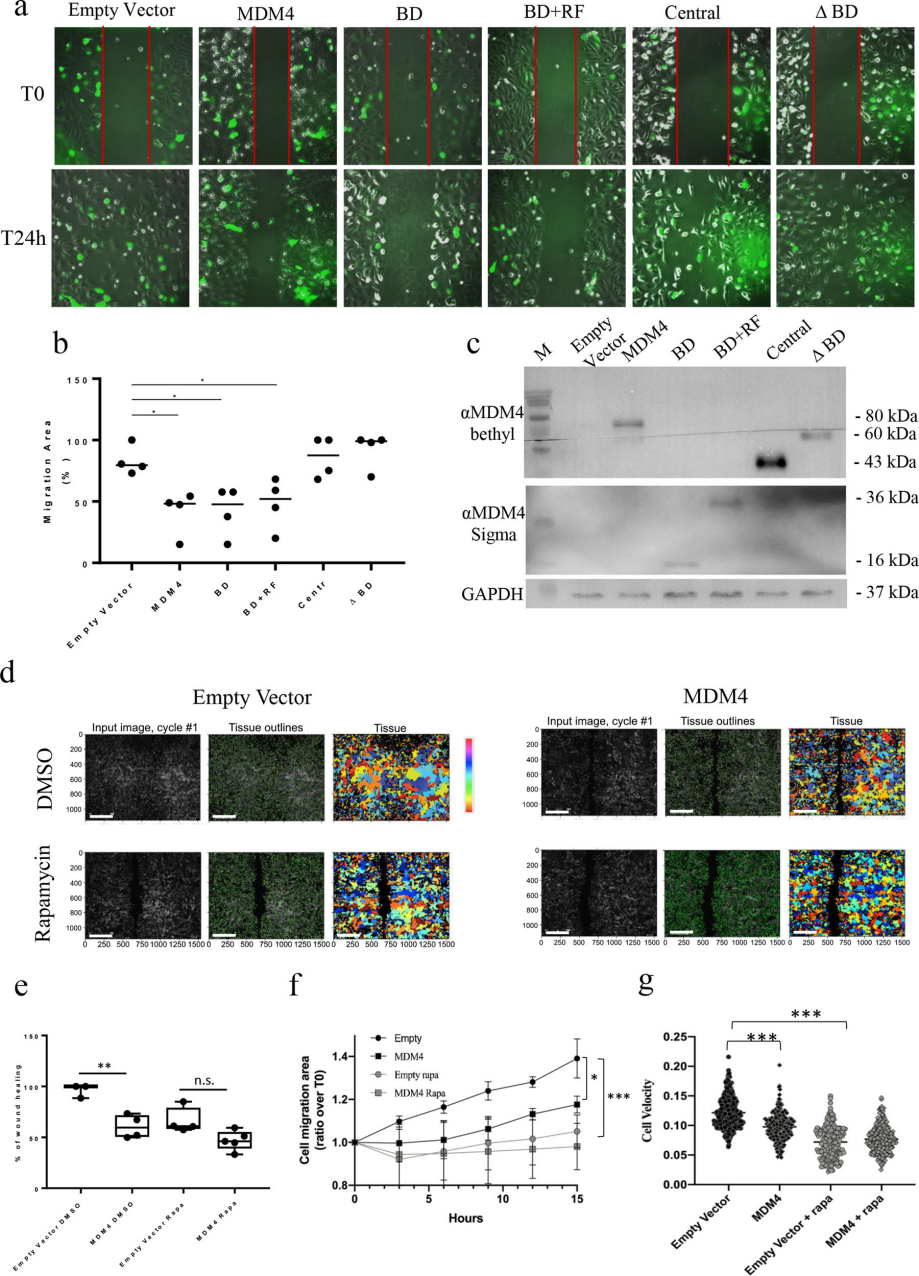
mTOR is required for MDM4 activity on cell migration. **a** Upper panel: representative pictures of cell migration evaluated through wound-healing assay of Empty Vector- or SK-OV-3 cells expressing wt or MDM4 deletion mutants+GFP as indicated. Pictures were taken at time 0 and 24 h after the scratch. Scale bar = 200 Mμ **b** quantification of cell migration (*n* = 4, one-way ANOVA for multiple comparisons, Empty vector vs wt-MDM4, *p* = 0.0135; Empty vector vs BD, *p* = 0.015; Empty vector vs BD + RF, *p* = 0.04). BD = p53-binding domain, RF = Ring Finger domain, Central = Central domain- lacking both BD and RF domains. **c** WB of MDM4 deletion mutants used in **a**. **d** Representative images of cell migration of Empty Vector and MDM4-SK-OV-3 cells using Rapamycin (Rapa) or vehicle (DMSO). Representative micrographs show the analysis performed with Cell Profiler 3.1.9, 15 h after scratching. Colors are randomly assigned by the software. **e** Quantification of cell migration shown in **c** using Fiji (*n* = 4, one-way ANOVA for multiple comparisons, Empty Vector+DMSO vs MDM4 + DMSO, *p* = 0.004; Empty Vector+DMSO vs Empty vector+Rapa, *p* = 0.01; Empty Vector+DMSO vs MDM4 + Rapa, *p* = 0.0002). **f** Quantification of cell confluency shown in **c** (one-way ANOVA for multiple comparisons, Empty Vector+DMSO vs MDM4 + DMSO, *p* = 0.04; Empty Vector+DMSO vs Empty vector+Rapa, *p* = 0.001; Empty Vector+DMSO vs MDM4 + Rapa, *p* = 0.0003). **g** Cell velocity analysis of cell migration shown in **c** (*n* = 392 cells for Empty Vector+DMSO, *n* = 229 cells for MDM4 + DMSO, *n* = 170 cells for Empty Vector+Rapa, *n* = 232 for MDM4 + Rapa, one-way ANOVA for multiple comparisons, Empty Vector+DMSO vs MDM4 + DMSO, *p* < 0.0001; Empty Vector+DMSO vs Empty vector+Rapa, *p* < 0.0001).

To confirm that MDM4-mediated inhibition of mTOR underlies the reduction of metastatic features, we tested cell migration following expression of MDM4 deletion mutants that do not bind mTOR, according to published data^19^. All the mutants lacking the N-terminal domain (MDM4-ΔBD and MDM4-Central), through which the two proteins interact^19^, did not inhibit SK-OV-3 migration (Fig. 6a–c; Fig. S5a). To further establish the involvement of mTOR in MDM4 activity, we tested the ability of MDM4 to impair SK-OV-3 migration in presence of rapamycin. Indeed, this drug, by suppressing the mTOR-mediated S6K1 pathway, reduces cell motility^48^. Accordingly, rapamycin strongly reduced cell migration and velocity of *Empty*-SK-OV-3 cells and MDM4-expressing cells (compare Rapa vs. DMSO for each cell population, Fig. 6d–g; Fig. S5b). Interestingly, under this treatment MDM4 did not significantly reduce cell migration or velocity compared to Empty vector (Fig. 6d–g compare Rapa-treated *MDM4* with Rapa-treated *Empty Vector*), indicating the requirement of active mTOR for MDM4 function. Time-course analysis confirmed the similar behavior of rapamycin-treated *Empty Vector*-SK-OV-3 and *MDM4* cells (Fig. 6f; Video S1). Of note, rapamycin treatment reduced cell migration and velocity of *MDM4*-SK-OV-3 substantially less compared to *Empty vector*-cells (Fig. 6c–f; Fig. S5b), confirming the impairment of mTOR by MDM4 in these cells and suggesting that high levels of MDM4 may reduce the efficacy of mTOR therapeutic inhibition in EOC. Overall, these data demonstrate that MDM4 inhibits EOC metastatic properties and that this occurs through inactivation of the mTOR-mediated protein synthesis.

## Discussion

In this study, we demonstrated that high levels of MDM4 reduce the metastatic potential of EOC cells in xenograft and allograft mouse models. By using 2D and 3D assays, we showed that MDM4 affects the early steps of the EOC metastatic process, reducing migration and spreading of tumor cells. Moreover, a bioprinting co-culture assay showed that cells overexpressing MDM4 are unable to diffuse and intravasate into HUVEC-formed vessels. Overall, this evidence supports a model whereby MDM4 levels are an important factor in restraining metastatic properties of EOC. Importantly, these data are supported by the positive association of *MDM4* levels with OS in a large subset of patients with EOC.

This “oncosuppressive” activity of MDM4 may appear in contrast with its in vivo oncogenic function^9,10,49^. The most striking difference with previous studies is that MDM4 “oncosuppressive” activity here described is p53-independent. Accordingly, a recent report indicated that high levels of MDM4 may support the therapeutic application of anti-cancer ferroptosis inducers independently of p53^18^. Moreover, MDM4 may possess context-dependent tumor suppressor functions in addition to its well reported oncogenic function^50^.

More recently, we have also demonstrated the importance of MDM4 subcellular localization to determine its activity^9^; specifically, the nuclear localization of MDM4 appears to counteract its oncosuppressive function. In fact, MDM4 shows almost exclusive nuclear localization in human tumors^51^, whereas the protein is mainly cytoplasmic in non-tumor cells^52^. The relationship evidenced in this work between MDM4 and mTOR, which is a cytoplasmic factor, further supports this model and highlights the relevance of detecting subcellular localization to deeply understand this protein function and its usefulness for diagnostic and therapeutic purposes^53^.

The precocious antagonistic activity of MDM4 on the acquisition of the EOC cells metastatic features is supported by results of the proteomic analysis, which hint at general repression of protein synthesis associated with MDM4 expression. Dysregulation of translation initiation has been thoroughly documented in human cancer, included EOC^54^, and a general derepression of this process has been associated with cancer progression and acquisition of the metastatic features^55^.

The translation machinery is tightly controlled by phosphatidylinositol 3-kinase (PI3K)/AKT/mTOR signaling pathway with mTOR promoting translation of components of the translation machinery. Indeed, MDM4 impairs mTOR function by downregulating its target p70S6K1^T389^. The inability of MDM4 to reduce protein synthesis in the presence of rapamycin further highlights the crosstalk between two proteins. In addition, the proof of their binding and the requirement of this binding by MDM4 to inhibit EOC cell migration support a model whereby cytoplasmic MDM4 by binding to mTOR and inhibiting its activity, reduces protein synthesis and consequently cell migration and invasion properties. Of note, recent work demonstrated that deregulation of protein translation associated with mTOR upregulation promotes breast cancer metastasis^56^. Importantly, the ability of MDM4 to inhibit mTOR activity as previously reported^19,20^, demonstrates its potential clinical relevance by affecting EOC metastatic properties. Intriguingly, the MDM4-binding region to mTOR superimposes p53-binding region, that is the NH_2_ terminal domain, suggesting a competition between p53-dependent and independent MDM4 activities in tumor cells.

Different studies have reported hyperactivation and mutation of the mTOR pathway in OC^43^. Accordingly, various clinical trials with mTOR inhibitors have been initiated, gaining, unfortunately, limited success. The results of our study may provide a molecular rationale for this limited success: impairment of mTOR activity by the levels of external regulators as MDM4, can reduce the efficacy of pharmacological inhibition. Analysis of MDM4 levels may, therefore, provide a more detailed framework for the application of the mTOR-targeted therapies.

Overall, these data indicate that high MDM4 levels inhibit the EOC metastatic process contributing to restrain cancer progression and suggest that evaluation of its levels can be a useful marker for prognostic and therapeutic purposes in EOC.

## Supplementary information

Supplementary Table S1

Supplementary Table S2

Supplemental Figures

Supplementary Video 1

## Data Availability

Proteomics data are deposited on the http://www.ebi.ac.uk/pride with the accession number PXD019935.
