# Supplementary material for: Inhibition of the mTOR pathway and reprogramming of protein synthesis by MDM4 reduce ovarian cancer metastatic properties
Source: Cell Death Dis. 2021 May 29;12(6):558. doi: 10.1038/s41419-021-03828-z (PMC8164635; doi:10.1038/s41419-021-03828-z)
Supplement: Supplementary file 1 — Supplementary Table S1 [file 41419_2021_3828_MOESM1_ESM.pdf]

Table S1. MDM4-SK-OV-3 vs Empty Vector-SK-OV-3 MCTSs

Accession * Unique protein sequence identifier according to UniProtKB/Swiss-Prot Protein Knowledgebase, release 2011_06 of 31-May-11.

PLGS Score§ ProteinLynx Global Server score.

MDM4/EMPTY Ratio§§ Ratio of expression between the two experimental groups. An arbitrary ratio value of 10 or 0.1 is attributed to a protein found “highly represented” in the numerator (MDM4) or in the denominator (Empty) of the fraction, respectively.

	Accession *	Description	PLGS Score§	MDM4/EMPTY Ratio§§
1	Q59GN2	R39L5_HUMAN Putative 60S ribosomal protein L39-like 5 OS=Homo sapiens OX=9606	1654,71	10
2	O00410	IPO5_HUMAN Importin-5 OS=Homo sapiens OX=9606 GN=IPO5 PE=1 SV=4	251,59	10
3	Q5XKP0	MIC13_HUMAN MICOS complex subunit MIC13 OS=Homo sapiens OX=9606 GN=MIC1	146,18	10
4	Q03468	ERCC6_HUMAN DNA excision repair protein ERCC-6 OS=Homo sapiens OX=9606 GN=E	209,71	10
5	Q96HC4	PDLI5_HUMAN PDZ and LIM domain protein 5 OS=Homo sapiens OX=9606 GN=PDLIM	448,58	10
6	P31689	DNJA1_HUMAN DnaJ homolog subfamily A member 1 OS=Homo sapiens OX=9606 GN	251,81	10
7	P0C7P4	UCRIL_HUMAN Putative cytochrome b-c1 complex subunit Rieske-like protein 1 OS=H	182,55	10
8	O43765	SGTA_HUMAN Small glutamine-rich tetratricopeptide repeat-containing protein alpha	261,98	10
9	Q9UBQ7	GRHPR_HUMAN Glyoxylate reductase/hydroxypyruvate reductase OS=Homo sapiens (	306,68	10
10	Q9Y3F4	STRAP_HUMAN Serine-threonine kinase receptor-associated protein OS=Homo sapier	259,59	10
11	Q13510	ASAH1_HUMAN Acid ceramidase OS=Homo sapiens OX=9606 GN=ASAH1 PE=1 SV=5	245,21	10
12	P67870	CSK2B_HUMAN Casein kinase II subunit beta OS=Homo sapiens OX=9606 GN=CSNK2B	231,9	10
13	Q13506	NAB1_HUMAN NGFI-A-binding protein 1 OS=Homo sapiens OX=9606 GN=NAB1 PE=1 S	280,5	10
14	Q13501	SQSTM_HUMAN Sequestosome-1 OS=Homo sapiens OX=9606 GN=SQSTM1 PE=1 SV=1	185,13	10
15	O43747	AP1G1_HUMAN AP-1 complex subunit gamma-1 OS=Homo sapiens OX=9606 GN=AP1	243,13	10
16	Q96DY7	MTBP_HUMAN Mdm2-binding protein OS=Homo sapiens OX=9606 GN=MTBP PE=1 SV	458,47	10
17	P11171	41_HUMAN Protein 4.1 OS=Homo sapiens OX=9606 GN=EPB41 PE=1 SV=4	256,23	10
18	P55263	ADK_HUMAN Adenosine kinase OS=Homo sapiens OX=9606 GN=ADK PE=1 SV=2	197,28	10
19	Q12774	ARHG5_HUMAN Rho guanine nucleotide exchange factor 5 OS=Homo sapiens OX=960	282,33	10

20	O15360	FANCA_HUMAN Fanconi anemia group A protein OS=Homo sapiens OX=9606 GN=FAN	109,34	10
21	Q5JQF8	PAP1M_HUMAN Polyadenylate-binding protein 1-like 2 OS=Homo sapiens OX=9606 G	464,79	10
22	Q99865	SPI2A_HUMAN Spindlin-2A OS=Homo sapiens OX=9606 GN=SPIN2A PE=1 SV=3	259,05	10
23	Q6RW13	ATRAP_HUMAN Type-1 angiotensin II receptor-associated protein OS=Homo sapiens C	218,47	10
24	P11166	GTR1_HUMAN Solute carrier family 2_ facilitated glucose transporter member 1 OS=H	313,21	10
25	Q6P1N0	C2D1A_HUMAN Coiled-coil and C2 domain-containing protein 1A OS=Homo sapiens O	252,1	10
26	Q5PSV4	BRM1L_HUMAN Breast cancer metastasis-suppressor 1-like protein OS=Homo sapiens	162,04	10
27	Q9BYZ2	LDH6B_HUMAN L-lactate dehydrogenase A-like 6B OS=Homo sapiens OX=9606 GN=LD	423,71	10
28	Q8TCY9	URGCP_HUMAN Up-regulator of cell proliferation OS=Homo sapiens OX=9606 GN=UR	287,35	10
29	Q9Y3C6	PPIL1_HUMAN Peptidyl-prolyl cis-trans isomerase-like 1 OS=Homo sapiens OX=9606 G	206,97	10
30	O15347	HMGB3_HUMAN High mobility group protein B3 OS=Homo sapiens OX=9606 GN=HM	246,22	10
31	O94979	SC31A_HUMAN Protein transport protein Sec31A OS=Homo sapiens OX=9606 GN=SEC	188,32	10
32	Q9Y3B4	SF3B6_HUMAN Splicing factor 3B subunit 6 OS=Homo sapiens OX=9606 GN=SF3B6 PE	307,65	10
33	Q9Y3B3	TMED7_HUMAN Transmembrane emp24 domain-containing protein 7 OS=Homo sapi	182,16	10
34	O43716	GATC_HUMAN Glutamyl-tRNA(Gln) amidotransferase subunit C_ mitochondrial OS=Ho	577,64	10
35	Q14919	NC2A_HUMAN Dr1-associated corepressor OS=Homo sapiens OX=9606 GN=DRAP1 PE	189,1	10
36	Q9UHX1	PUF60_HUMAN Poly(U)-binding-splicing factor PUF60 OS=Homo sapiens OX=9606 GN	252,22	10
37	Q10567	AP1B1_HUMAN AP-1 complex subunit beta-1 OS=Homo sapiens OX=9606 GN=AP1B1	245,27	10
38	P11498	PYC_HUMAN Pyruvate carboxylase_ mitochondrial OS=Homo sapiens OX=9606 GN=PC	178,37	10
39	Q7KZF4	SND1_HUMAN Staphylococcal nuclease domain-containing protein 1 OS=Homo sapien	431,78	10
40	P29017	CD1C_HUMAN T-cell surface glycoprotein CD1c OS=Homo sapiens OX=9606 GN=CD1C	245	10
41	Q9NNZ3	DNJC4_HUMAN DnaJ homolog subfamily C member 4 OS=Homo sapiens OX=9606 GN	684,28	10
42	P29373	RABP2_HUMAN Cellular retinoic acid-binding protein 2 OS=Homo sapiens OX=9606 GI	178,05	10
43	P29372	3MG_HUMAN DNA-3-methyladenine glycosylase OS=Homo sapiens OX=9606 GN=MP	212,29	10
44	Q1L5Z9	LONF2_HUMAN LON peptidase N-terminal domain and RING finger protein 2 OS=Hom	112,99	10
45	P30520	PURA2_HUMAN Adenylosuccinate synthetase isozyme 2 OS=Homo sapiens OX=9606 (	234,48	10
46	Q96NG3	TTC25_HUMAN Tetratricopeptide repeat protein 25 OS=Homo sapiens OX=9606 GN=T	310,79	10
47	Q9UHV9	PFD2_HUMAN Prefoldin subunit 2 OS=Homo sapiens OX=9606 GN=PFDN2 PE=1 SV=1	777,24	10
48	O14579	COPE_HUMAN Coatomer subunit epsilon OS=Homo sapiens OX=9606 GN=COPE PE=1	267,25	10
49	Q8N4J0	CARME_HUMAN Carnosine N-methyltransferase OS=Homo sapiens OX=9606 GN=CAR	239,74	10
50	Q96E39	RMXL1_HUMAN RNA binding motif protein_ X-linked-like-1 OS=Homo sapiens OX=960	258,79	10

51	Q96GX2	A7L3B_HUMAN Ataxin-7-like protein 3B OS=Homo sapiens OX=9606 GN=ATXN7L3B PE	310,09	10
52	O75525	KHDR3_HUMAN KH domain-containing_ RNA-binding_ signal transduction-associated	222,72	10
53	O75884	RBBP9_HUMAN Putative hydrolase RBBP9 OS=Homo sapiens OX=9606 GN=RBBP9 PE=	481	10
54	P07910	HNRPC_HUMAN Heterogeneous nuclear ribonucleoproteins C1/C2 OS=Homo sapiens	346,48	10
55	O94921	CDK14_HUMAN Cyclin-dependent kinase 14 OS=Homo sapiens OX=9606 GN=CDK14 P	84,84	10
56	A0A1B0GUJ8	PNM8C_HUMAN Paraneoplastic antigen-like protein 8C OS=Homo sapiens OX=9606 G	315,51	10
57	Q9UKY7	CDV3_HUMAN Protein CDV3 homolog OS=Homo sapiens OX=9606 GN=CDV3 PE=1 SV	302,03	10
58	Q96E14	RMI2_HUMAN RecQ-mediated genome instability protein 2 OS=Homo sapiens OX=96	226,1	10
59	Q96AJ1	CLUA1_HUMAN Clusterin-associated protein 1 OS=Homo sapiens OX=9606 GN=CLUAI	280,5	10
60	P48729	KC1A_HUMAN Casein kinase I isoform alpha OS=Homo sapiens OX=9606 GN=CSNK1A	386,94	10
61	Q9Y2Z4	SYYM_HUMAN Tyrosine--tRNA ligase_ mitochondrial OS=Homo sapiens OX=9606 GN=	349,66	10
62	O94905	ERLN2_HUMAN Erlin-2 OS=Homo sapiens OX=9606 GN=ERLIN2 PE=1 SV=1	234,68	10
63	Q9BSE5	SPEB_HUMAN Agmatinase_ mitochondrial OS=Homo sapiens OX=9606 GN=AGMAT PE	224,56	10
64	P55884	EIF3B_HUMAN Eukaryotic translation initiation factor 3 subunit B OS=Homo sapiens O	468,82	10
65	Q9UBE0	SAE1_HUMAN SUMO-activating enzyme subunit 1 OS=Homo sapiens OX=9606 GN=SA	283,21	10
66	P47985	UCRI_HUMAN Cytochrome b-c1 complex subunit Rieske_ mitochondrial OS=Homo sap	182,55	10
67	Q86T23	CROL1_HUMAN Putative ciliary rootlet coiled-coil protein-like 1 protein OS=Homo sap	227,33	10
68	O14529	CUX2_HUMAN Homeobox protein cut-like 2 OS=Homo sapiens OX=9606 GN=CUX2 PE	103,7	10
69	O75843	AP1G2_HUMAN AP-1 complex subunit gamma-like 2 OS=Homo sapiens OX=9606 GN=	200,5	10
70	Q9NXF1	TEX10_HUMAN Testis-expressed protein 10 OS=Homo sapiens OX=9606 GN=TEX10 PE	407,69	10
71	Q15046	SYK_HUMAN Lysine--tRNA ligase OS=Homo sapiens OX=9606 GN=KARS PE=1 SV=3	271,18	10
72	Q6NUP7	PP4R4_HUMAN Serine/threonine-protein phosphatase 4 regulatory subunit 4 OS=Hor	245,06	10
73	P20749	BCL3_HUMAN B-cell lymphoma 3 protein OS=Homo sapiens OX=9606 GN=BCL3 PE=1	274,72	10
74	Q15393	SF3B3_HUMAN Splicing factor 3B subunit 3 OS=Homo sapiens OX=9606 GN=SF3B3 PE	250,5	10
75	Q92499	DDX1_HUMAN ATP-dependent RNA helicase DDX1 OS=Homo sapiens OX=9606 GN=D	396,55	10
76	Q9BYN0	SRXN1_HUMAN Sulfiredoxin-1 OS=Homo sapiens OX=9606 GN=SRXN1 PE=1 SV=2	327,84	10
77	Q8N4C6	NIN_HUMAN Ninein OS=Homo sapiens OX=9606 GN=NIN PE=1 SV=4	326,61	10
78	Q16478	GRIK5_HUMAN Glutamate receptor ionotropic_ kainate 5 OS=Homo sapiens OX=9606	223,72	10
79	Q9HBM1	SPC25_HUMAN Kinetochore protein Spc25 OS=Homo sapiens OX=9606 GN=SPC25 PE	227,46	10
80	Q9Y376	CAB39_HUMAN Calcium-binding protein 39 OS=Homo sapiens OX=9606 GN=CAB39 PE	199,18	10
81	Q9UNZ2	NSF1C_HUMAN NSFL1 cofactor p47 OS=Homo sapiens OX=9606 GN=NSFL1C PE=1 SV=	313,01	10

82	Q9H2M9	RBGPR_HUMAN Rab3 GTPase-activating protein non-catalytic subunit OS=Homo sapie	339,31	10
83	L0R819	ASURF_HUMAN ASNSD1 upstream open reading frame protein OS=Homo sapiens OX=	195,25	10
84	Q2WGJ9	FR1L6_HUMAN Fer-1-like protein 6 OS=Homo sapiens OX=9606 GN=FER1L6 PE=2 SV=:	245,97	10
85	Q6P4F1	FUT10_HUMAN Alpha-(1_3)-fucosyltransferase 10 OS=Homo sapiens OX=9606 GN=FU	208,74	10
86	Q9NR63	CP26B_HUMAN Cytochrome P450 26B1 OS=Homo sapiens OX=9606 GN=CYP26B1 PE=	297,32	10
87	Q96QC0	PP1RA_HUMAN Serine/threonine-protein phosphatase 1 regulatory subunit 10 OS=Hc	398,38	10
88	Q9Y2T3	GUAD_HUMAN Guanine deaminase OS=Homo sapiens OX=9606 GN=GDA PE=1 SV=1	233,39	10
89	Q15006	EMC2_HUMAN ER membrane protein complex subunit 2 OS=Homo sapiens OX=9606	180,68	10
90	Q96AC1	FERM2_HUMAN Fermitin family homolog 2 OS=Homo sapiens OX=9606 GN=FERMT2 I	319,23	10
91	Q8TCJ2	STT3B_HUMAN Dolichyl-diphosphooligosaccharide--protein glycosyltransferase subun	171,01	10
92	Q8WVE0	EFMT1_HUMAN EEF1A lysine methyltransferase 1 OS=Homo sapiens OX=9606 GN=EE	400,44	10
93	Q8IV20	LACC1_HUMAN Laccase domain-containing protein 1 OS=Homo sapiens OX=9606 GN=	265,99	10
94	Q68D10	SPT2_HUMAN Protein SPT2 homolog OS=Homo sapiens OX=9606 GN=SPTY2D1 PE=1 S	108,19	10
95	Q13177	PAK2_HUMAN Serine/threonine-protein kinase PAK 2 OS=Homo sapiens OX=9606 GN	211,6	10
96	Q9Y333	LSM2_HUMAN U6 snRNA-associated Sm-like protein LSm2 OS=Homo sapiens OX=960(	393,67	10
97	Q9Y5X1	SNX9_HUMAN Sorting nexin-9 OS=Homo sapiens OX=9606 GN=SNX9 PE=1 SV=1	200,12	10
98	Q6TDU7	CASC1_HUMAN Protein CASC1 OS=Homo sapiens OX=9606 GN=CASC1 PE=2 SV=2	255,63	10
99	A0A0C4DH25	KVD20_HUMAN Immunoglobulin kappa variable 3D-20 OS=Homo sapiens OX=9606 GI	285,94	10
100	P10619	PPGB_HUMAN Lysosomal protective protein OS=Homo sapiens OX=9606 GN=CTSA PE	246,35	10
101	Q13155	AIMP2_HUMAN Aminoacyl tRNA synthase complex-interacting multifunctional protei	295,22	10
102	Q9Y314	NOSIP_HUMAN Nitric oxide synthase-interacting protein OS=Homo sapiens OX=9606 (	387,12	10
103	Q9Y5V0	ZN706_HUMAN Zinc finger protein 706 OS=Homo sapiens OX=9606 GN=ZNF706 PE=1	329,1	10
104	Q8IXT1	DDIAS_HUMAN DNA damage-induced apoptosis suppressor protein OS=Homo sapien:	266,83	10
105	O95373	IPO7_HUMAN Importin-7 OS=Homo sapiens OX=9606 GN=IPO7 PE=1 SV=1	271,35	10
106	Q8N163	CCAR2_HUMAN Cell cycle and apoptosis regulator protein 2 OS=Homo sapiens OX=96	457,73	10
107	Q13148	TADBP_HUMAN TAR DNA-binding protein 43 OS=Homo sapiens OX=9606 GN=TARDBF	239,13	10
108	Q16401	PSMD5_HUMAN 26S proteasome non-ATPase regulatory subunit 5 OS=Homo sapiens	185,46	10
109	Q86VM9	ZCH18_HUMAN Zinc finger CCCH domain-containing protein 18 OS=Homo sapiens OX:	103,91	10
110	Q9H930	SP14L_HUMAN Nuclear body protein SP140-like protein OS=Homo sapiens OX=9606 G	126,98	10
111	P01619	KV320_HUMAN Immunoglobulin kappa variable 3-20 OS=Homo sapiens OX=9606 GN=	285,94	10
112	P28838	AMPL_HUMAN Cytosol aminopeptidase OS=Homo sapiens OX=9606 GN=LAP3 PE=1 SV	300,15	10

113	P05089	ARGI1_HUMAN Arginase-1 OS=Homo sapiens OX=9606 GN=ARG1 PE=1 SV=2	271,83	10
114	O60241	AGRB2_HUMAN Adhesion G protein-coupled receptor B2 OS=Homo sapiens OX=9606	190,02	10
115	Q96DB5	RMD1_HUMAN Regulator of microtubule dynamics protein 1 OS=Homo sapiens OX=9(	201,08	10
116	P26640	SYVC_HUMAN Valine--tRNA ligase OS=Homo sapiens OX=9606 GN=VARS PE=1 SV=4	198,02	10
117	P34896	GLYC_HUMAN Serine hydroxymethyltransferase_ cytosolic OS=Homo sapiens OX=960	520,75	10
118	P42785	PCP_HUMAN Lysosomal Pro-X carboxypeptidase OS=Homo sapiens OX=9606 GN=PRC	278,41	10
119	P05067	A4_HUMAN Amyloid-beta A4 protein OS=Homo sapiens OX=9606 GN=APP PE=1 SV=3	240,79	10
120	Q13103	SPP24_HUMAN Secreted phosphoprotein 24 OS=Homo sapiens OX=9606 GN=SPP2 PE	308,93	10
121	Q9Y624	JAM1_HUMAN Junctional adhesion molecule A OS=Homo sapiens OX=9606 GN=F11R	250,63	10
122	Q99471	PFD5_HUMAN Prefoldin subunit 5 OS=Homo sapiens OX=9606 GN=PFDN5 PE=1 SV=2	181,56	10
123	Q15637	SF01_HUMAN Splicing factor 1 OS=Homo sapiens OX=9606 GN=SF1 PE=1 SV=4	304,77	10
124	Q7Z4V5	HDGR2_HUMAN Hepatoma-derived growth factor-related protein 2 OS=Homo sapiens	207,32	10
125	P23368	MAOM_HUMAN NAD-dependent malic enzyme_ mitochondrial OS=Homo sapiens OX	147,65	10
126	Q9Y617	SERC_HUMAN Phosphoserine aminotransferase OS=Homo sapiens OX=9606 GN=PSAT	508,14	10
127	P06493	CDK1_HUMAN Cyclin-dependent kinase 1 OS=Homo sapiens OX=9606 GN=CDK1 PE=1	272,87	10
128	Q15629	TRAM1_HUMAN Translocating chain-associated membrane protein 1 OS=Homo sapier	191,5	10
129	Q96Q89	KI20B_HUMAN Kinesin-like protein KIF20B OS=Homo sapiens OX=9606 GN=KIF20B PE	172,46	10
130	P06126	CD1A_HUMAN T-cell surface glycoprotein CD1a OS=Homo sapiens OX=9606 GN=CD1A	284,56	10
131	O60551	NMT2_HUMAN Glycylpeptide N-tetradecanoyltransferase 2 OS=Homo sapiens OX=96	200,74	10
132	P07203	GPX1_HUMAN Glutathione peroxidase 1 OS=Homo sapiens OX=9606 GN=GPX1 PE=1 S	419,98	10
133	Q7Z4T9	CFA91_HUMAN Cilia- and flagella-associated protein 91 OS=Homo sapiens OX=9606 G	180,07	10
134	Q7Z4T8	GLTL5_HUMAN Inactive polypeptide N-acetylgalactosaminyltransferase-like protein 5	449,95	10
135	Q9UDY6	TRI10_HUMAN Tripartite motif-containing protein 10 OS=Homo sapiens OX=9606 GN=	283,74	10
136	Q6UXP7	F151B_HUMAN Protein FAM151B OS=Homo sapiens OX=9606 GN=FAM151B PE=1 SV=	170,72	10
137	Q99442	SEC62_HUMAN Translocation protein SEC62 OS=Homo sapiens OX=9606 GN=SEC62 PI	300,99	10
138	Q9UNL2	SSRG_HUMAN Translocon-associated protein subunit gamma OS=Homo sapiens OX=9	208,07	10
139	P05026	AT1B1_HUMAN Sodium/potassium-transporting ATPase subunit beta-1 OS=Homo sap	639,78	10
140	Q7Z4S6	KI21A_HUMAN Kinesin-like protein KIF21A OS=Homo sapiens OX=9606 GN=KIF21A PE	125,23	10
141	Q99439	CNN2_HUMAN Calponin-2 OS=Homo sapiens OX=9606 GN=CNN2 PE=1 SV=4	247,05	10
142	O00303	EIF3F_HUMAN Eukaryotic translation initiation factor 3 subunit F OS=Homo sapiens O	314,1	10
143	Q9Y2G7	ZFP30_HUMAN Zinc finger protein 30 homolog OS=Homo sapiens OX=9606 GN=ZFP30	431,2	10

144	Q99798	ACON_HUMAN Aconitate hydratase_ mitochondrial OS=Homo sapiens OX=9606 GN=A	261,04	10
145	Q99797	MIPEP_HUMAN Mitochondrial intermediate peptidase OS=Homo sapiens OX=9606 GN	341,72	10
146	Q96A65	EXOC4_HUMAN Exocyst complex component 4 OS=Homo sapiens OX=9606 GN=EXOC	316,34	10
147	O75153	CLU_HUMAN Clustered mitochondria protein homolog OS=Homo sapiens OX=9606 GI	218,07	10
148	Q13418	ILK_HUMAN Integrin-linked protein kinase OS=Homo sapiens OX=9606 GN=ILK PE=1 S	336,85	10
149	Q6UXN7	TO20L_HUMAN TOMM20-like protein 1 OS=Homo sapiens OX=9606 GN=TOMM20L PI	377,65	10
150	Q9Y5L4	TIM13_HUMAN Mitochondrial import inner membrane translocase subunit Tim13 OS	861,39	10
151	O60888	CUTA_HUMAN Protein CutA OS=Homo sapiens OX=9606 GN=CUTA PE=1 SV=2	230,16	10
152	O95996	APCL_HUMAN Adenomatous polyposis coli protein 2 OS=Homo sapiens OX=9606 GN=	111,13	10
153	Q13409	DC1I2_HUMAN Cytoplasmic dynein 1 intermediate chain 2 OS=Homo sapiens OX=960	258,8	10
154	Q13404	UB2V1_HUMAN Ubiquitin-conjugating enzyme E2 variant 1 OS=Homo sapiens OX=960	499,74	10
155	O15264	MK13_HUMAN Mitogen-activated protein kinase 13 OS=Homo sapiens OX=9606 GN=I	189,74	10
156	O95983	MBD3_HUMAN Methyl-CpG-binding domain protein 3 OS=Homo sapiens OX=9606 GN	197,97	10
157	Q32MZ4	LRRF1_HUMAN Leucine-rich repeat flightless-interacting protein 1 OS=Homo sapiens (	184,16	10
158	Q8TBZ2	MYBPP_HUMAN MYCBP-associated protein OS=Homo sapiens OX=9606 GN=MYCBPAI	493,38	10
159	P14324	FPPS_HUMAN Farnesyl pyrophosphate synthase OS=Homo sapiens OX=9606 GN=FDPS	228,29	10
160	O15258	RER1_HUMAN Protein RER1 OS=Homo sapiens OX=9606 GN=RER1 PE=1 SV=1	246,48	10
161	O60503	ADCY9_HUMAN Adenylate cyclase type 9 OS=Homo sapiens OX=9606 GN=ADCY9 PE=	192,99	10
162	P0DN82	O12D1_HUMAN Olfactory receptor 12D1 OS=Homo sapiens OX=9606 GN=OR12D1 PE	239,74	10
163	O75489	NDUS3_HUMAN NADH dehydrogenase [ubiquinone] iron-sulfur protein 3_ mitochond	272,46	10
164	Q8TBY8	PMFBP_HUMAN Polyamine-modulated factor 1-binding protein 1 OS=Homo sapiens C	294,81	10
165	O00625	PIR_HUMAN Pirin OS=Homo sapiens OX=9606 GN=PIR PE=1 SV=1	454,15	10
166	P0DN76	U2AF5_HUMAN Splicing factor U2AF 35 kDa subunit-like protein OS=Homo sapiens OX	178,57	10
167	P55145	MANF_HUMAN Mesencephalic astrocyte-derived neurotrophic factor OS=Homo sapie	292,41	10
168	Q9BXY0	MAK16_HUMAN Protein MAK16 homolog OS=Homo sapiens OX=9606 GN=MAK16 PE	235,49	10
169	O75477	ERLN1_HUMAN Erlin-1 OS=Homo sapiens OX=9606 GN=ERLIN1 PE=1 SV=1	282,74	10
170	Q8N752	KC1AL_HUMAN Casein kinase I isoform alpha-like OS=Homo sapiens OX=9606 GN=CSN	230,86	10
171	Q9Y2B0	CNPY2_HUMAN Protein canopy homolog 2 OS=Homo sapiens OX=9606 GN=CNPY2 PE	406,54	10
172	Q04759	KPCT_HUMAN Protein kinase C theta type OS=Homo sapiens OX=9606 GN=PRKCQ PE	331,59	10
173	O43615	TIM44_HUMAN Mitochondrial import inner membrane translocase subunit TIM44 OS	224,78	10
174	P63027	VAMP2_HUMAN Vesicle-associated membrane protein 2 OS=Homo sapiens OX=9606	777,06	10

175	Q9Y2A7	NCKP1_HUMAN Nck-associated protein 1 OS=Homo sapiens OX=9606 GN=NCKAP1 PE	252,05	10
176	P48681	NEST_HUMAN Nestin OS=Homo sapiens OX=9606 GN=NES PE=1 SV=2	402,81	10
177	Q8WXX5	DNJC9_HUMAN DnaJ homolog subfamily C member 9 OS=Homo sapiens OX=9606 GN	314,19	10
178	B2RXH8	HNRC2_HUMAN Heterogeneous nuclear ribonucleoprotein C-like 2 OS=Homo sapiens	309,22	10
179	O15212	PFD6_HUMAN Prefoldin subunit 6 OS=Homo sapiens OX=9606 GN=PFDN6 PE=1 SV=1	276,29	10
180	Q16099	GRIK4_HUMAN Glutamate receptor ionotropic_ kainate 4 OS=Homo sapiens OX=9606	251,13	10
181	O60812	HNRC1_HUMAN Heterogeneous nuclear ribonucleoprotein C-like 1 OS=Homo sapiens	305,03	10
182	P0DMR1	HNRC4_HUMAN Heterogeneous nuclear ribonucleoprotein C-like 4 OS=Homo sapiens	305,03	10
183	P56545	CTBP2_HUMAN C-terminal-binding protein 2 OS=Homo sapiens OX=9606 GN=CTBP2 F	264,74	10
184	P06730	IF4E_HUMAN Eukaryotic translation initiation factor 4E OS=Homo sapiens OX=9606 GI	487,08	10
185	P07814	SYEP_HUMAN Bifunctional glutamate/proline--tRNA ligase OS=Homo sapiens OX=960(	303,7	10
186	P62256	UBE2H_HUMAN Ubiquitin-conjugating enzyme E2 H OS=Homo sapiens OX=9606 GN=U	500,39	10
187	Q96T21	SEBP2_HUMAN Selenocysteine insertion sequence-binding protein 2 OS=Homo sapier	106,26	10
188	P06727	APOA4_HUMAN Apolipoprotein A-IV OS=Homo sapiens OX=9606 GN=APOA4 PE=1 SV	194,38	10
189	P19174	PLCG1_HUMAN 1-phosphatidylinositol 4_5-bisphosphate phosphodiesterase gamma-	278,21	10
190	Q9BXS5	AP1M1_HUMAN AP-1 complex subunit mu-1 OS=Homo sapiens OX=9606 GN=AP1M1	185,86	10
191	P29590	PML_HUMAN Protein PML OS=Homo sapiens OX=9606 GN=PML PE=1 SV=3	375,8	10
192	A3KMH1	VWA8_HUMAN von Willebrand factor A domain-containing protein 8 OS=Homo sapie	228,23	10
193	Q8N9S9	SNX31_HUMAN Sorting nexin-31 OS=Homo sapiens OX=9606 GN=SNX31 PE=1 SV=3	198,93	10
194	Q00341	VIGLN_HUMAN Vigilin OS=Homo sapiens OX=9606 GN=HDLBP PE=1 SV=2	188,48	10
195	P20674	COX5A_HUMAN Cytochrome c oxidase subunit 5A_ mitochondrial OS=Homo sapiens (	366,05	10
196	Q9H227	GBA3_HUMAN Cytosolic beta-glucosidase OS=Homo sapiens OX=9606 GN=GBA3 PE=1	234,54	10
197	Q96SN8	CK5P2_HUMAN CDK5 regulatory subunit-associated protein 2 OS=Homo sapiens OX=9	279,87	10
198	P28482	MK01_HUMAN Mitogen-activated protein kinase 1 OS=Homo sapiens OX=9606 GN=M	598,7	10
199	Q9P035	HACD3_HUMAN Very-long-chain (3R)-3-hydroxyacyl-CoA dehydratase 3 OS=Homo sap	243,19	10
200	Q12929	EPS8_HUMAN Epidermal growth factor receptor kinase substrate 8 OS=Homo sapiens	305,15	10
201	Q9BUJ2	HNRL1_HUMAN Heterogeneous nuclear ribonucleoprotein U-like protein 1 OS=Homo	277,28	10
202	Q86V81	THOC4_HUMAN THO complex subunit 4 OS=Homo sapiens OX=9606 GN=ALYREF PE=1	165,6	10
203	Q9HB07	MYG1_HUMAN UPF0160 protein MYG1_ mitochondrial OS=Homo sapiens OX=9606 G	276,04	10
204	P22830	HEMH_HUMAN Ferrochelatase_ mitochondrial OS=Homo sapiens OX=9606 GN=FECH	284,55	10
205	Q3ZCX4	ZN568_HUMAN Zinc finger protein 568 OS=Homo sapiens OX=9606 GN=ZNF568 PE=2	231,64	10

206	P30711	GSTT1_HUMAN Glutathione S-transferase theta-1 OS=Homo sapiens OX=9606 GN=GS	268,32	10
207	P20648	ATP4A_HUMAN Potassium-transporting ATPase alpha chain 1 OS=Homo sapiens OX=9	205,9	10
208	P14927	QCR7_HUMAN Cytochrome b-c1 complex subunit 7 OS=Homo sapiens OX=9606 GN=U	465,48	10
209	Q12907	LMAN2_HUMAN Vesicular integral-membrane protein VIP36 OS=Homo sapiens OX=96	270,05	10
210	Q12906	ILF3_HUMAN Interleukin enhancer-binding factor 3 OS=Homo sapiens OX=9606 GN=IL	236,4	10
211	Q12905	ILF2_HUMAN Interleukin enhancer-binding factor 2 OS=Homo sapiens OX=9606 GN=IL	288,73	10
212	A6NC57	ANR62_HUMAN Ankyrin repeat domain-containing protein 62 OS=Homo sapiens OX=9	314,25	10
213	Q9BRA2	TXD17_HUMAN Thioredoxin domain-containing protein 17 OS=Homo sapiens OX=960	461,74	10
214	Q96G28	CFA36_HUMAN Cilia- and flagella-associated protein 36 OS=Homo sapiens OX=9606 G	419,44	10
215	Q969X0	RIPL2_HUMAN RILP-like protein 2 OS=Homo sapiens OX=9606 GN=RILPL2 PE=1 SV=1	269,02	10
216	Q86XZ4	SPAS2_HUMAN Spermatogenesis-associated serine-rich protein 2 OS=Homo sapiens C	148,73	10
217	P01225	FSHB_HUMAN Follitropin subunit beta OS=Homo sapiens OX=9606 GN=FSHB PE=1 SV=	171,52	10
218	P61457	PHS_HUMAN Pterin-4-alpha-carbinolamine dehydratase OS=Homo sapiens OX=9606 G	205,69	10
219	Q9BXL7	CAR11_HUMAN Caspase recruitment domain-containing protein 11 OS=Homo sapiens	148,67	10
220	P35244	RFA3_HUMAN Replication protein A 14 kDa subunit OS=Homo sapiens OX=9606 GN=F	1086,01	10
221	Q76KD6	SPERI_HUMAN Speriolin OS=Homo sapiens OX=9606 GN=SPATC1 PE=2 SV=2	181,01	10
222	Q13098	CSN1_HUMAN COP9 signalosome complex subunit 1 OS=Homo sapiens OX=9606 GN=	243,32	10
223	P62891	RL39_HUMAN 60S ribosomal protein L39 OS=Homo sapiens OX=9606 GN=RPL39 PE=1	1654,71	10
224	Q9UMX0	UBQL1_HUMAN Ubiquilin-1 OS=Homo sapiens OX=9606 GN=UBQLN1 PE=1 SV=2	283,3	10
225	Q9H4Q3	PRD13_HUMAN PR domain zinc finger protein 13 OS=Homo sapiens OX=9606 GN=PRD	260,39	10
226	Q96IU4	ABHEB_HUMAN Protein ABHD14B OS=Homo sapiens OX=9606 GN=ABHD14B PE=1 SV	274,14	10
227	O14732	IMPA2_HUMAN Inositol monophosphatase 2 OS=Homo sapiens OX=9606 GN=IMPA2	316,22	10
228	Q9UJQ7	SCP2D_HUMAN SCP2 sterol-binding domain-containing protein 1 OS=Homo sapiens O	367,51	10
229	Q9UN86	G3BP2_HUMAN Ras GTPase-activating protein-binding protein 2 OS=Homo sapiens OX	249,62	10
230	Q14165	MLEC_HUMAN Malectin OS=Homo sapiens OX=9606 GN=MLEC PE=1 SV=1	172,37	10
231	Q8N6D5	ANR29_HUMAN Ankyrin repeat domain-containing protein 29 OS=Homo sapiens OX=9	273,57	10
232	Q9UGI8	TES_HUMAN Testin OS=Homo sapiens OX=9606 GN=TES PE=1 SV=1	186,83	10
233	Q8WXI9	P66B_HUMAN Transcriptional repressor p66-beta OS=Homo sapiens OX=9606 GN=GA	298,48	10
234	Q9P2P5	HECW2_HUMAN E3 ubiquitin-protein ligase HECW2 OS=Homo sapiens OX=9606 GN=H	136,87	10
235	P19784	CSK22_HUMAN Casein kinase II subunit alpha' OS=Homo sapiens OX=9606 GN=CSNK2	203,69	10
236	Q92698	RAD54_HUMAN DNA repair and recombination protein RAD54-like OS=Homo sapiens	156,04	10

237	O96008	TOM40_HUMAN Mitochondrial import receptor subunit TOM40 homolog OS=Homo s	253,7	10
238	Q9H4M9	EHD1_HUMAN EH domain-containing protein 1 OS=Homo sapiens OX=9606 GN=EHD1	194,97	10
239	P52788	SPSY_HUMAN Spermine synthase OS=Homo sapiens OX=9606 GN=SMS PE=1 SV=2	565,94	10
240	Q14498	RBM39_HUMAN RNA-binding protein 39 OS=Homo sapiens OX=9606 GN=RBM39 PE=	236,35	10
241	O95273	CCDB1_HUMAN Cyclin-D1-binding protein 1 OS=Homo sapiens OX=9606 GN=CCNDBP	164,15	10
242	Q14494	NF2L1_HUMAN Endoplasmic reticulum membrane sensor NFE2L1 OS=Homo sapiens (	278,31	10
243	Q8TBF8	FA81A_HUMAN Protein FAM81A OS=Homo sapiens OX=9606 GN=FAM81A PE=2 SV=3	308,28	10
244	Q8TEL6	TP4AP_HUMAN Short transient receptor potential channel 4-associated protein OS=H	255,71	10
245	Q13045	FLII_HUMAN Protein flightless-1 homolog OS=Homo sapiens OX=9606 GN=FLII PE=1 S\	149,95	10
246	A6NHY2	AKD1B_HUMAN Ankyrin repeat and death domain-containing protein 1B OS=Homo sa	262,49	10
247	Q9UMS4	PRP19_HUMAN Pre-mRNA-processing factor 19 OS=Homo sapiens OX=9606 GN=PRPF	216,69	10
248	P36639	8ODP_HUMAN 7_8-dihydro-8-oxoguanine triphosphatase OS=Homo sapiens OX=9606	390,82	10
249	P45973	CBX5_HUMAN Chromobox protein homolog 5 OS=Homo sapiens OX=9606 GN=CBX5 F	188,49	10
250	Q9H4L7	SMRCD_HUMAN SWI/SNF-related matrix-associated actin-dependent regulator of chr	104,64	10
251	P19404	NDUV2_HUMAN NADH dehydrogenase [ubiquinone] flavoprotein 2_ mitochondrial O:	267,16	10
252	Q96FJ2	DYL2_HUMAN Dynein light chain 2_ cytoplasmic OS=Homo sapiens OX=9606 GN=DYN	546,48	10
253	Q96IP4	FA46A_HUMAN Putative nucleotidyltransferase FAM46A OS=Homo sapiens OX=9606	234,06	10
254	Q16653	MOG_HUMAN Myelin-oligodendrocyte glycoprotein OS=Homo sapiens OX=9606 GN=I	250,89	10
255	Q9UMR2	DD19B_HUMAN ATP-dependent RNA helicase DDX19B OS=Homo sapiens OX=9606 GI	158,83	10
256	Q9C035	TRIM5_HUMAN Tripartite motif-containing protein 5 OS=Homo sapiens OX=9606 GN=	201,47	10
257	Q5VTR2	BRE1A_HUMAN E3 ubiquitin-protein ligase BRE1A OS=Homo sapiens OX=9606 GN=RN	363,06	10
258	Q86Y38	XYLT1_HUMAN Xylosyltransferase 1 OS=Homo sapiens OX=9606 GN=XYLT1 PE=1 SV=1	411,95	10
259	Q15560	TCEA2_HUMAN Transcription elongation factor A protein 2 OS=Homo sapiens OX=960	279,64	10
260	Q14116	IL18_HUMAN Interleukin-18 OS=Homo sapiens OX=9606 GN=IL18 PE=1 SV=1	331,57	10
261	P41252	SYIC_HUMAN Isoleucine--tRNA ligase_ cytoplasmic OS=Homo sapiens OX=9606 GN=IA	224,36	10
262	P42338	PK3CB_HUMAN Phosphatidylinositol 4_5-bisphosphate 3-kinase catalytic subunit beta	177,03	10
263	P08237	PFKAM_HUMAN ATP-dependent 6-phosphofructokinase_ muscle type OS=Homo sapi	395,64	10
264	Q96CB9	NSUN4_HUMAN 5-methylcytosine rRNA methyltransferase NSUN4 OS=Homo sapiens	307,29	10
265	Q14108	SCRB2_HUMAN Lysosome membrane protein 2 OS=Homo sapiens OX=9606 GN=SCAR	205,64	10
266	P08590	MYL3_HUMAN Myosin light chain 3 OS=Homo sapiens OX=9606 GN=MYL3 PE=1 SV=3	512,55	10
267	Q06210	GFPT1_HUMAN Glutamine--fructose-6-phosphate aminotransferase [isomerizing] 1 O	189,22	10

268	Q9UPV0	CE164_HUMAN Centrosomal protein of 164 kDa OS=Homo sapiens OX=9606 GN=CEP1	148,93	10
269	Q8IZV5	RDH10_HUMAN Retinol dehydrogenase 10 OS=Homo sapiens OX=9606 GN=RDH10 PE	230,8	10
270	O95232	LC7L3_HUMAN Luc7-like protein 3 OS=Homo sapiens OX=9606 GN=LUC7L3 PE=1 SV=2	269,33	10
271	Q16629	SRSF7_HUMAN Serine/arginine-rich splicing factor 7 OS=Homo sapiens OX=9606 GN=S	352,41	10
272	P09669	COX6C_HUMAN Cytochrome c oxidase subunit 6C OS=Homo sapiens OX=9606 GN=CO	703,1	10
273	Q13363	CTBP1_HUMAN C-terminal-binding protein 1 OS=Homo sapiens OX=9606 GN=CTBP1 F	273,82	10
274	P08579	RU2B_HUMAN U2 small nuclear ribonucleoprotein B'' OS=Homo sapiens OX=9606 GN	208,78	10
275	P11908	PRPS2_HUMAN Ribose-phosphate pyrophosphokinase 2 OS=Homo sapiens OX=9606 G	666,86	10
276	Q14444	CAPR1_HUMAN Caprin-1 OS=Homo sapiens OX=9606 GN=CAPRIN1 PE=1 SV=2	225,85	10
277	Q6ZS92	YD022_HUMAN Putative uncharacterized protein FLJ45721 OS=Homo sapiens OX=960	237,28	10
278	O43237	DC1L2_HUMAN Cytoplasmic dynein 1 light intermediate chain 2 OS=Homo sapiens OX	260,18	10
279	Q5VX52	SPAT1_HUMAN Spermatogenesis-associated protein 1 OS=Homo sapiens OX=9606 GN	174,08	10
280	P50552	VASP_HUMAN Vasodilator-stimulated phosphoprotein OS=Homo sapiens OX=9606 GN	256,96	10
281	Q92628	K0232_HUMAN Uncharacterized protein KIAA0232 OS=Homo sapiens OX=9606 GN=KI	283,96	10
282	Q9NPJ3	ACO13_HUMAN Acyl-coenzyme A thioesterase 13 OS=Homo sapiens OX=9606 GN=AC	429,22	10
283	P40126	TYRP2_HUMAN L-dopachrome tautomerase OS=Homo sapiens OX=9606 GN=DCT PE=	143,84	10
284	O00584	RNT2_HUMAN Ribonuclease T2 OS=Homo sapiens OX=9606 GN=RNASET2 PE=1 SV=2	214,14	10
285	Q13347	EIF3I_HUMAN Eukaryotic translation initiation factor 3 subunit I OS=Homo sapiens OX	158,46	10
286	P41567	EIF1_HUMAN Eukaryotic translation initiation factor 1 OS=Homo sapiens OX=9606 GN	550,99	10
287	O00217	NDUS8_HUMAN NADH dehydrogenase [ubiquinone] iron-sulfur protein 8_ mitochond	405,98	10
288	Q92973	TNPO1_HUMAN Transportin-1 OS=Homo sapiens OX=9606 GN=TNPO1 PE=1 SV=2	222,28	10
289	Q9UPR0	PLCL2_HUMAN Inactive phospholipase C-like protein 2 OS=Homo sapiens OX=9606 GN	249,52	10
290	Q9H4E7	DEFI6_HUMAN Differentially expressed in FDCP 6 homolog OS=Homo sapiens OX=960	410,42	10
291	Q86XL3	ANKL2_HUMAN Ankyrin repeat and LEM domain-containing protein 2 OS=Homo sapie	249,9	10
292	P30042	ES1_HUMAN ES1 protein homolog_ mitochondrial OS=Homo sapiens OX=9606 GN=C2	194,16	10
293	O00560	SDCB1_HUMAN Syntenin-1 OS=Homo sapiens OX=9606 GN=SDCBP PE=1 SV=1	623,67	10
294	O60437	PEPL_HUMAN Periplakin OS=Homo sapiens OX=9606 GN=PPL PE=1 SV=4	105,68	10
295	P51970	NDUA8_HUMAN NADH dehydrogenase [ubiquinone] 1 alpha subcomplex subunit 8 OS	281,51	10
296	Q01081	U2AF1_HUMAN Splicing factor U2AF 35 kDa subunit OS=Homo sapiens OX=9606 GN=U	178,57	10
297	Q86UE4	LYRIC_HUMAN Protein LYRIC OS=Homo sapiens OX=9606 GN=MTDH PE=1 SV=2	176,28	10
298	Q9P2E3	ZNFX1_HUMAN NFX1-type zinc finger-containing protein 1 OS=Homo sapiens OX=960	281,25	10

299	Q8N684	CPSF7_HUMAN Cleavage and polyadenylation specificity factor subunit 7 OS=Homo sa	206,06	10
300	Q8WU76	SCFD2_HUMAN Sec1 family domain-containing protein 2 OS=Homo sapiens OX=9606	281,03	10
301	Q969G5	CAVN3_HUMAN Caveolae-associated protein 3 OS=Homo sapiens OX=9606 GN=CAVIN	220,43	10
302	Q15836	VAMP3_HUMAN Vesicle-associated membrane protein 3 OS=Homo sapiens OX=9606	982,68	10
303	O15160	RPAC1_HUMAN DNA-directed RNA polymerases I and III subunit RPAC1 OS=Homo sap	259,34	10
304	Q31612	1B73_HUMAN HLA class I histocompatibility antigen_ B-73 alpha chain OS=Homo sapi	338,79	10
305	Q8WU68	U2AF4_HUMAN Splicing factor U2AF 26 kDa subunit OS=Homo sapiens OX=9606 GN=U	178,57	10
306	Q96BX8	MOB3A_HUMAN MOB kinase activator 3A OS=Homo sapiens OX=9606 GN=MOB3A PE	255,13	10
307	O60762	DPM1_HUMAN Dolichol-phosphate mannosyltransferase subunit 1 OS=Homo sapiens	260,97	10
308	Q9BTT0	AN32E_HUMAN Acidic leucine-rich nuclear phosphoprotein 32 family member E OS=H	223,31	10
309	O15151	MDM4_HUMAN Protein Mdm4 OS=Homo sapiens OX=9606 GN=MDM4 PE=1 SV=2	266,25	10
310	O94782	UBP1_HUMAN Ubiquitin carboxyl-terminal hydrolase 1 OS=Homo sapiens OX=9606 GI	299,58	10
311	Q15819	UB2V2_HUMAN Ubiquitin-conjugating enzyme E2 variant 2 OS=Homo sapiens OX=960	363,95	10
312	O15146	MUSK_HUMAN Muscle_ skeletal receptor tyrosine-protein kinase OS=Homo sapiens C	229,29	10
313	Q92917	GPKOW_HUMAN G-patch domain and KOW motifs-containing protein OS=Homo sapie	179,03	10
314	P46060	RAGP1_HUMAN Ran GTPase-activating protein 1 OS=Homo sapiens OX=9606 GN=RAN	292,72	10
315	P14209	CD99_HUMAN CD99 antigen OS=Homo sapiens OX=9606 GN=CD99 PE=1 SV=1	312,6	10
316	Q13630	FCL_HUMAN GDP-L-fucose synthase OS=Homo sapiens OX=9606 GN=TSTA3 PE=1 SV=	266,49	10
317	B7ZW38	HNRC3_HUMAN Heterogeneous nuclear ribonucleoprotein C-like 3 OS=Homo sapiens	305,03	10
318	Q9NZ56	FMN2_HUMAN Formin-2 OS=Homo sapiens OX=9606 GN=FMN2 PE=1 SV=4	193,01	10
319	O00505	IMA4_HUMAN Importin subunit alpha-4 OS=Homo sapiens OX=9606 GN=KPNA3 PE=1	269,47	10
320	O60739	EIF1B_HUMAN Eukaryotic translation initiation factor 1b OS=Homo sapiens OX=9606 (	550,99	10
321	O15126	SCAM1_HUMAN Secretory carrier-associated membrane protein 1 OS=Homo sapiens	331,6	10
322	P38159	RBMX_HUMAN RNA-binding motif protein_ X chromosome OS=Homo sapiens OX=960	299,36	10
323	P07741	APT_HUMAN Adenine phosphoribosyltransferase OS=Homo sapiens OX=9606 GN=API	335,03	10
324	Q8TAV0	FA76A_HUMAN Protein FAM76A OS=Homo sapiens OX=9606 GN=FAM76A PE=2 SV=1	251,55	10
325	Q04637	IF4G1_HUMAN Eukaryotic translation initiation factor 4 gamma 1 OS=Homo sapiens C	196,52	10
326	O95837	GNA14_HUMAN Guanine nucleotide-binding protein subunit alpha-14 OS=Homo sapie	211,36	10
327	O95831	AIFM1_HUMAN Apoptosis-inducing factor 1_ mitochondrial OS=Homo sapiens OX=960	189,97	10
328	O75340	PDCD6_HUMAN Programmed cell death protein 6 OS=Homo sapiens OX=9606 GN=PD	547,14	10
329	Q99615	DNJC7_HUMAN DnaJ homolog subfamily C member 7 OS=Homo sapiens OX=9606 GN	200,4	10

330	P28070	PSB4_HUMAN Proteasome subunit beta type-4 OS=Homo sapiens OX=9606 GN=PSME	276,07	10
331	Q6DD88	ATLA3_HUMAN Atlastin-3 OS=Homo sapiens OX=9606 GN=ATL3 PE=1 SV=1	292,95	10
332	P19086	GNAZ_HUMAN Guanine nucleotide-binding protein G(z) subunit alpha OS=Homo sapie	211,36	10
333	O15455	TLR3_HUMAN Toll-like receptor 3 OS=Homo sapiens OX=9606 GN=TLR3 PE=1 SV=1	118,48	10
334	Q00266	METK1_HUMAN S-adenosylmethionine synthase isoform type-1 OS=Homo sapiens OX	203,67	10
335	P07711	CATL1_HUMAN Cathepsin L1 OS=Homo sapiens OX=9606 GN=CTSL PE=1 SV=2	290,87	10
336	Q02790	FKBP4_HUMAN Peptidyl-prolyl cis-trans isomerase FKBP4 OS=Homo sapiens OX=9606	346,61	10
337	P20591	MX1_HUMAN Interferon-induced GTP-binding protein Mx1 OS=Homo sapiens OX=960	282,64	10
338	Q96BQ5	CC127_HUMAN Coiled-coil domain-containing protein 127 OS=Homo sapiens OX=960	180,39	10
339	Q09160	1A80_HUMAN HLA class I histocompatibility antigen_ A-80 alpha chain OS=Homo sapi	411,75	10
340	Q8TB33	CX024_HUMAN Putative uncharacterized protein encoded by LINC01560 OS=Homo sa	182,96	10
341	O75310	UDB11_HUMAN UDP-glucuronosyltransferase 2B11 OS=Homo sapiens OX=9606 GN=U	424,5	10
342	P14866	HNRPL_HUMAN Heterogeneous nuclear ribonucleoprotein L OS=Homo sapiens OX=96	316,16	10
343	Q8IZF7	AGRF2_HUMAN Adhesion G-protein coupled receptor F2 OS=Homo sapiens OX=9606	264,81	10
344	Q9UJ98	STAG3_HUMAN Cohesin subunit SA-3 OS=Homo sapiens OX=9606 GN=STAG3 PE=1 SV	49,79	10
345	Q02413	DSG1_HUMAN Desmoglein-1 OS=Homo sapiens OX=9606 GN=DSG1 PE=1 SV=2	211,69	10
346	Q6FI81	CPIN1_HUMAN Anamorsin OS=Homo sapiens OX=9606 GN=CIAPIN1 PE=1 SV=2	215,78	10
347	Q96C01	F136A_HUMAN Protein FAM136A OS=Homo sapiens OX=9606 GN=FAM136A PE=1 SV	240,4	10
348	Q00597	FANCC_HUMAN Fanconi anemia group C protein OS=Homo sapiens OX=9606 GN=FAN	330,49	10
349	P82979	SARNP_HUMAN SAP domain-containing ribonucleoprotein OS=Homo sapiens OX=960	284,72	10
350	Q5JNZ3	ZN311_HUMAN Zinc finger protein 311 OS=Homo sapiens OX=9606 GN=ZNF311 PE=2	228,28	10
351	Q13907	IDI1_HUMAN Isopentenyl-diphosphate Delta-isomerase 1 OS=Homo sapiens OX=9606	377,65	10
352	P39880	CUX1_HUMAN Homeobox protein cut-like 1 OS=Homo sapiens OX=9606 GN=CUX1 PE	266,03	10
353	Q502W6	VWA3B_HUMAN von Willebrand factor A domain-containing protein 3B OS=Homo sap	414,93	10
354	Q96I99	SUCB2_HUMAN Succinate--CoA ligase [GDP-forming] subunit beta_ mitochondrial OS=	319,18	10
355	Q6A163	K1C39_HUMAN Keratin_ type I cytoskeletal 39 OS=Homo sapiens OX=9606 GN=KRT39	208,8	10
356	Q08380	LG3BP_HUMAN Galectin-3-binding protein OS=Homo sapiens OX=9606 GN=LGALS3BP	251,28	10
357	Q9UFN0	NPS3A_HUMAN Protein NipSnap homolog 3A OS=Homo sapiens OX=9606 GN=NIPSNA	279,7	10
358	P61009	SPCS3_HUMAN Signal peptidase complex subunit 3 OS=Homo sapiens OX=9606 GN=S	231,79	10
359	Q7Z3B3	KANL1_HUMAN KAT8 regulatory NSL complex subunit 1 OS=Homo sapiens OX=9606 G	276,82	10
360	P82932	RT06_HUMAN 28S ribosomal protein S6_ mitochondrial OS=Homo sapiens OX=9606 G	546,94	10

361	P49915	GUAA_HUMAN GMP synthase [glutamine-hydrolyzing] OS=Homo sapiens OX=9606 GN	335,15	10
362	O75608	LYPA1_HUMAN Acyl-protein thioesterase 1 OS=Homo sapiens OX=9606 GN=LYPLA1 PI	295,14	10
363	Q8TDQ7	GNPI2_HUMAN Glucosamine-6-phosphate isomerase 2 OS=Homo sapiens OX=9606 G	156,64	10
364	P10451	OSTP_HUMAN Osteopontin OS=Homo sapiens OX=9606 GN=SPP1 PE=1 SV=1	317,26	10
365	P21964	COMT_HUMAN Catechol O-methyltransferase OS=Homo sapiens OX=9606 GN=COMT	316,44	10
366	P28331	NDUS1_HUMAN NADH-ubiquinone oxidoreductase 75 kDa subunit_ mitochondrial OS	219,91	10
367	Q9BTE7	DCNL5_HUMAN DCN1-like protein 5 OS=Homo sapiens OX=9606 GN=DCUN1D5 PE=1 !	216,88	10
368	Q8IW93	ARHGJ_HUMAN Rho guanine nucleotide exchange factor 19 OS=Homo sapiens OX=96	256,2	10
369	P59090	TSAS2_HUMAN Putative uncharacterized protein TSPEAR-AS2 OS=Homo sapiens OX=9	317,51	10
370	Q15155	NOMO1_HUMAN Nodal modulator 1 OS=Homo sapiens OX=9606 GN=NOMO1 PE=1 S'	255,57	10
371	Q8IVW1	ARL17_HUMAN ADP-ribosylation factor-like protein 17 OS=Homo sapiens OX=9606 GN	425,78	10
372	Q00526	CDK3_HUMAN Cyclin-dependent kinase 3 OS=Homo sapiens OX=9606 GN=CDK3 PE=1	218,32	10
373	Q9NRY4	RHG35_HUMAN Rho GTPase-activating protein 35 OS=Homo sapiens OX=9606 GN=AF	188,79	10
374	Q9NYF8	BCLF1_HUMAN Bcl-2-associated transcription factor 1 OS=Homo sapiens OX=9606 GN	304,23	10
375	Q16595	FRDA_HUMAN Frataxin_ mitochondrial OS=Homo sapiens OX=9606 GN=FXN PE=1 SV=	220,65	10
376	Q15147	PLCB4_HUMAN 1-phosphatidylinositol 4_5-bisphosphate phosphodiesterase beta-4 O	287,48	10
377	Q9ULV0	MYO5B_HUMAN Unconventional myosin-Vb OS=Homo sapiens OX=9606 GN=MYO5B	319,1	10
378	Q9H0H5	RGAP1_HUMAN Rac GTPase-activating protein 1 OS=Homo sapiens OX=9606 GN=RAC	253,33	10
379	P60228	EIF3E_HUMAN Eukaryotic translation initiation factor 3 subunit E OS=Homo sapiens O	198,81	10
380	Q9HCM1	K1551_HUMAN Uncharacterized protein KIAA1551 OS=Homo sapiens OX=9606 GN=KI	268,45	10
381	Q15124	PGM5_HUMAN Phosphoglucomutase-like protein 5 OS=Homo sapiens OX=9606 GN=F	228,14	10
382	Q9BPZ2	SPI2B_HUMAN Spindlin-2B OS=Homo sapiens OX=9606 GN=SPIN2B PE=1 SV=1	259,05	10
383	Q96I24	FUBP3_HUMAN Far upstream element-binding protein 3 OS=Homo sapiens OX=9606 (	325,79	10
384	Q9H6S0	YTDC2_HUMAN Probable ATP-dependent RNA helicase YTHDC2 OS=Homo sapiens OX	395,02	10
385	Q7Z6B0	CCD91_HUMAN Coiled-coil domain-containing protein 91 OS=Homo sapiens OX=9606	377,65	10
386	P50148	GNAQ_HUMAN Guanine nucleotide-binding protein G(q) subunit alpha OS=Homo sapi	312,05	10
387	O95168	NDUB4_HUMAN NADH dehydrogenase [ubiquinone] 1 beta subcomplex subunit 4 OS=	178,54	10
388	Q9BPX5	ARP5L_HUMAN Actin-related protein 2/3 complex subunit 5-like protein OS=Homo sa	447,78	10
389	Q9H3K6	BOLA2_HUMAN BolA-like protein 2 OS=Homo sapiens OX=9606 GN=BOLA2 PE=1 SV=1	508,34	10
390	Q14376	GALE_HUMAN UDP-glucose 4-epimerase OS=Homo sapiens OX=9606 GN=GALE PE=1 !	333,31	10
391	Q8TAD7	OCC1_HUMAN Overexpressed in colon carcinoma 1 protein OS=Homo sapiens OX=96(	834,77	10

392	Q9BWD1	THIC_HUMAN Acetyl-CoA acetyltransferase_ cytosolic OS=Homo sapiens OX=9606 GN	264,62	10
393	Q5JR59	MTUS2_HUMAN Microtubule-associated tumor suppressor candidate 2 OS=Homo sap	279,38	10
394	Q96KT0	FAAS1_HUMAN Uncharacterized protein FAM167A-AS1 OS=Homo sapiens OX=9606 G	237,64	10
395	Q16531	DDB1_HUMAN DNA damage-binding protein 1 OS=Homo sapiens OX=9606 GN=DDB1	199,3	10
396	Q16890	TPD53_HUMAN Tumor protein D53 OS=Homo sapiens OX=9606 GN=TPD52L1 PE=1 SV	271,5	10
397	P46926	GNPI1_HUMAN Glucosamine-6-phosphate isomerase 1 OS=Homo sapiens OX=9606 G	172,55	10
398	P60891	PRPS1_HUMAN Ribose-phosphate pyrophosphokinase 1 OS=Homo sapiens OX=9606 (	611,71	10
399	Q9UM07	PADI4_HUMAN Protein-arginine deiminase type-4 OS=Homo sapiens OX=9606 GN=PA	377,65	10
400	Q86TI0	TBCD1_HUMAN TBC1 domain family member 1 OS=Homo sapiens OX=9606 GN=TBC1	251,55	10
401	Q16878	CDO1_HUMAN Cysteine dioxygenase type 1 OS=Homo sapiens OX=9606 GN=CDO1 PE	154,39	10
402	O43491	E41L2_HUMAN Band 4.1-like protein 2 OS=Homo sapiens OX=9606 GN=EPB41L2 PE=1	231,46	10
403	Q9H9S3	S61A2_HUMAN Protein transport protein Sec61 subunit alpha isoform 2 OS=Homo sa	216,12	10
404	Q13243	SRSF5_HUMAN Serine/arginine-rich splicing factor 5 OS=Homo sapiens OX=9606 GN=	185,09	10
405	Q496Y0	LONF3_HUMAN LON peptidase N-terminal domain and RING finger protein 3 OS=Hom	277,35	10
406	P22061	PIMT_HUMAN Protein-L-isoaspartate(D-aspartate) O-methyltransferase OS=Homo sa	527,38	10
407	Q5JTV8	TOIP1_HUMAN Torsin-1A-interacting protein 1 OS=Homo sapiens OX=9606 GN=TOR1	218,34	10
408	Q9NUU7	DD19A_HUMAN ATP-dependent RNA helicase DDX19A OS=Homo sapiens OX=9606 G	158,83	10
409	Q4V328	GRAP1_HUMAN GRIP1-associated protein 1 OS=Homo sapiens OX=9606 GN=GRIPAP1	234,54	10
410	Q9UBX3	DIC_HUMAN Mitochondrial dicarboxylate carrier OS=Homo sapiens OX=9606 GN=SLC	446,49	10
411	Q13586	STIM1_HUMAN Stromal interaction molecule 1 OS=Homo sapiens OX=9606 GN=STIM	361,86	10
412	Q86TE4	LUZP2_HUMAN Leucine zipper protein 2 OS=Homo sapiens OX=9606 GN=LUZP2 PE=2	900,56	10
413	Q8IZ21	PHAR4_HUMAN Phosphatase and actin regulator 4 OS=Homo sapiens OX=9606 GN=P	163,64	10
414	Q9Y6R4	M3K4_HUMAN Mitogen-activated protein kinase kinase kinase 4 OS=Homo sapiens O	153,53	10
415	P00966	ASSY_HUMAN Argininosuccinate synthase OS=Homo sapiens OX=9606 GN=ASS1 PE=1	166,26	10
416	P49257	LMAN1_HUMAN Protein ERGIC-53 OS=Homo sapiens OX=9606 GN=LMAN1 PE=1 SV=2	203,15	10
417	P05161	ISG15_HUMAN Ubiquitin-like protein ISG15 OS=Homo sapiens OX=9606 GN=ISG15 PE	358,66	10
418	P61923	COPZ1_HUMAN Coatomer subunit zeta-1 OS=Homo sapiens OX=9606 GN=COPZ1 PE=	385,42	10
419	P52948	NUP98_HUMAN Nuclear pore complex protein Nup98-Nup96 OS=Homo sapiens OX=9	201,87	10
420	O95433	AHSA1_HUMAN Activator of 90 kDa heat shock protein ATPase homolog 1 OS=Homo	568,45	10
421	P15586	GNS_HUMAN N-acetylglucosamine-6-sulfatase OS=Homo sapiens OX=9606 GN=GNS P	471,22	10
422	Q13206	DDX10_HUMAN Probable ATP-dependent RNA helicase DDX10 OS=Homo sapiens OX=	317,02	10

423	P14136	GFAP_HUMAN Glial fibrillary acidic protein OS=Homo sapiens OX=9606 GN=GFAP PE=	647,74	10
424	P61916	NPC2_HUMAN NPC intracellular cholesterol transporter 2 OS=Homo sapiens OX=9606	844,7	10
425	P50402	EMD_HUMAN Emerin OS=Homo sapiens OX=9606 GN=EMD PE=1 SV=1	236,46	10
426	O76021	RL1D1_HUMAN Ribosomal L1 domain-containing protein 1 OS=Homo sapiens OX=960	271,54	10
427	P07686	HEXB_HUMAN Beta-hexosaminidase subunit beta OS=Homo sapiens OX=9606 GN=HE	431,63	10
428	Q9NUQ6	SPS2L_HUMAN SPATS2-like protein OS=Homo sapiens OX=9606 GN=SPATS2L PE=1 SV	135,83	10
429	Q96HE7	ERO1A_HUMAN ERO1-like protein alpha OS=Homo sapiens OX=9606 GN=ERO1A PE=1	277,4	10
430	P07316	CRGB_HUMAN Gamma-crystallin B OS=Homo sapiens OX=9606 GN=CRYGB PE=1 SV=3	264,81	10
431	Q9UBT2	SAE2_HUMAN SUMO-activating enzyme subunit 2 OS=Homo sapiens OX=9606 GN=UE	262,36	10
432	Q99551	MTEF1_HUMAN Transcription termination factor 1_ mitochondrial OS=Homo sapiens	337,68	10
433	P61601	NCALD_HUMAN Neurocalcin-delta OS=Homo sapiens OX=9606 GN=NCALD PE=1 SV=2	286,7	6,55
434	O76003	GLRX3_HUMAN Glutaredoxin-3 OS=Homo sapiens OX=9606 GN=GLRX3 PE=1 SV=2	243,26	3,16
435	P30043	BLVRB_HUMAN Flavin reductase (NADPH) OS=Homo sapiens OX=9606 GN=BLVRB PE=	214,72	2,83
436	Q6DRA6	H2B2D_HUMAN Putative histone H2B type 2-D OS=Homo sapiens OX=9606 GN=HIST2	344,48	2,66
437	Q6IS14	IF5AL_HUMAN Eukaryotic translation initiation factor 5A-1-like OS=Homo sapiens OX=	264,34	2,64
438	Q6DN03	H2B2C_HUMAN Putative histone H2B type 2-C OS=Homo sapiens OX=9606 GN=HIST2I	344,48	2,61
439	P02795	MT2_HUMAN Metallothionein-2 OS=Homo sapiens OX=9606 GN=MT2A PE=1 SV=1	5904,11	2,59
440	P04732	MT1E_HUMAN Metallothionein-1E OS=Homo sapiens OX=9606 GN=MT1E PE=1 SV=1	5904,11	2,51
441	P49419	AL7A1_HUMAN Alpha-aminoadipic semialdehyde dehydrogenase OS=Homo sapiens C	191,17	2,48
442	P13640	MT1G_HUMAN Metallothionein-1G OS=Homo sapiens OX=9606 GN=MT1G PE=1 SV=2	5904,11	2,46
443	P80297	MT1X_HUMAN Metallothionein-1X OS=Homo sapiens OX=9606 GN=MT1X PE=1 SV=1	5904,11	2,46
444	P30626	SORCN_HUMAN Sorcin OS=Homo sapiens OX=9606 GN=SRI PE=1 SV=1	271,97	2,46
445	P0CG47	UBB_HUMAN Polyubiquitin-B OS=Homo sapiens OX=9606 GN=UBB PE=1 SV=1	4820,3	2,41
446	Q8N339	MT1M_HUMAN Metallothionein-1M OS=Homo sapiens OX=9606 GN=MT1M PE=3 SV	5904,11	2,41
447	P02786	TFR1_HUMAN Transferrin receptor protein 1 OS=Homo sapiens OX=9606 GN=TFRC PE	215,99	2,41
448	P62987	RL40_HUMAN Ubiquitin-60S ribosomal protein L40 OS=Homo sapiens OX=9606 GN=U	4820,3	2,39
449	P62979	RS27A_HUMAN Ubiquitin-40S ribosomal protein S27a OS=Homo sapiens OX=9606 GN	4820,3	2,36
450	P0CG48	UBC_HUMAN Polyubiquitin-C OS=Homo sapiens OX=9606 GN=UBC PE=1 SV=3	4820,3	2,36
451	P07108	ACBP_HUMAN Acyl-CoA-binding protein OS=Homo sapiens OX=9606 GN=DBI PE=1 SV	336,57	2,27
452	Q9UBL6	CPNE7_HUMAN Copine-7 OS=Homo sapiens OX=9606 GN=CPNE7 PE=1 SV=1	460,1	2,25
453	P23381	SYWC_HUMAN Tryptophan--tRNA ligase_ cytoplasmic OS=Homo sapiens OX=9606 GN	238,84	2,23

454	P09874	PARP1_HUMAN Poly [ADP-ribose] polymerase 1 OS=Homo sapiens OX=9606 GN=PARI	241,55	2,23
455	Q9Y265	RUVB1_HUMAN RuvB-like 1 OS=Homo sapiens OX=9606 GN=RUVBL1 PE=1 SV=1	261,97	2,16
456	P63241	IF5A1_HUMAN Eukaryotic translation initiation factor 5A-1 OS=Homo sapiens OX=960	221,02	2,10
457	P49841	GSK3B_HUMAN Glycogen synthase kinase-3 beta OS=Homo sapiens OX=9606 GN=GSK	212,94	2,10
458	P04626	ERBB2_HUMAN Receptor tyrosine-protein kinase erbB-2 OS=Homo sapiens OX=9606 (	179,71	2,08
459	Q96JA4	M4A14_HUMAN Membrane-spanning 4-domains subfamily A member 14 OS=Homo s	217,6	2,08
460	O00764	PDXK_HUMAN Pyridoxal kinase OS=Homo sapiens OX=9606 GN=PDXK PE=1 SV=1	343,51	2,05
461	P56537	IF6_HUMAN Eukaryotic translation initiation factor 6 OS=Homo sapiens OX=9606 GN=	471,45	2,03
462	O95336	6PGL_HUMAN 6-phosphogluconolactonase OS=Homo sapiens OX=9606 GN=PGLS PE=	254,02	2,03
463	O60664	PLIN3_HUMAN Perilipin-3 OS=Homo sapiens OX=9606 GN=PLIN3 PE=1 SV=3	251,48	1,99
464	P07858	CATB_HUMAN Cathepsin B OS=Homo sapiens OX=9606 GN=CTSB PE=1 SV=3	225,8	1,97
465	P13489	RINI_HUMAN Ribonuclease inhibitor OS=Homo sapiens OX=9606 GN=RNH1 PE=1 SV=2	262,78	1,97
466	P30740	ILEU_HUMAN Leukocyte elastase inhibitor OS=Homo sapiens OX=9606 GN=SERPINB1	336,47	1,95
467	Q99733	NP1L4_HUMAN Nucleosome assembly protein 1-like 4 OS=Homo sapiens OX=9606 GN	249,48	1,93
468	O95571	ETHE1_HUMAN Persulfide dioxygenase ETHE1_ mitochondrial OS=Homo sapiens OX=9	203,17	1,93
469	P63208	SKP1_HUMAN S-phase kinase-associated protein 1 OS=Homo sapiens OX=9606 GN=SK	410,8	1,90
470	P50453	SPB9_HUMAN Serpin B9 OS=Homo sapiens OX=9606 GN=SERPINB9 PE=1 SV=1	223,46	1,90
471	P61604	CH10_HUMAN 10 kDa heat shock protein_ mitochondrial OS=Homo sapiens OX=9606	4066,88	1,88
472	P54709	AT1B3_HUMAN Sodium/potassium-transporting ATPase subunit beta-3 OS=Homo sap	280,96	1,88
473	P10768	ESTD_HUMAN S-formylglutathione hydrolase OS=Homo sapiens OX=9606 GN=ESD PE=	923,95	1,86
474	P40121	CAPG_HUMAN Macrophage-capping protein OS=Homo sapiens OX=9606 GN=CAPG PE	1032,9	1,84
475	O15427	MOT4_HUMAN Monocarboxylate transporter 4 OS=Homo sapiens OX=9606 GN=SLC16	297,36	1,84
476	Q58FF6	H90B4_HUMAN Putative heat shock protein HSP 90-beta 4 OS=Homo sapiens OX=960	2526,77	1,82
477	P54819	KAD2_HUMAN Adenylate kinase 2_ mitochondrial OS=Homo sapiens OX=9606 GN=AK	281,78	1,82
478	P61513	RL37A_HUMAN 60S ribosomal protein L37a OS=Homo sapiens OX=9606 GN=RPL37A F	228,2	1,82
479	O75368	SH3L1_HUMAN SH3 domain-binding glutamic acid-rich-like protein OS=Homo sapiens	525,72	1,80
480	O00487	PSDE_HUMAN 26S proteasome non-ATPase regulatory subunit 14 OS=Homo sapiens (	242,84	1,80
481	P27482	CALL3_HUMAN Calmodulin-like protein 3 OS=Homo sapiens OX=9606 GN=CALML3 PE	263,21	1,79
482	P20700	LMNB1_HUMAN Lamin-B1 OS=Homo sapiens OX=9606 GN=LMNB1 PE=1 SV=2	251,72	1,79
483	Q07960	RHG01_HUMAN Rho GTPase-activating protein 1 OS=Homo sapiens OX=9606 GN=ARH	295,16	1,77
484	P63220	RS21_HUMAN 40S ribosomal protein S21 OS=Homo sapiens OX=9606 GN=RPS21 PE=1	688,2	1,77

485	Q58FG0	HS905_HUMAN Putative heat shock protein HSP 90-alpha A5 OS=Homo sapiens OX=96	1293,33	1,75
486	P84098	RL19_HUMAN 60S ribosomal protein L19 OS=Homo sapiens OX=9606 GN=RPL19 PE=1	688,13	1,75
487	Q9UKA9	PTBP2_HUMAN Polypyrimidine tract-binding protein 2 OS=Homo sapiens OX=9606 GN	266,89	1,75
488	Q08211	DHX9_HUMAN ATP-dependent RNA helicase A OS=Homo sapiens OX=9606 GN=DHX9	243,25	1,73
489	P00491	PNPH_HUMAN Purine nucleoside phosphorylase OS=Homo sapiens OX=9606 GN=PNP	553,65	1,73
490	P32320	CDD_HUMAN Cytidine deaminase OS=Homo sapiens OX=9606 GN=CDA PE=1 SV=2	671,76	1,73
491	P00403	COX2_HUMAN Cytochrome c oxidase subunit 2 OS=Homo sapiens OX=9606 GN=MT-C	227,73	1,73
492	P25786	PSA1_HUMAN Proteasome subunit alpha type-1 OS=Homo sapiens OX=9606 GN=PSM	490,62	1,72
493	Q15181	IPYR_HUMAN Inorganic pyrophosphatase OS=Homo sapiens OX=9606 GN=PPA1 PE=1	219,72	1,72
494	P51858	HDGF_HUMAN Hepatoma-derived growth factor OS=Homo sapiens OX=9606 GN=HDG	740,35	1,72
495	Q96L46	CPNS2_HUMAN Calpain small subunit 2 OS=Homo sapiens OX=9606 GN=CAPNS2 PE=2	406,65	1,72
496	P30505	1C08_HUMAN HLA class I histocompatibility antigen_ Cw-8 alpha chain OS=Homo sapi	236,09	1,70
497	P11766	ADHX_HUMAN Alcohol dehydrogenase class-3 OS=Homo sapiens OX=9606 GN=ADH5	609,38	1,70
498	P26639	SYTC_HUMAN Threonine--tRNA ligase_ cytoplasmic OS=Homo sapiens OX=9606 GN=T	207,97	1,70
499	O15144	ARPC2_HUMAN Actin-related protein 2/3 complex subunit 2 OS=Homo sapiens OX=96	216,54	1,70
500	P43487	RANG_HUMAN Ran-specific GTPase-activating protein OS=Homo sapiens OX=9606 GN	612,67	1,70
501	P17081	RHOQ_HUMAN Rho-related GTP-binding protein RhoQ OS=Homo sapiens OX=9606 GN	426,89	1,68
502	O00232	PSD12_HUMAN 26S proteasome non-ATPase regulatory subunit 12 OS=Homo sapiens	190,34	1,68
503	P49368	TCPG_HUMAN T-complex protein 1 subunit gamma OS=Homo sapiens OX=9606 GN=C	989,03	1,68
504	P62854	RS26_HUMAN 40S ribosomal protein S26 OS=Homo sapiens OX=9606 GN=RPS26 PE=1	714,86	1,67
505	P84095	RHOG_HUMAN Rho-related GTP-binding protein RhoG OS=Homo sapiens OX=9606 GN	426,89	1,67
506	P10321	1C07_HUMAN HLA class I histocompatibility antigen_ Cw-7 alpha chain OS=Homo sapi	236,09	1,67
507	Q5JNZ5	RS26L_HUMAN Putative 40S ribosomal protein S26-like 1 OS=Homo sapiens OX=9606	426,72	1,67
508	P62330	ARF6_HUMAN ADP-ribosylation factor 6 OS=Homo sapiens OX=9606 GN=ARF6 PE=1 S	281,75	1,65
509	P27695	APEX1_HUMAN DNA-(apurinic or apyrimidinic site) lyase OS=Homo sapiens OX=9606 G	871,4	1,65
510	P16152	CBR1_HUMAN Carbonyl reductase [NADPH] 1 OS=Homo sapiens OX=9606 GN=CBR1 P	1470,5	1,65
511	Q92945	FUBP2_HUMAN Far upstream element-binding protein 2 OS=Homo sapiens OX=9606 G	286,66	1,65
512	Q9TNN7	1C05_HUMAN HLA class I histocompatibility antigen_ Cw-5 alpha chain OS=Homo sapi	236,09	1,65
513	P62333	PRS10_HUMAN 26S proteasome regulatory subunit 10B OS=Homo sapiens OX=9606 G	262,82	1,63
514	P14868	SYDC_HUMAN Aspartate--tRNA ligase_ cytoplasmic OS=Homo sapiens OX=9606 GN=D	295,07	1,63
515	Q96FQ6	S10AG_HUMAN Protein S100-A16 OS=Homo sapiens OX=9606 GN=S100A16 PE=1 SV=	3769,79	1,63

516	Q9Y230	RUVB2_HUMAN RuvB-like 2 OS=Homo sapiens OX=9606 GN=RUVBL2 PE=1 SV=3	258,62	1,63
517	Q58FF7	H90B3_HUMAN Putative heat shock protein HSP 90-beta-3 OS=Homo sapiens OX=960	3517,47	1,62
518	P18077	RL35A_HUMAN 60S ribosomal protein L35a OS=Homo sapiens OX=9606 GN=RPL35A F	327,22	1,62
519	P10599	THIO_HUMAN Thioredoxin OS=Homo sapiens OX=9606 GN=TXN PE=1 SV=3	2512,85	1,62
520	O60493	SNX3_HUMAN Sorting nexin-3 OS=Homo sapiens OX=9606 GN=SNX3 PE=1 SV=3	440,39	1,62
521	Q99584	S10AD_HUMAN Protein S100-A13 OS=Homo sapiens OX=9606 GN=S100A13 PE=1 SV=	397,6	1,62
522	Q9ULV4	COR1C_HUMAN Coronin-1C OS=Homo sapiens OX=9606 GN=CORO1C PE=1 SV=1	289,68	1,62
523	Q16543	CDC37_HUMAN Hsp90 co-chaperone Cdc37 OS=Homo sapiens OX=9606 GN=CDC37 P	221,3	1,62
524	O43242	PSMD3_HUMAN 26S proteasome non-ATPase regulatory subunit 3 OS=Homo sapiens	168,22	1,62
525	P31930	QCR1_HUMAN Cytochrome b-c1 complex subunit 1_ mitochondrial OS=Homo sapiens	433,65	1,62
526	P07602	SAP_HUMAN Prosaposin OS=Homo sapiens OX=9606 GN=PSAP PE=1 SV=2	422,16	1,60
527	P40926	MDHM_HUMAN Malate dehydrogenase_ mitochondrial OS=Homo sapiens OX=9606 (	1337,68	1,60
528	Q04760	LGUL_HUMAN Lactoylglutathione lyase OS=Homo sapiens OX=9606 GN=GLO1 PE=1 S\	962,21	1,60
529	O00299	CLIC1_HUMAN Chloride intracellular channel protein 1 OS=Homo sapiens OX=9606 GN	943,31	1,60
530	Q9H4E5	RHOJ_HUMAN Rho-related GTP-binding protein RhoJ OS=Homo sapiens OX=9606 GN=	426,89	1,60
531	P05386	RLA1_HUMAN 60S acidic ribosomal protein P1 OS=Homo sapiens OX=9606 GN=RPLP1	2406,26	1,60
532	P78330	SERB_HUMAN Phosphoserine phosphatase OS=Homo sapiens OX=9606 GN=PSPH PE=	607,21	1,58
533	P30044	PRDX5_HUMAN Peroxiredoxin-5_ mitochondrial OS=Homo sapiens OX=9606 GN=PRD	913,12	1,58
534	P60953	CDC42_HUMAN Cell division control protein 42 homolog OS=Homo sapiens OX=9606 (	1122,52	1,58
535	P28066	PSA5_HUMAN Proteasome subunit alpha type-5 OS=Homo sapiens OX=9606 GN=PSM	282,03	1,58
536	Q01130	SRSF2_HUMAN Serine/arginine-rich splicing factor 2 OS=Homo sapiens OX=9606 GN=S	2421,12	1,57
537	P00441	SODC_HUMAN Superoxide dismutase [Cu-Zn] OS=Homo sapiens OX=9606 GN=SOD1 P	1146,76	1,57
538	Q14764	MVP_HUMAN Major vault protein OS=Homo sapiens OX=9606 GN=MVP PE=1 SV=4	364,04	1,57
539	Q96KP4	CNDP2_HUMAN Cytosolic non-specific dipeptidase OS=Homo sapiens OX=9606 GN=CI	395,02	1,57
540	P07339	CATD_HUMAN Cathepsin D OS=Homo sapiens OX=9606 GN=CTSD PE=1 SV=1	1804,98	1,57
541	P05787	K2C8_HUMAN Keratin_ type II cytoskeletal 8 OS=Homo sapiens OX=9606 GN=KRT8 PE	904,65	1,55
542	P36406	TRI23_HUMAN E3 ubiquitin-protein ligase TRIM23 OS=Homo sapiens OX=9606 GN=TR	209,88	1,55
543	P09104	ENOG_HUMAN Gamma-enolase OS=Homo sapiens OX=9606 GN=ENO2 PE=1 SV=3	1184,8	1,55
544	P0DN37	PAL4G_HUMAN Peptidyl-prolyl cis-trans isomerase A-like 4G OS=Homo sapiens OX=96	608,22	1,55
545	Q6NZI2	CAVN1_HUMAN Caveolae-associated protein 1 OS=Homo sapiens OX=9606 GN=CAVII	832,94	1,55
546	P60900	PSA6_HUMAN Proteasome subunit alpha type-6 OS=Homo sapiens OX=9606 GN=PSM	919,94	1,55

547	Q9NVA2	SEP11_HUMAN Septin-11 OS=Homo sapiens OX=9606 GN=SEPT11 PE=1 SV=3	390,66	1,55
548	A0A075B759	PAL4E_HUMAN Peptidyl-prolyl cis-trans isomerase A-like 4E OS=Homo sapiens OX=96	608,22	1,55
549	P20073	ANXA7_HUMAN Annexin A7 OS=Homo sapiens OX=9606 GN=ANXA7 PE=1 SV=3	396,24	1,55
550	A8MTJ3	GNAT3_HUMAN Guanine nucleotide-binding protein G(t) subunit alpha-3 OS=Homo s	326,71	1,55
551	P49458	SRP09_HUMAN Signal recognition particle 9 kDa protein OS=Homo sapiens OX=9606	756,36	1,55
552	P49448	DHE4_HUMAN Glutamate dehydrogenase 2_ mitochondrial OS=Homo sapiens OX=96	217,21	1,54
553	P49411	EFTU_HUMAN Elongation factor Tu_ mitochondrial OS=Homo sapiens OX=9606 GN=T	1357,26	1,54
554	P32969	RL9_HUMAN 60S ribosomal protein L9 OS=Homo sapiens OX=9606 GN=RPL9 PE=1 SV	806,68	1,54
555	Q99714	HCD2_HUMAN 3-hydroxyacyl-CoA dehydrogenase type-2 OS=Homo sapiens OX=9606	581,49	1,54
556	P06744	G6PI_HUMAN Glucose-6-phosphate isomerase OS=Homo sapiens OX=9606 GN=GPI P	437,8	1,54
557	P19338	NUCL_HUMAN Nucleolin OS=Homo sapiens OX=9606 GN=NCL PE=1 SV=3	971,44	1,54
558	O00148	DX39A_HUMAN ATP-dependent RNA helicase DDX39A OS=Homo sapiens OX=9606 G	446,84	1,54
559	P08754	GNAI3_HUMAN Guanine nucleotide-binding protein G(k) subunit alpha OS=Homo sap	545,58	1,54
560	P08708	RS17_HUMAN 40S ribosomal protein S17 OS=Homo sapiens OX=9606 GN=RPS17 PE=1	1675,88	1,54
561	P11488	GNAT1_HUMAN Guanine nucleotide-binding protein G(t) subunit alpha-1 OS=Homo s	361,11	1,54
562	O75947	ATP5H_HUMAN ATP synthase subunit d_ mitochondrial OS=Homo sapiens OX=9606 G	1407,96	1,54
563	P61956	SUMO2_HUMAN Small ubiquitin-related modifier 2 OS=Homo sapiens OX=9606 GN=S	2438,91	1,54
564	P62633	CNBP_HUMAN Cellular nucleic acid-binding protein OS=Homo sapiens OX=9606 GN=C	542,37	1,54
565	P13693	TCTP_HUMAN Translationally-controlled tumor protein OS=Homo sapiens OX=9606 G	230,74	1,52
566	P31939	PUR9_HUMAN Bifunctional purine biosynthesis protein PURH OS=Homo sapiens OX=	388,72	1,52
567	P78371	TCPB_HUMAN T-complex protein 1 subunit beta OS=Homo sapiens OX=9606 GN=CCT	292,77	1,52
568	P51148	RAB5C_HUMAN Ras-related protein Rab-5C OS=Homo sapiens OX=9606 GN=RAB5C P	1289,98	1,52
569	P52565	GDIR1_HUMAN Rho GDP-dissociation inhibitor 1 OS=Homo sapiens OX=9606 GN=ARH	2694,66	1,52
570	Q9BRL6	SRSF8_HUMAN Serine/arginine-rich splicing factor 8 OS=Homo sapiens OX=9606 GN=	2202,81	1,52
571	P46779	RL28_HUMAN 60S ribosomal protein L28 OS=Homo sapiens OX=9606 GN=RPL28 PE=1	1001,85	1,52
572	Q9Y277	VDAC3_HUMAN Voltage-dependent anion-selective channel protein 3 OS=Homo sapi	840,04	1,52
573	F5H284	PAL4D_HUMAN Peptidyl-prolyl cis-trans isomerase A-like 4D OS=Homo sapiens OX=96	608,22	1,52
574	P08865	RSSA_HUMAN 40S ribosomal protein SA OS=Homo sapiens OX=9606 GN=RPSA PE=1 S	1838,09	1,52
575	P05556	ITB1_HUMAN Integrin beta-1 OS=Homo sapiens OX=9606 GN=ITGB1 PE=1 SV=2	393,14	1,52
576	P52907	CAZA1_HUMAN F-actin-capping protein subunit alpha-1 OS=Homo sapiens OX=9606	431,67	1,52
577	P04632	CPNS1_HUMAN Calpain small subunit 1 OS=Homo sapiens OX=9606 GN=CAPNS1 PE=1	439,71	1,52

578	P24539	AT5F1_HUMAN ATP synthase F(0) complex subunit B1_ mitochondrial OS=Homo sapie	211,53	1,52
579	A0A0B4J2A2	PAL4C_HUMAN Peptidyl-prolyl cis-trans isomerase A-like 4C OS=Homo sapiens OX=96	608,22	1,51
580	P00558	PGK1_HUMAN Phosphoglycerate kinase 1 OS=Homo sapiens OX=9606 GN=PGK1 PE=1	2941,76	1,51
581	P60763	RAC3_HUMAN Ras-related C3 botulinum toxin substrate 3 OS=Homo sapiens OX=960	702,34	1,51
582	P50991	TCPD_HUMAN T-complex protein 1 subunit delta OS=Homo sapiens OX=9606 GN=CCT	318,74	1,51
583	P0DN26	PAL4F_HUMAN Peptidyl-prolyl cis-trans isomerase A-like 4F OS=Homo sapiens OX=96	608,22	1,51
584	P30084	ECHM_HUMAN Enoyl-CoA hydratase_ mitochondrial OS=Homo sapiens OX=9606 GN=	1048,87	1,51
585	P14678	RSMB_HUMAN Small nuclear ribonucleoprotein-associated proteins B and B' OS=Hom	250,14	1,51
586	P30456	1A43_HUMAN HLA class I histocompatibility antigen_ A-43 alpha chain OS=Homo sapi	621,38	1,51
587	P52597	HNRPF_HUMAN Heterogeneous nuclear ribonucleoprotein F OS=Homo sapiens OX=96	344,2	1,49
588	P10316	1A69_HUMAN HLA class I histocompatibility antigen_ A-69 alpha chain OS=Homo sapi	996,81	1,49
589	Q9Y696	CLIC4_HUMAN Chloride intracellular channel protein 4 OS=Homo sapiens OX=9606 GN	803,7	1,49
590	P35613	BASI_HUMAN Basigin OS=Homo sapiens OX=9606 GN=BSG PE=1 SV=2	407,65	1,49
591	P30457	1A66_HUMAN HLA class I histocompatibility antigen_ A-66 alpha chain OS=Homo sapi	775,21	1,49
592	P30450	1A26_HUMAN HLA class I histocompatibility antigen_ A-26 alpha chain OS=Homo sapi	621,38	1,49
593	Q9UJZ1	STML2_HUMAN Stomatin-like protein 2_ mitochondrial OS=Homo sapiens OX=9606 G	371,86	1,49
594	P12429	ANXA3_HUMAN Annexin A3 OS=Homo sapiens OX=9606 GN=ANXA3 PE=1 SV=3	399,64	1,49
595	P35232	PHB_HUMAN Prohibitin OS=Homo sapiens OX=9606 GN=PHB PE=1 SV=1	308,34	1,49
596	P01892	1A02_HUMAN HLA class I histocompatibility antigen_ A-2 alpha chain OS=Homo sapie	1022,85	1,49
597	P01891	1A68_HUMAN HLA class I histocompatibility antigen_ A-68 alpha chain OS=Homo sapi	1145,58	1,49
598	P30086	PEBP1_HUMAN Phosphatidylethanolamine-binding protein 1 OS=Homo sapiens OX=9	2236,68	1,49
599	P30048	PRDX3_HUMAN Thioredoxin-dependent peroxide reductase_ mitochondrial OS=Hom	1702,64	1,49
600	P61970	NTF2_HUMAN Nuclear transport factor 2 OS=Homo sapiens OX=9606 GN=NUTF2 PE=	2614,22	1,49
601	P15531	NDKA_HUMAN Nucleoside diphosphate kinase A OS=Homo sapiens OX=9606 GN=NM	2998,41	1,48
602	P61204	ARF3_HUMAN ADP-ribosylation factor 3 OS=Homo sapiens OX=9606 GN=ARF3 PE=1 S	2367,35	1,48
603	P84077	ARF1_HUMAN ADP-ribosylation factor 1 OS=Homo sapiens OX=9606 GN=ARF1 PE=1 S	2424,22	1,48
604	Q9UNX3	RL26L_HUMAN 60S ribosomal protein L26-like 1 OS=Homo sapiens OX=9606 GN=RPL2	498,12	1,48
605	Q5TZA2	CROCC_HUMAN Rootletin OS=Homo sapiens OX=9606 GN=CROCC PE=1 SV=1	246,16	1,48
606	P39023	RL3_HUMAN 60S ribosomal protein L3 OS=Homo sapiens OX=9606 GN=RPL3 PE=1 SV=	648,32	1,48
607	P30459	1A74_HUMAN HLA class I histocompatibility antigen_ A-74 alpha chain OS=Homo sapi	917,77	1,48
608	P30453	1A34_HUMAN HLA class I histocompatibility antigen_ A-34 alpha chain OS=Homo sapi	880,07	1,48

609	P0DME0	SETLP_HUMAN Protein SETSIP OS=Homo sapiens OX=9606 GN=SETSIP PE=1 SV=1	758,35	1,48
610	O00231	PSD11_HUMAN 26S proteasome non-ATPase regulatory subunit 11 OS=Homo sapiens	251,6	1,48
611	P30050	RL12_HUMAN 60S ribosomal protein L12 OS=Homo sapiens OX=9606 GN=RPL12 PE=1	1419,34	1,48
612	P23526	SAHH_HUMAN Adenosylhomocysteinase OS=Homo sapiens OX=9606 GN=AHCY PE=1	1124,45	1,48
613	P04439	1A03_HUMAN HLA class I histocompatibility antigen_ A-3 alpha chain OS=Homo sapie	812,91	1,48
614	P13746	1A11_HUMAN HLA class I histocompatibility antigen_ A-11 alpha chain OS=Homo sapi	786,87	1,48
615	P0DP25	CALM3_HUMAN Calmodulin-3 OS=Homo sapiens OX=9606 GN=CALM3 PE=1 SV=1	2082,95	1,48
616	P26368	U2AF2_HUMAN Splicing factor U2AF 65 kDa subunit OS=Homo sapiens OX=9606 GN=U	169,18	1,48
617	O43396	TXNL1_HUMAN Thioredoxin-like protein 1 OS=Homo sapiens OX=9606 GN=TXNL1 PE=	277,78	1,48
618	Q07021	C1QBP_HUMAN Complement component 1 Q subcomponent-binding protein_ mitoch	215,21	1,48
619	Q14974	IMB1_HUMAN Importin subunit beta-1 OS=Homo sapiens OX=9606 GN=KPNB1 PE=1 S	168,55	1,46
620	P38646	GRP75_HUMAN Stress-70 protein_ mitochondrial OS=Homo sapiens OX=9606 GN=HSI	2565,66	1,46
621	Q15365	PCBP1_HUMAN Poly(rC)-binding protein 1 OS=Homo sapiens OX=9606 GN=PCBP1 PE=	2230,75	1,46
622	P18462	1A25_HUMAN HLA class I histocompatibility antigen_ A-25 alpha chain OS=Homo sapi	621,38	1,46
623	P07205	PGK2_HUMAN Phosphoglycerate kinase 2 OS=Homo sapiens OX=9606 GN=PGK2 PE=1	614,34	1,46
624	Q01105	SET_HUMAN Protein SET OS=Homo sapiens OX=9606 GN=SET PE=1 SV=3	1069,26	1,46
625	P20339	RAB5A_HUMAN Ras-related protein Rab-5A OS=Homo sapiens OX=9606 GN=RAB5A P	997,78	1,46
626	P04843	RPN1_HUMAN Dolichyl-diphosphooligosaccharide--protein glycosyltransferase subuni	656,11	1,46
627	P16189	1A31_HUMAN HLA class I histocompatibility antigen_ A-31 alpha chain OS=Homo sapi	880,07	1,46
628	Q14103	HNRPD_HUMAN Heterogeneous nuclear ribonucleoprotein D0 OS=Homo sapiens OX=	1709,94	1,46
629	P24752	THIL_HUMAN Acetyl-CoA acetyltransferase_ mitochondrial OS=Homo sapiens OX=960	355,21	1,46
630	P20618	PSB1_HUMAN Proteasome subunit beta type-1 OS=Homo sapiens OX=9606 GN=PSMB	405,4	1,46
631	P30049	ATPD_HUMAN ATP synthase subunit delta_ mitochondrial OS=Homo sapiens OX=960	357,09	1,46
632	P04899	GNAI2_HUMAN Guanine nucleotide-binding protein G(i) subunit alpha-2 OS=Homo sa	454,57	1,46
633	P63173	RL38_HUMAN 60S ribosomal protein L38 OS=Homo sapiens OX=9606 GN=RPL38 PE=1	467,22	1,46
634	P55209	NP1L1_HUMAN Nucleosome assembly protein 1-like 1 OS=Homo sapiens OX=9606 GN	1019,5	1,45
635	P30512	1A29_HUMAN HLA class I histocompatibility antigen_ A-29 alpha chain OS=Homo sapi	726,24	1,45
636	C9JRZ8	AK1BF_HUMAN Aldo-keto reductase family 1 member B15 OS=Homo sapiens OX=960	534,68	1,45
637	Q13740	CD166_HUMAN CD166 antigen OS=Homo sapiens OX=9606 GN=ALCAM PE=1 SV=2	265,93	1,45
638	P16190	1A33_HUMAN HLA class I histocompatibility antigen_ A-33 alpha chain OS=Homo sapi	880,07	1,45
639	P16188	1A30_HUMAN HLA class I histocompatibility antigen_ A-30 alpha chain OS=Homo sapi	880,07	1,45

640	P0DP23	CALM1_HUMAN Calmodulin-1 OS=Homo sapiens OX=9606 GN=CALM1 PE=1 SV=1	2082,95	1,45
641	P62753	RS6_HUMAN 40S ribosomal protein S6 OS=Homo sapiens OX=9606 GN=RPS6 PE=1 SV=	1034,4	1,45
642	Q14019	COTL1_HUMAN Coactosin-like protein OS=Homo sapiens OX=9606 GN=COTL1 PE=1 SV	602,02	1,45
643	P62942	FKB1A_HUMAN Peptidyl-prolyl cis-trans isomerase FKBP1A OS=Homo sapiens OX=960	1058,17	1,45
644	O75131	CPNE3_HUMAN Copine-3 OS=Homo sapiens OX=9606 GN=CPNE3 PE=1 SV=1	174,09	1,45
645	P63096	GNAI1_HUMAN Guanine nucleotide-binding protein G(i) subunit alpha-1 OS=Homo sa	391,58	1,45
646	Q13310	PABP4_HUMAN Polyadenylate-binding protein 4 OS=Homo sapiens OX=9606 GN=PAB	322,01	1,45
647	P63162	RSMN_HUMAN Small nuclear ribonucleoprotein-associated protein N OS=Homo sapie	250,14	1,45
648	P62495	ERF1_HUMAN Eukaryotic peptide chain release factor subunit 1 OS=Homo sapiens OX	205,35	1,45
649	O75626	PRDM1_HUMAN PR domain zinc finger protein 1 OS=Homo sapiens OX=9606 GN=PRD	328,72	1,45
650	O43776	SYNC_HUMAN Asparagine--tRNA ligase_ cytoplasmic OS=Homo sapiens OX=9606 GN=	392,67	1,43
651	P05455	LA_HUMAN Lupus La protein OS=Homo sapiens OX=9606 GN=SSB PE=1 SV=2	504,56	1,43
652	P31943	HNRH1_HUMAN Heterogeneous nuclear ribonucleoprotein H OS=Homo sapiens OX=9	433,38	1,43
653	P26006	ITA3_HUMAN Integrin alpha-3 OS=Homo sapiens OX=9606 GN=ITGA3 PE=1 SV=5	689,33	1,43
654	Q15366	PCBP2_HUMAN Poly(rC)-binding protein 2 OS=Homo sapiens OX=9606 GN=PCBP2 PE=	2315,47	1,43
655	P09429	HMGB1_HUMAN High mobility group protein B1 OS=Homo sapiens OX=9606 GN=HM	2429,55	1,43
656	P00505	AATM_HUMAN Aspartate aminotransferase_ mitochondrial OS=Homo sapiens OX=96	1532,07	1,43
657	Q6EEV6	SUMO4_HUMAN Small ubiquitin-related modifier 4 OS=Homo sapiens OX=9606 GN=S	2438,91	1,43
658	P21796	VDAC1_HUMAN Voltage-dependent anion-selective channel protein 1 OS=Homo sapie	1532,45	1,43
659	P26583	HMGB2_HUMAN High mobility group protein B2 OS=Homo sapiens OX=9606 GN=HM	2489,34	1,43
660	P0DP24	CALM2_HUMAN Calmodulin-2 OS=Homo sapiens OX=9606 GN=CALM2 PE=1 SV=1	2082,95	1,43
661	P25398	RS12_HUMAN 40S ribosomal protein S12 OS=Homo sapiens OX=9606 GN=RPS12 PE=1	2191,05	1,43
662	Q16881	TRXR1_HUMAN Thioredoxin reductase 1_ cytoplasmic OS=Homo sapiens OX=9606 GN	297,5	1,43
663	P27824	CALX_HUMAN Calnexin OS=Homo sapiens OX=9606 GN=CANX PE=1 SV=2	675,42	1,43
664	P09496	CLCA_HUMAN Clathrin light chain A OS=Homo sapiens OX=9606 GN=CLTA PE=1 SV=1	287	1,43
665	P00367	DHE3_HUMAN Glutamate dehydrogenase 1_ mitochondrial OS=Homo sapiens OX=96	303,48	1,43
666	P61254	RL26_HUMAN 60S ribosomal protein L26 OS=Homo sapiens OX=9606 GN=RPL26 PE=1	498,12	1,43
667	Q96AE4	FUBP1_HUMAN Far upstream element-binding protein 1 OS=Homo sapiens OX=9606	165,12	1,43
668	P34932	HSP74_HUMAN Heat shock 70 kDa protein 4 OS=Homo sapiens OX=9606 GN=HSPA4 F	221,88	1,42
669	Q13838	DX39B_HUMAN Spliceosome RNA helicase DDX39B OS=Homo sapiens OX=9606 GN=D	521,7	1,42
670	P13667	PDIA4_HUMAN Protein disulfide-isomerase A4 OS=Homo sapiens OX=9606 GN=PDIA4	839,15	1,42

671	O75874	IDHC_HUMAN Isocitrate dehydrogenase [NADP] cytoplasmic OS=Homo sapiens OX=96	254,82	1,42
672	P52272	HNRPM_HUMAN Heterogeneous nuclear ribonucleoprotein M OS=Homo sapiens OX=	1136,39	1,42
673	P61586	RHOA_HUMAN Transforming protein RhoA OS=Homo sapiens OX=9606 GN=RHOA PE=	2430,91	1,42
674	P10314	1A32_HUMAN HLA class I histocompatibility antigen_ A-32 alpha chain OS=Homo sapi	763,93	1,42
675	O14818	PSA7_HUMAN Proteasome subunit alpha type-7 OS=Homo sapiens OX=9606 GN=PSM	727,8	1,42
676	P18085	ARF4_HUMAN ADP-ribosylation factor 4 OS=Homo sapiens OX=9606 GN=ARF4 PE=1 S	2053,12	1,42
677	Q02878	RL6_HUMAN 60S ribosomal protein L6 OS=Homo sapiens OX=9606 GN=RPL6 PE=1 SV=	672,22	1,42
678	P42677	RS27_HUMAN 40S ribosomal protein S27 OS=Homo sapiens OX=9606 GN=RPS27 PE=1	3518,56	1,42
679	P09972	ALDOC_HUMAN Fructose-bisphosphate aldolase C OS=Homo sapiens OX=9606 GN=AL	751,33	1,42
680	Q13642	FHL1_HUMAN Four and a half LIM domains protein 1 OS=Homo sapiens OX=9606 GN=	512,14	1,42
681	P12004	PCNA_HUMAN Proliferating cell nuclear antigen OS=Homo sapiens OX=9606 GN=PCN	353,52	1,42
682	P08195	4F2_HUMAN 4F2 cell-surface antigen heavy chain OS=Homo sapiens OX=9606 GN=SL	291,19	1,42
683	P09525	ANXA4_HUMAN Annexin A4 OS=Homo sapiens OX=9606 GN=ANXA4 PE=1 SV=4	7180,95	1,42
684	P46782	RS5_HUMAN 40S ribosomal protein S5 OS=Homo sapiens OX=9606 GN=RPS5 PE=1 SV	352,43	1,42
685	Q14195	DPYL3_HUMAN Dihydropyrimidinase-related protein 3 OS=Homo sapiens OX=9606 G	233,23	1,42
686	P55854	SUMO3_HUMAN Small ubiquitin-related modifier 3 OS=Homo sapiens OX=9606 GN=S	2438,91	1,42
687	P13010	XRCC5_HUMAN X-ray repair cross-complementing protein 5 OS=Homo sapiens OX=96	487,87	1,40
688	Q58FG1	HS904_HUMAN Putative heat shock protein HSP 90-alpha A4 OS=Homo sapiens OX=9	1055,22	1,40
689	P57721	PCBP3_HUMAN Poly(rC)-binding protein 3 OS=Homo sapiens OX=9606 GN=PCBP3 PE=	1098,04	1,40
690	Q9Y281	COF2_HUMAN Cofilin-2 OS=Homo sapiens OX=9606 GN=CFL2 PE=1 SV=1	3185,17	1,40
691	P35268	RL22_HUMAN 60S ribosomal protein L22 OS=Homo sapiens OX=9606 GN=RPL22 PE=1	3484,59	1,40
692	P07196	NFL_HUMAN Neurofilament light polypeptide OS=Homo sapiens OX=9606 GN=NEFL P	418,02	1,40
693	P23246	SFPQ_HUMAN Splicing factor_ proline- and glutamine-rich OS=Homo sapiens OX=960	1041,6	1,40
694	P62424	RL7A_HUMAN 60S ribosomal protein L7a OS=Homo sapiens OX=9606 GN=RPL7A PE=1	1403,41	1,40
695	Q8NFI4	F10A5_HUMAN Putative protein FAM10A5 OS=Homo sapiens OX=9606 GN=ST13P5 P	1168,09	1,40
696	P62879	GBB2_HUMAN Guanine nucleotide-binding protein G(I)/G(S)/G(T) subunit beta-2 OS=	575,85	1,40
697	O43143	DHX15_HUMAN Pre-mRNA-splicing factor ATP-dependent RNA helicase DHX15 OS=H	281,38	1,40
698	P49207	RL34_HUMAN 60S ribosomal protein L34 OS=Homo sapiens OX=9606 GN=RPL34 PE=1	364,02	1,40
699	P35237	SPB6_HUMAN Serpin B6 OS=Homo sapiens OX=9606 GN=SERPINB6 PE=1 SV=3	172,97	1,40
700	P19087	GNAT2_HUMAN Guanine nucleotide-binding protein G(t) subunit alpha-2 OS=Homo s	337,68	1,40
701	P62140	PP1B_HUMAN Serine/threonine-protein phosphatase PP1-beta catalytic subunit OS=H	217,77	1,40

702	P24534	EF1B_HUMAN Elongation factor 1-beta OS=Homo sapiens OX=9606 GN=EEF1B2 PE=1	667,75	1,39
703	P50395	GDIB_HUMAN Rab GDP dissociation inhibitor beta OS=Homo sapiens OX=9606 GN=GI	1259,66	1,39
704	P15153	RAC2_HUMAN Ras-related C3 botulinum toxin substrate 2 OS=Homo sapiens OX=9606	867,81	1,39
705	P62913	RL11_HUMAN 60S ribosomal protein L11 OS=Homo sapiens OX=9606 GN=RPL11 PE=1	2373,91	1,39
706	P30447	1A23_HUMAN HLA class I histocompatibility antigen_ A-23 alpha chain OS=Homo sapi	650,66	1,39
707	Q01469	FABP5_HUMAN Fatty acid-binding protein_ epidermal OS=Homo sapiens OX=9606 GN	1416,3	1,39
708	P09382	LEG1_HUMAN Galectin-1 OS=Homo sapiens OX=9606 GN=LGALS1 PE=1 SV=2	3367,94	1,39
709	P62873	GBB1_HUMAN Guanine nucleotide-binding protein G(I)/G(S)/G(T) subunit beta-1 OS=I	755,67	1,39
710	P23528	COF1_HUMAN Cofilin-1 OS=Homo sapiens OX=9606 GN=CFL1 PE=1 SV=3	5953,2	1,39
711	O60701	UGDH_HUMAN UDP-glucose 6-dehydrogenase OS=Homo sapiens OX=9606 GN=UGDH	680,95	1,39
712	P05534	1A24_HUMAN HLA class I histocompatibility antigen_ A-24 alpha chain OS=Homo sapi	650,66	1,39
713	Q15185	TEBP_HUMAN Prostaglandin E synthase 3 OS=Homo sapiens OX=9606 GN=PTGES3 PE	910,52	1,39
714	P61158	ARP3_HUMAN Actin-related protein 3 OS=Homo sapiens OX=9606 GN=ACTR3 PE=1 S\	305,44	1,39
715	E9PAV3	NACAM_HUMAN Nascent polypeptide-associated complex subunit alpha_ muscle-spe	438,09	1,39
716	P78417	GSTO1_HUMAN Glutathione S-transferase omega-1 OS=Homo sapiens OX=9606 GN=(	504,84	1,39
717	Q4VXU2	PAP1L_HUMAN Polyadenylate-binding protein 1-like OS=Homo sapiens OX=9606 GN=	154,68	1,39
718	O75531	BAF_HUMAN Barrier-to-autointegration factor OS=Homo sapiens OX=9606 GN=BANF	406,73	1,39
719	P40227	TCPZ_HUMAN T-complex protein 1 subunit zeta OS=Homo sapiens OX=9606 GN=CCT6	335,69	1,39
720	O43852	CALU_HUMAN Calumenin OS=Homo sapiens OX=9606 GN=CALU PE=1 SV=2	322,38	1,39
721	Q9UBI6	GBG12_HUMAN Guanine nucleotide-binding protein G(I)/G(S)/G(O) subunit gamma-1	1012,87	1,39
722	P22626	ROA2_HUMAN Heterogeneous nuclear ribonucleoproteins A2/B1 OS=Homo sapiens C	741,07	1,38
723	P84085	ARF5_HUMAN ADP-ribosylation factor 5 OS=Homo sapiens OX=9606 GN=ARF5 PE=1 S	1508,17	1,38
724	P48047	ATPO_HUMAN ATP synthase subunit O_ mitochondrial OS=Homo sapiens OX=9606 GI	1059,29	1,38
725	P17987	TCPA_HUMAN T-complex protein 1 subunit alpha OS=Homo sapiens OX=9606 GN=TCI	420,47	1,38
726	P61160	ARP2_HUMAN Actin-related protein 2 OS=Homo sapiens OX=9606 GN=ACTR2 PE=1 S\	738,1	1,38
727	P55795	HNRH2_HUMAN Heterogeneous nuclear ribonucleoprotein H2 OS=Homo sapiens OX=	424,35	1,38
728	P26599	PTBP1_HUMAN Polypyrimidine tract-binding protein 1 OS=Homo sapiens OX=9606 GN	361,93	1,38
729	Q16658	FSCN1_HUMAN Fascin OS=Homo sapiens OX=9606 GN=FSCN1 PE=1 SV=3	640,54	1,38
730	P60981	DEST_HUMAN Destrin OS=Homo sapiens OX=9606 GN=DSTN PE=1 SV=3	1794,09	1,38
731	P67936	TPM4_HUMAN Tropomyosin alpha-4 chain OS=Homo sapiens OX=9606 GN=TPM4 PE=	849,23	1,38
732	Q6NVV1	R13P3_HUMAN Putative 60S ribosomal protein L13a protein RPL13AP3 OS=Homo sap	2008,29	1,38

733	P08134	RHOC_HUMAN Rho-related GTP-binding protein RhoC OS=Homo sapiens OX=9606 GN	2350,19	1,38
734	P09211	GSTP1_HUMAN Glutathione S-transferase P OS=Homo sapiens OX=9606 GN=GSTP1 PI	3664,45	1,38
735	Q13263	TIF1B_HUMAN Transcription intermediary factor 1-beta OS=Homo sapiens OX=9606 G	531,73	1,38
736	P61978	HNRPK_HUMAN Heterogeneous nuclear ribonucleoprotein K OS=Homo sapiens OX=96	1841,92	1,38
737	Q70Z35	PREX2_HUMAN Phosphatidylinositol 3_4_5-trisphosphate-dependent Rac exchanger 2	335,35	1,38
738	P04080	CYTB_HUMAN Cystatin-B OS=Homo sapiens OX=9606 GN=CSTB PE=1 SV=2	2967,8	1,38
739	P63000	RAC1_HUMAN Ras-related C3 botulinum toxin substrate 1 OS=Homo sapiens OX=9606	1152,16	1,38
740	P52943	CRIP2_HUMAN Cysteine-rich protein 2 OS=Homo sapiens OX=9606 GN=CRIP2 PE=1 SV	669,43	1,38
741	Q9UBT6	POLK_HUMAN DNA polymerase kappa OS=Homo sapiens OX=9606 GN=POLK PE=1 SV=	325,47	1,38
742	P22314	UBA1_HUMAN Ubiquitin-like modifier-activating enzyme 1 OS=Homo sapiens OX=960	500,91	1,36
743	P40939	ECHA_HUMAN Trifunctional enzyme subunit alpha_ mitochondrial OS=Homo sapiens	591,83	1,36
744	Q16181	SEPT7_HUMAN Septin-7 OS=Homo sapiens OX=9606 GN=SEPT7 PE=1 SV=2	492,76	1,36
745	O94925	GLSK_HUMAN Glutaminase kidney isoform_ mitochondrial OS=Homo sapiens OX=960	767,34	1,36
746	P31948	STIP1_HUMAN Stress-induced-phosphoprotein 1 OS=Homo sapiens OX=9606 GN=STIP	925,06	1,36
747	P62937	PPIA_HUMAN Peptidyl-prolyl cis-trans isomerase A OS=Homo sapiens OX=9606 GN=PI	2735,37	1,36
748	Q99497	PARK7_HUMAN Protein/nucleic acid deglycase DJ-1 OS=Homo sapiens OX=9606 GN=P	3577,66	1,36
749	P05023	AT1A1_HUMAN Sodium/potassium-transporting ATPase subunit alpha-1 OS=Homo sa	912,92	1,36
750	P14625	ENPL_HUMAN Endoplasmin OS=Homo sapiens OX=9606 GN=HSP90B1 PE=1 SV=1	4478,02	1,36
751	P46776	RL27A_HUMAN 60S ribosomal protein L27a OS=Homo sapiens OX=9606 GN=RPL27A P	1708,91	1,36
752	Q15233	NONO_HUMAN Non-POU domain-containing octamer-binding protein OS=Homo sapie	676,14	1,36
753	P50502	F10A1_HUMAN Hsc70-interacting protein OS=Homo sapiens OX=9606 GN=ST13 PE=1	1836,11	1,36
754	P13797	PLST_HUMAN Plastin-3 OS=Homo sapiens OX=9606 GN=PLS3 PE=1 SV=4	374,53	1,36
755	P36578	RL4_HUMAN 60S ribosomal protein L4 OS=Homo sapiens OX=9606 GN=RPL4 PE=1 SV=	950,7	1,36
756	P61313	RL15_HUMAN 60S ribosomal protein L15 OS=Homo sapiens OX=9606 GN=RPL15 PE=1	1305,63	1,36
757	Q9Y3U8	RL36_HUMAN 60S ribosomal protein L36 OS=Homo sapiens OX=9606 GN=RPL36 PE=1	1679,66	1,36
758	O60343	TBCD4_HUMAN TBC1 domain family member 4 OS=Homo sapiens OX=9606 GN=TBC1	316,99	1,36
759	P49755	TMEDA_HUMAN Transmembrane emp24 domain-containing protein 10 OS=Homo sap	478,44	1,36
760	Q9Y266	NUDC_HUMAN Nuclear migration protein nudC OS=Homo sapiens OX=9606 GN=NUD	468,72	1,36
761	P18669	PGAM1_HUMAN Phosphoglycerate mutase 1 OS=Homo sapiens OX=9606 GN=PGAM1	702	1,36
762	P61020	RAB5B_HUMAN Ras-related protein Rab-5B OS=Homo sapiens OX=9606 GN=RAB5B PI	1049,04	1,36
763	P62857	RS28_HUMAN 40S ribosomal protein S28 OS=Homo sapiens OX=9606 GN=RPS28 PE=1	1107,03	1,36

764	P51149	RAB7A_HUMAN Ras-related protein Rab-7a OS=Homo sapiens OX=9606 GN=RAB7A PI	2149,97	1,35
765	P62917	RL8_HUMAN 60S ribosomal protein L8 OS=Homo sapiens OX=9606 GN=RPL8 PE=1 SV=	2507,74	1,35
766	P35609	ACTN2_HUMAN Alpha-actinin-2 OS=Homo sapiens OX=9606 GN=ACTN2 PE=1 SV=1	748,94	1,35
767	P05387	RLA2_HUMAN 60S acidic ribosomal protein P2 OS=Homo sapiens OX=9606 GN=RPLP2	4400,03	1,35
768	Q93045	STMN2_HUMAN Stathmin-2 OS=Homo sapiens OX=9606 GN=STMN2 PE=1 SV=3	991,6	1,35
769	Q00839	HNRPU_HUMAN Heterogeneous nuclear ribonucleoprotein U OS=Homo sapiens OX=9	1268,92	1,35
770	P04075	ALDOA_HUMAN Fructose-bisphosphate aldolase A OS=Homo sapiens OX=9606 GN=AI	4683,95	1,35
771	P55084	ECHB_HUMAN Trifunctional enzyme subunit beta_ mitochondrial OS=Homo sapiens C	325,29	1,35
772	Q12797	ASPH_HUMAN Aspartyl/asparaginyl beta-hydroxylase OS=Homo sapiens OX=9606 GN	167,83	1,35
773	P27816	MAP4_HUMAN Microtubule-associated protein 4 OS=Homo sapiens OX=9606 GN=MA	224,31	1,35
774	P06576	ATPB_HUMAN ATP synthase subunit beta_ mitochondrial OS=Homo sapiens OX=9606	4118,66	1,34
775	O95758	PTBP3_HUMAN Polypyrimidine tract-binding protein 3 OS=Homo sapiens OX=9606 GN	186,76	1,34
776	P52209	6PGD_HUMAN 6-phosphogluconate dehydrogenase_ decarboxylating OS=Homo sapie	619,36	1,34
777	P53999	TCP4_HUMAN Activated RNA polymerase II transcriptional coactivator p15 OS=Homo	1918,53	1,34
778	P23396	RS3_HUMAN 40S ribosomal protein S3 OS=Homo sapiens OX=9606 GN=RPS3 PE=1 SV=	2628,66	1,34
779	P37802	TAGL2_HUMAN Transgelin-2 OS=Homo sapiens OX=9606 GN=TAGLN2 PE=1 SV=3	6142,84	1,34
780	P17661	DESM_HUMAN Desmin OS=Homo sapiens OX=9606 GN=DES PE=1 SV=3	1180,49	1,34
781	O60506	HNRPQ_HUMAN Heterogeneous nuclear ribonucleoprotein Q OS=Homo sapiens OX=9	433,75	1,34
782	P62280	RS11_HUMAN 40S ribosomal protein S11 OS=Homo sapiens OX=9606 GN=RPS11 PE=1	1807,43	1,34
783	P39687	AN32A_HUMAN Acidic leucine-rich nuclear phosphoprotein 32 family member A OS=I	494,77	1,34
784	Q07020	RL18_HUMAN 60S ribosomal protein L18 OS=Homo sapiens OX=9606 GN=RPL18 PE=1	1781,41	1,34
785	P32119	PRDX2_HUMAN Peroxiredoxin-2 OS=Homo sapiens OX=9606 GN=PRDX2 PE=1 SV=5	1366,99	1,34
786	P08758	ANXA5_HUMAN Annexin A5 OS=Homo sapiens OX=9606 GN=ANXA5 PE=1 SV=2	3275,42	1,34
787	P50995	ANX11_HUMAN Annexin A11 OS=Homo sapiens OX=9606 GN=ANXA11 PE=1 SV=1	225,86	1,34
788	P55786	PSA_HUMAN Puromycin-sensitive aminopeptidase OS=Homo sapiens OX=9606 GN=NI	402,73	1,34
789	P63244	RACK1_HUMAN Receptor of activated protein C kinase 1 OS=Homo sapiens OX=9606 (	2559,24	1,34
790	P09960	LKHA4_HUMAN Leukotriene A-4 hydrolase OS=Homo sapiens OX=9606 GN=LTA4H PE=	221,89	1,34
791	P32970	CD70_HUMAN CD70 antigen OS=Homo sapiens OX=9606 GN=CD70 PE=1 SV=2	555,55	1,34
792	P13073	COX41_HUMAN Cytochrome c oxidase subunit 4 isoform 1_ mitochondrial OS=Homo s	399,41	1,34
793	P60174	TPIS_HUMAN Triosephosphate isomerase OS=Homo sapiens OX=9606 GN=TPI1 PE=1 S	7682,34	1,32
794	P41091	IF2G_HUMAN Eukaryotic translation initiation factor 2 subunit 3 OS=Homo sapiens OX	372,4	1,32

795	Q14847	LASP1_HUMAN LIM and SH3 domain protein 1 OS=Homo sapiens OX=9606 GN=LASP1	994,22	1,32
796	Q29960	1C16_HUMAN HLA class I histocompatibility antigen_ Cw-16 alpha chain OS=Homo sa	611,33	1,32
797	Q92598	HS105_HUMAN Heat shock protein 105 kDa OS=Homo sapiens OX=9606 GN=HSPH1 P	280,58	1,32
798	P45880	VDAC2_HUMAN Voltage-dependent anion-selective channel protein 2 OS=Homo sapie	934,94	1,32
799	Q16555	DPYL2_HUMAN Dihydropyrimidinase-related protein 2 OS=Homo sapiens OX=9606 GI	445,87	1,32
800	P60842	IF4A1_HUMAN Eukaryotic initiation factor 4A-I OS=Homo sapiens OX=9606 GN=EIF4A	2426,99	1,32
801	Q92841	DDX17_HUMAN Probable ATP-dependent RNA helicase DDX17 OS=Homo sapiens OX=	1091,83	1,32
802	P16949	STMN1_HUMAN Stathmin OS=Homo sapiens OX=9606 GN=STMN1 PE=1 SV=3	2136,5	1,32
803	Q71UM5	RS27L_HUMAN 40S ribosomal protein S27-like OS=Homo sapiens OX=9606 GN=RPS27	3416,42	1,32
804	Q8TAA3	PSMA8_HUMAN Proteasome subunit alpha-type 8 OS=Homo sapiens OX=9606 GN=PS	233,3	1,32
805	Q15019	SEPT2_HUMAN Septin-2 OS=Homo sapiens OX=9606 GN=SEPT2 PE=1 SV=1	572,95	1,32
806	P47756	CAPZB_HUMAN F-actin-capping protein subunit beta OS=Homo sapiens OX=9606 GN=	361,1	1,32
807	P32019	I5P2_HUMAN Type II inositol 1_4_5-trisphosphate 5-phosphatase OS=Homo sapiens (	278,85	1,32
808	Q07065	CKAP4_HUMAN Cytoskeleton-associated protein 4 OS=Homo sapiens OX=9606 GN=Ck	397,39	1,32
809	P67809	YBOX1_HUMAN Nuclease-sensitive element-binding protein 1 OS=Homo sapiens OX=(	1007,93	1,32
810	Q03252	LMNB2_HUMAN Lamin-B2 OS=Homo sapiens OX=9606 GN=LMNB2 PE=1 SV=4	282,15	1,32
811	P37837	TALDO_HUMAN Transaldolase OS=Homo sapiens OX=9606 GN=TALDO1 PE=1 SV=2	2340,42	1,31
812	P34897	GLYM_HUMAN Serine hydroxymethyltransferase_ mitochondrial OS=Homo sapiens O	534,01	1,31
813	P06748	NPM_HUMAN Nucleophosmin OS=Homo sapiens OX=9606 GN=NPM1 PE=1 SV=2	3924,62	1,31
814	P62826	RAN_HUMAN GTP-binding nuclear protein Ran OS=Homo sapiens OX=9606 GN=RAN F	4538,19	1,31
815	Q9UQ80	PA2G4_HUMAN Proliferation-associated protein 2G4 OS=Homo sapiens OX=9606 GN=	1542,81	1,31
816	P18621	RL17_HUMAN 60S ribosomal protein L17 OS=Homo sapiens OX=9606 GN=RPL17 PE=1	1836,62	1,31
817	P30041	PRDX6_HUMAN Peroxiredoxin-6 OS=Homo sapiens OX=9606 GN=PRDX6 PE=1 SV=3	1669,2	1,31
818	P55072	TERA_HUMAN Transitional endoplasmic reticulum ATPase OS=Homo sapiens OX=960(	4673,64	1,31
819	Q8IZP2	ST134_HUMAN Putative protein FAM10A4 OS=Homo sapiens OX=9606 GN=ST13P4 PE	1818,59	1,31
820	P25705	ATPA_HUMAN ATP synthase subunit alpha_ mitochondrial OS=Homo sapiens OX=960	3226,52	1,31
821	Q99623	PHB2_HUMAN Prohibitin-2 OS=Homo sapiens OX=9606 GN=PHB2 PE=1 SV=2	534,52	1,31
822	P36542	ATPG_HUMAN ATP synthase subunit gamma_ mitochondrial OS=Homo sapiens OX=9(	610,43	1,31
823	P68036	UB2L3_HUMAN Ubiquitin-conjugating enzyme E2 L3 OS=Homo sapiens OX=9606 GN=	854,58	1,31
824	P46940	IQGA1_HUMAN Ras GTPase-activating-like protein IQGAP1 OS=Homo sapiens OX=960	379,55	1,31
825	P08133	ANXA6_HUMAN Annexin A6 OS=Homo sapiens OX=9606 GN=ANXA6 PE=1 SV=3	323,09	1,31

826	Q14697	GANAB_HUMAN Neutral alpha-glucosidase AB OS=Homo sapiens OX=9606 GN=GANA	687,91	1,31
827	P07951	TPM2_HUMAN Tropomyosin beta chain OS=Homo sapiens OX=9606 GN=TPM2 PE=1 S	848,84	1,31
828	O75828	CBR3_HUMAN Carbonyl reductase [NADPH] 3 OS=Homo sapiens OX=9606 GN=CBR3 P	465,07	1,31
829	P62910	RL32_HUMAN 60S ribosomal protein L32 OS=Homo sapiens OX=9606 GN=RPL32 PE=1	780,88	1,31
830	P47914	RL29_HUMAN 60S ribosomal protein L29 OS=Homo sapiens OX=9606 GN=RPL29 PE=1	455,33	1,31
831	P00742	FA10_HUMAN Coagulation factor X OS=Homo sapiens OX=9606 GN=F10 PE=1 SV=2	247,8	1,31
832	P15880	RS2_HUMAN 40S ribosomal protein S2 OS=Homo sapiens OX=9606 GN=RPS2 PE=1 SV=	1562,31	1,30
833	P07900	HS90A_HUMAN Heat shock protein HSP 90-alpha OS=Homo sapiens OX=9606 GN=HSP	11750,56	1,30
834	Q14240	IF4A2_HUMAN Eukaryotic initiation factor 4A-II OS=Homo sapiens OX=9606 GN=EIF4A	1844,78	1,30
835	P07237	PDIA1_HUMAN Protein disulfide-isomerase OS=Homo sapiens OX=9606 GN=P4HB PE=	2226,74	1,30
836	P0DMV9	HS71B_HUMAN Heat shock 70 kDa protein 1B OS=Homo sapiens OX=9606 GN=HSPA1	5269,26	1,30
837	P0DMV8	HS71A_HUMAN Heat shock 70 kDa protein 1A OS=Homo sapiens OX=9606 GN=HSPA1	5269,26	1,30
838	P06753	TPM3_HUMAN Tropomyosin alpha-3 chain OS=Homo sapiens OX=9606 GN=TPM3 PE=	796,56	1,30
839	Q00325	MPCP_HUMAN Phosphate carrier protein_ mitochondrial OS=Homo sapiens OX=9606	1481,94	1,30
840	P31150	GDIA_HUMAN Rab GDP dissociation inhibitor alpha OS=Homo sapiens OX=9606 GN=G	534,86	1,30
841	P15311	EZRI_HUMAN Ezrin OS=Homo sapiens OX=9606 GN=EZR PE=1 SV=4	2481,99	1,30
842	P40429	RL13A_HUMAN 60S ribosomal protein L13a OS=Homo sapiens OX=9606 GN=RPL13A P	3199,99	1,30
843	Q08043	ACTN3_HUMAN Alpha-actinin-3 OS=Homo sapiens OX=9606 GN=ACTN3 PE=1 SV=2	634,47	1,30
844	P61353	RL27_HUMAN 60S ribosomal protein L27 OS=Homo sapiens OX=9606 GN=RPL27 PE=1	784,85	1,30
845	P18206	VINC_HUMAN Vinculin OS=Homo sapiens OX=9606 GN=VCL PE=1 SV=4	1041,2	1,30
846	P62899	RL31_HUMAN 60S ribosomal protein L31 OS=Homo sapiens OX=9606 GN=RPL31 PE=1	993,28	1,30
847	Q5T655	CFA58_HUMAN Cilia- and flagella-associated protein 58 OS=Homo sapiens OX=9606 G	317,58	1,30
848	Q96DA2	RB39B_HUMAN Ras-related protein Rab-39B OS=Homo sapiens OX=9606 GN=RAB39B	1095,81	1,30
849	P62263	RS14_HUMAN 40S ribosomal protein S14 OS=Homo sapiens OX=9606 GN=RPS14 PE=1	1415,93	1,30
850	P30455	1A36_HUMAN HLA class I histocompatibility antigen_ A-36 alpha chain OS=Homo sapi	544,17	1,30
851	P22234	PUR6_HUMAN Multifunctional protein ADE2 OS=Homo sapiens OX=9606 GN=PAICS P	283,26	1,30
852	O14980	XPO1_HUMAN Exportin-1 OS=Homo sapiens OX=9606 GN=XPO1 PE=1 SV=1	281,51	1,30
853	A5PKW4	PSD1_HUMAN PH and SEC7 domain-containing protein 1 OS=Homo sapiens OX=9606	282,45	1,30
854	P30443	1A01_HUMAN HLA class I histocompatibility antigen_ A-1 alpha chain OS=Homo sapie	544,17	1,30
855	P54577	SYYC_HUMAN Tyrosine--tRNA ligase_ cytoplasmic OS=Homo sapiens OX=9606 GN=YA	454,8	0,70
856	Q6ZU80	CE128_HUMAN Centrosomal protein of 128 kDa OS=Homo sapiens OX=9606 GN=CEP1	71,53	0,70

857	P02768	ALBU_HUMAN Serum albumin OS=Homo sapiens OX=9606 GN=ALB PE=1 SV=2	3368,19	0,70
858	P29992	GNA11_HUMAN Guanine nucleotide-binding protein subunit alpha-11 OS=Homo sapi	533,12	0,69
859	Q9Y2J8	PADI2_HUMAN Protein-arginine deiminase type-2 OS=Homo sapiens OX=9606 GN=PA	114,95	0,65
860	P04004	VTNC_HUMAN Vitronectin OS=Homo sapiens OX=9606 GN=VTN PE=1 SV=1	253,9	0,65
861	Q04695	K1C17_HUMAN Keratin_ type I cytoskeletal 17 OS=Homo sapiens OX=9606 GN=KRT17	947,15	0,65
862	P19012	K1C15_HUMAN Keratin_ type I cytoskeletal 15 OS=Homo sapiens OX=9606 GN=KRT15	881,79	0,65
863	Q14532	K1H2_HUMAN Keratin_ type I cuticular Ha2 OS=Homo sapiens OX=9606 GN=KRT32 PE	490,45	0,64
864	Q8N371	KDM8_HUMAN JmjC domain-containing protein 5 OS=Homo sapiens OX=9606 GN=KD	308,21	0,64
865	P69891	HBG1_HUMAN Hemoglobin subunit gamma-1 OS=Homo sapiens OX=9606 GN=HBG1 I	1173,15	0,64
866	O76014	KRT37_HUMAN Keratin_ type I cuticular Ha7 OS=Homo sapiens OX=9606 GN=KRT37 P	456,85	0,64
867	Q6N069	NAA16_HUMAN N-alpha-acetyltransferase 16_ NatA auxiliary subunit OS=Homo sapie	330,45	0,63
868	P06732	KCRM_HUMAN Creatine kinase M-type OS=Homo sapiens OX=9606 GN=CKM PE=1 SV	567,5	0,63
869	O76015	KRT38_HUMAN Keratin_ type I cuticular Ha8 OS=Homo sapiens OX=9606 GN=KRT38 P	456,85	0,63
870	O76013	KRT36_HUMAN Keratin_ type I cuticular Ha6 OS=Homo sapiens OX=9606 GN=KRT36 P	516,67	0,63
871	Q99456	K1C12_HUMAN Keratin_ type I cytoskeletal 12 OS=Homo sapiens OX=9606 GN=KRT12	353,6	0,63
872	P69892	HBG2_HUMAN Hemoglobin subunit gamma-2 OS=Homo sapiens OX=9606 GN=HBG2 I	1173,15	0,63
873	P02100	HBE_HUMAN Hemoglobin subunit epsilon OS=Homo sapiens OX=9606 GN=HBE1 PE=1	1173,15	0,62
874	O95155	UBE4B_HUMAN Ubiquitin conjugation factor E4 B OS=Homo sapiens OX=9606 GN=UB	279,84	0,62
875	P04259	K2C6B_HUMAN Keratin_ type II cytoskeletal 6B OS=Homo sapiens OX=9606 GN=KRT6I	1306,79	0,62
876	Q7Z794	K2C1B_HUMAN Keratin_ type II cytoskeletal 1b OS=Homo sapiens OX=9606 GN=KRT7	921,11	0,62
877	Q2M2I5	K1C24_HUMAN Keratin_ type I cytoskeletal 24 OS=Homo sapiens OX=9606 GN=KRT24	595,71	0,62
878	Q92764	KRT35_HUMAN Keratin_ type I cuticular Ha5 OS=Homo sapiens OX=9606 GN=KRT35 P	599,04	0,61
879	P48668	K2C6C_HUMAN Keratin_ type II cytoskeletal 6C OS=Homo sapiens OX=9606 GN=KRT6	352,75	0,61
880	P08779	K1C16_HUMAN Keratin_ type I cytoskeletal 16 OS=Homo sapiens OX=9606 GN=KRT16	1331,92	0,61
881	Q5JTH9	RRP12_HUMAN RRP12-like protein OS=Homo sapiens OX=9606 GN=RRP12 PE=1 SV=2	428,82	0,60
882	Q14525	KT33B_HUMAN Keratin_ type I cuticular Ha3-II OS=Homo sapiens OX=9606 GN=KRT33	485,3	0,60
883	P02042	HBD_HUMAN Hemoglobin subunit delta OS=Homo sapiens OX=9606 GN=HBD PE=1 S\	1445,61	0,59
884	P02533	K1C14_HUMAN Keratin_ type I cytoskeletal 14 OS=Homo sapiens OX=9606 GN=KRT14	1283,19	0,59
885	P13646	K1C13_HUMAN Keratin_ type I cytoskeletal 13 OS=Homo sapiens OX=9606 GN=KRT13	654,85	0,59
886	P68871	HBB_HUMAN Hemoglobin subunit beta OS=Homo sapiens OX=9606 GN=HBB PE=1 SV	1465,08	0,59
887	P02538	K2C6A_HUMAN Keratin_ type II cytoskeletal 6A OS=Homo sapiens OX=9606 GN=KRT6	352,75	0,59

888	P19013	K2C4_HUMAN Keratin_ type II cytoskeletal 4 OS=Homo sapiens OX=9606 GN=KRT4 PE	168,74	0,58
889	P02787	TRFE_HUMAN Serotransferrin OS=Homo sapiens OX=9606 GN=TF PE=1 SV=3	606,31	0,58
890	Q15323	K1H1_HUMAN Keratin_ type I cuticular Ha1 OS=Homo sapiens OX=9606 GN=KRT31 PE	498,34	0,58
891	P04264	K2C1_HUMAN Keratin_ type II cytoskeletal 1 OS=Homo sapiens OX=9606 GN=KRT1 PE	7093,49	0,57
892	P38405	GNAL_HUMAN Guanine nucleotide-binding protein G(olf) subunit alpha OS=Homo sap	522,64	0,57
893	P35527	K1C9_HUMAN Keratin_ type I cytoskeletal 9 OS=Homo sapiens OX=9606 GN=KRT9 PE=	2115,94	0,56
894	Q01432	AMPD3_HUMAN AMP deaminase 3 OS=Homo sapiens OX=9606 GN=AMPD3 PE=1 SV=	284,49	0,55
895	P35908	K22E_HUMAN Keratin_ type II cytoskeletal 2 epidermal OS=Homo sapiens OX=9606 G	2299,27	0,55
896	Q9BS26	ERP44_HUMAN Endoplasmic reticulum resident protein 44 OS=Homo sapiens OX=960	308,44	0,54
897	P13645	K1C10_HUMAN Keratin_ type I cytoskeletal 10 OS=Homo sapiens OX=9606 GN=KRT10	3977,56	0,54
898	P02749	APOH_HUMAN Beta-2-glycoprotein 1 OS=Homo sapiens OX=9606 GN=APOH PE=1 SV=	367,34	0,54
899	P69905	HBA_HUMAN Hemoglobin subunit alpha OS=Homo sapiens OX=9606 GN=HBA1 PE=1 S	626,48	0,54
900	Q7Z3Y7	K1C28_HUMAN Keratin_ type I cytoskeletal 28 OS=Homo sapiens OX=9606 GN=KRT28	1232,55	0,54
901	P52292	IMA1_HUMAN Importin subunit alpha-1 OS=Homo sapiens OX=9606 GN=KPNA2 PE=1	285,95	0,54
902	P81605	DCD_HUMAN Dermcidin OS=Homo sapiens OX=9606 GN=DCD PE=1 SV=2	904,64	0,54
903	Q8NDV3	SMC1B_HUMAN Structural maintenance of chromosomes protein 1B OS=Homo sapier	97,04	0,54
904	O60218	AK1BA_HUMAN Aldo-keto reductase family 1 member B10 OS=Homo sapiens OX=960	444,62	0,52
905	Q9GZV4	IF5A2_HUMAN Eukaryotic translation initiation factor 5A-2 OS=Homo sapiens OX=960	346,65	0,52
906	Q9BX84	TRPM6_HUMAN Transient receptor potential cation channel subfamily M member 6 C	423,61	0,52
907	Q9BTC0	DIDO1_HUMAN Death-inducer obliterator 1 OS=Homo sapiens OX=9606 GN=DIDO1 P	306,85	0,52
908	Q7Z3Y9	K1C26_HUMAN Keratin_ type I cytoskeletal 26 OS=Homo sapiens OX=9606 GN=KRT26	336,58	0,52
909	Q6UVJ0	SAS6_HUMAN Spindle assembly abnormal protein 6 homolog OS=Homo sapiens OX=9	295,63	0,51
910	Q6I9Y2	THOC7_HUMAN THO complex subunit 7 homolog OS=Homo sapiens OX=9606 GN=TH	312,27	0,51
911	Q9Y4C1	KDM3A_HUMAN Lysine-specific demethylase 3A OS=Homo sapiens OX=9606 GN=KDN	306,66	0,50
912	P01023	A2MG_HUMAN Alpha-2-macroglobulin OS=Homo sapiens OX=9606 GN=A2M PE=1 SV	402,22	0,50
913	Q92797	SYMPK_HUMAN Symplekin OS=Homo sapiens OX=9606 GN=SYMPK PE=1 SV=2	219,37	0,50
914	Q7Z3Y8	K1C27_HUMAN Keratin_ type I cytoskeletal 27 OS=Homo sapiens OX=9606 GN=KRT27	784,74	0,49
915	O96000	NDUBA_HUMAN NADH dehydrogenase [ubiquinone] 1 beta subcomplex subunit 10 O	185,98	0,49
916	Q7Z3Z0	K1C25_HUMAN Keratin_ type I cytoskeletal 25 OS=Homo sapiens OX=9606 GN=KRT25	784,74	0,49
917	P13647	K2C5_HUMAN Keratin_ type II cytoskeletal 5 OS=Homo sapiens OX=9606 GN=KRT5 PE	331,83	0,47
918	P02788	TRFL_HUMAN Lactotransferrin OS=Homo sapiens OX=9606 GN=LTF PE=1 SV=6	567	0,46

919	P02771	FETA_HUMAN Alpha-fetoprotein OS=Homo sapiens OX=9606 GN=AFP PE=1 SV=1	309,34	0,46
920	Q00534	CDK6_HUMAN Cyclin-dependent kinase 6 OS=Homo sapiens OX=9606 GN=CDK6 PE=1	350,35	0,46
921	P01031	CO5_HUMAN Complement C5 OS=Homo sapiens OX=9606 GN=C5 PE=1 SV=4	296,79	0,45
922	Q8N1G1	REXO1_HUMAN RNA exonuclease 1 homolog OS=Homo sapiens OX=9606 GN=REXO1	238,14	0,45
923	Q9H270	VPS11_HUMAN Vacuolar protein sorting-associated protein 11 homolog OS=Homo sa	274,24	0,44
924	A7E2Y1	MYH7B_HUMAN Myosin-7B OS=Homo sapiens OX=9606 GN=MYH7B PE=1 SV=4	265,23	0,43
925	Q9HC77	CENPJ_HUMAN Centromere protein J OS=Homo sapiens OX=9606 GN=CENPJ PE=1 SV	316,51	0,43
926	Q5XKE5	K2C79_HUMAN Keratin_ type II cytoskeletal 79 OS=Homo sapiens OX=9606 GN=KRT7	291,89	0,42
927	P01024	CO3_HUMAN Complement C3 OS=Homo sapiens OX=9606 GN=C3 PE=1 SV=2	231,34	0,42
928	P02774	VTDB_HUMAN Vitamin D-binding protein OS=Homo sapiens OX=9606 GN=GC PE=1 SV	213,16	0,42
929	Q9Y3B2	EXOS1_HUMAN Exosome complex component CSL4 OS=Homo sapiens OX=9606 GN=E	182,95	0,41
930	P05543	THBG_HUMAN Thyroxine-binding globulin OS=Homo sapiens OX=9606 GN=SERPINA7	297,73	0,41
931	Q6VMQ6	MCAF1_HUMAN Activating transcription factor 7-interacting protein 1 OS=Homo sapi	408,43	0,41
932	P20742	PZP_HUMAN Pregnancy zone protein OS=Homo sapiens OX=9606 GN=PZP PE=1 SV=4	332,85	0,41
933	Q86TB3	ALPK2_HUMAN Alpha-protein kinase 2 OS=Homo sapiens OX=9606 GN=ALPK2 PE=2 S	300,82	0,39
934	Q06787	FMR1_HUMAN Synaptic functional regulator FMR1 OS=Homo sapiens OX=9606 GN=F	352,59	0,39
935	P78385	KRT83_HUMAN Keratin_ type II cuticular Hb3 OS=Homo sapiens OX=9606 GN=KRT83	12974,83	0,36
936	O43790	KRT86_HUMAN Keratin_ type II cuticular Hb6 OS=Homo sapiens OX=9606 GN=KRT86	11767,57	0,35
937	Q14644	RASA3_HUMAN Ras GTPase-activating protein 3 OS=Homo sapiens OX=9606 GN=RAS	400,48	0,35
938	P19823	ITIH2_HUMAN Inter-alpha-trypsin inhibitor heavy chain H2 OS=Homo sapiens OX=960	358,43	0,34
939	Q9BRP8	PYM1_HUMAN Partner of Y14 and mago OS=Homo sapiens OX=9606 GN=PYM1 PE=1	307,4	0,29
940	Q8IYF3	TEX11_HUMAN Testis-expressed protein 11 OS=Homo sapiens OX=9606 GN=TEX11 PE	359,52	0,29
941	O94892	ZN432_HUMAN Zinc finger protein 432 OS=Homo sapiens OX=9606 GN=ZNF432 PE=1	310,53	0,24
942	P10244	MYBB_HUMAN Myb-related protein B OS=Homo sapiens OX=9606 GN=MYBL2 PE=1 S	394,3	0,15
943	Q14624	ITIH4_HUMAN Inter-alpha-trypsin inhibitor heavy chain H4 OS=Homo sapiens OX=960	612,31	0,1
944	Q14980	NUMA1_HUMAN Nuclear mitotic apparatus protein 1 OS=Homo sapiens OX=9606 GN	123,61	0,1
945	Q9Y6N1	COX11_HUMAN Cytochrome c oxidase assembly protein COX11_ mitochondrial OS=H	213,6	0,1
946	P24522	GA45A_HUMAN Growth arrest and DNA damage-inducible protein GADD45 alpha OS=	254,17	0,1
947	P54198	HIRA_HUMAN Protein HIRA OS=Homo sapiens OX=9606 GN=HIRA PE=1 SV=2	351,55	0,1
948	P12273	PIP_HUMAN Prolactin-inducible protein OS=Homo sapiens OX=9606 GN=PIP PE=1 SV=	2085,32	0,1
949	Q2TB90	HKDC1_HUMAN Putative hexokinase HKDC1 OS=Homo sapiens OX=9606 GN=HKDC1	248,3	0,1

950	Q9Y6K9	NEMO_HUMAN NF-kappa-B essential modulator OS=Homo sapiens OX=9606 GN=IKBK	289,33	0,1
951	Q12768	WASC5_HUMAN WASH complex subunit 5 OS=Homo sapiens OX=9606 GN=WASHC5 F	190,66	0,1
952	Q9Y3B7	RM11_HUMAN 39S ribosomal protein L11_ mitochondrial OS=Homo sapiens OX=9606	280,2	0,1
953	Q17R98	ZN827_HUMAN Zinc finger protein 827 OS=Homo sapiens OX=9606 GN=ZNF827 PE=1	267,26	0,1
954	Q7Z5M8	AB12B_HUMAN Protein ABHD12B OS=Homo sapiens OX=9606 GN=ABHD12B PE=2 SV=	773,78	0,1
955	Q86T90	K1328_HUMAN Protein hinderin OS=Homo sapiens OX=9606 GN=KIAA1328 PE=1 SV=2	219,12	0,1
956	P0C7H8	KRA23_HUMAN Keratin-associated protein 2-3 OS=Homo sapiens OX=9606 GN=KRTAF	6903,6	0,1
957	Q9BYU5	KRA21_HUMAN Keratin-associated protein 2-1 OS=Homo sapiens OX=9606 GN=KRTAF	6903,6	0,1
958	P40938	RFC3_HUMAN Replication factor C subunit 3 OS=Homo sapiens OX=9606 GN=RFC3 PE	284,39	0,1
959	Q8IYB8	SUV3_HUMAN ATP-dependent RNA helicase SUPV3L1_ mitochondrial OS=Homo sapie	363,48	0,1
960	Q9BYT5	KRA22_HUMAN Keratin-associated protein 2-2 OS=Homo sapiens OX=9606 GN=KRTAF	6903,6	0,1
961	O14561	ACPM_HUMAN Acyl carrier protein_ mitochondrial OS=Homo sapiens OX=9606 GN=N	236,68	0,1
962	Q92187	SIA8D_HUMAN CMP-N-acetylneuraminate-poly-alpha-2_8-sialyltransferase OS=Homo	254,19	0,1
963	Q9BYR9	KRA24_HUMAN Keratin-associated protein 2-4 OS=Homo sapiens OX=9606 GN=KRTAF	6903,6	0,1
964	Q9UHR6	ZNHI2_HUMAN Zinc finger HIT domain-containing protein 2 OS=Homo sapiens OX=96	172,45	0,1
965	P01008	ANT3_HUMAN Antithrombin-III OS=Homo sapiens OX=9606 GN=SERPINC1 PE=1 SV=1	294,1	0,1
966	Q53GS7	GLE1_HUMAN Nucleoporin GLE1 OS=Homo sapiens OX=9606 GN=GLE1 PE=1 SV=2	260,65	0,1
967	Q7L523	RRAGA_HUMAN Ras-related GTP-binding protein A OS=Homo sapiens OX=9606 GN=R	911,03	0,1
968	Q02952	AKA12_HUMAN A-kinase anchor protein 12 OS=Homo sapiens OX=9606 GN=AKAP12 I	116,89	0,1
969	P53680	AP2S1_HUMAN AP-2 complex subunit sigma OS=Homo sapiens OX=9606 GN=AP2S1 P	454,42	0,1
970	O14863	ZNT4_HUMAN Zinc transporter 4 OS=Homo sapiens OX=9606 GN=SLC30A4 PE=1 SV=2	261,95	0,1
971	Q9UL25	RAB21_HUMAN Ras-related protein Rab-21 OS=Homo sapiens OX=9606 GN=RAB21 PE	275,87	0,1
972	P10632	CP2C8_HUMAN Cytochrome P450 2C8 OS=Homo sapiens OX=9606 GN=CYP2C8 PE=1 S	226,56	0,1
973	Q9Y2R4	DDX52_HUMAN Probable ATP-dependent RNA helicase DDX52 OS=Homo sapiens OX=	120,8	0,1
974	O95399	UTS2_HUMAN Urotensin-2 OS=Homo sapiens OX=9606 GN=UTS2 PE=1 SV=1	288,18	0,1
975	Q6NY19	KANK3_HUMAN KN motif and ankyrin repeat domain-containing protein 3 OS=Homo s	251,73	0,1
976	O95394	AGM1_HUMAN Phosphoacetylglucosamine mutase OS=Homo sapiens OX=9606 GN=P	251,65	0,1
977	A0A0A0MS15	HV349_HUMAN Immunoglobulin heavy variable 3-49 OS=Homo sapiens OX=9606 GN=	651,78	0,1
978	Q13163	MP2K5_HUMAN Dual specificity mitogen-activated protein kinase kinase 5 OS=Homo	426,76	0,1
979	A6NCN2	KR87P_HUMAN Putative keratin-87 protein OS=Homo sapiens OX=9606 GN=KRT87P F	9276,97	0,1
980	Q9Y5T5	UBP16_HUMAN Ubiquitin carboxyl-terminal hydrolase 16 OS=Homo sapiens OX=9606	268,51	0,1

981	P08697	A2AP_HUMAN Alpha-2-antiplasmin OS=Homo sapiens OX=9606 GN=SERPINF2 PE=1 S	492,04	0,1
982	P18754	RCC1_HUMAN Regulator of chromosome condensation OS=Homo sapiens OX=9606 G	326,22	0,1
983	Q5CZ79	AN20B_HUMAN Ankyrin repeat domain-containing protein 20B OS=Homo sapiens OX	278,03	0,1
984	Q15643	TRIPB_HUMAN Thyroid receptor-interacting protein 11 OS=Homo sapiens OX=9606 G	218,46	0,1
985	Q96DA0	ZG16B_HUMAN Zymogen granule protein 16 homolog B OS=Homo sapiens OX=9606	2335,05	0,1
986	P10915	HPLN1_HUMAN Hyaluronan and proteoglycan link protein 1 OS=Homo sapiens OX=96	223,65	0,1
987	P10911	MCF2_HUMAN Proto-oncogene DBL OS=Homo sapiens OX=9606 GN=MCF2 PE=1 SV=	164,7	0,1
988	Q9BRT9	SLD5_HUMAN DNA replication complex GINS protein SLD5 OS=Homo sapiens OX=960	400,86	0,1
989	P00846	ATP6_HUMAN ATP synthase subunit a OS=Homo sapiens OX=9606 GN=MT-ATP6 PE=1	248,38	0,1
990	Q6PML9	ZNT9_HUMAN Zinc transporter 9 OS=Homo sapiens OX=9606 GN=SLC30A9 PE=1 SV=1	219,88	0,1
991	Q8IUG1	KRA13_HUMAN Keratin-associated protein 1-3 OS=Homo sapiens OX=9606 GN=KRTA	223,02	0,1
992	Q8IUE1	TF2LX_HUMAN Homeobox protein TGIF2LX OS=Homo sapiens OX=9606 GN=TGIF2LX	294,73	0,1
993	Q8IUE0	TF2LY_HUMAN Homeobox protein TGIF2LY OS=Homo sapiens OX=9606 GN=TGIF2LY	294,73	0,1
994	Q13769	THOC5_HUMAN THO complex subunit 5 homolog OS=Homo sapiens OX=9606 GN=TH	232,67	0,1
995	Q8IUC1	KR111_HUMAN Keratin-associated protein 11-1 OS=Homo sapiens OX=9606 GN=KRT	2933,07	0,1
996	Q96JE7	SC16B_HUMAN Protein transport protein Sec16B OS=Homo sapiens OX=9606 GN=SE	270,93	0,1
997	O15247	CLIC2_HUMAN Chloride intracellular channel protein 2 OS=Homo sapiens OX=9606 G	327,31	0,1
998	Q12996	CSTF3_HUMAN Cleavage stimulation factor subunit 3 OS=Homo sapiens OX=9606 GN	264,58	0,1
999	P49747	COMP_HUMAN Cartilage oligomeric matrix protein OS=Homo sapiens OX=9606 GN=C	222,02	0,1
1000	Q96CN5	LRC45_HUMAN Leucine-rich repeat-containing protein 45 OS=Homo sapiens OX=9606	231,85	0,1
1001	A0A1B0GTD5	TEX49_HUMAN Testis-expressed protein 49 OS=Homo sapiens OX=9606 GN=TEX49 P	357,29	0,1
1002	Q9Y262	EIF3L_HUMAN Eukaryotic translation initiation factor 3 subunit L OS=Homo sapiens O	207,56	0,1
1003	Q70EK9	UBP51_HUMAN Ubiquitin carboxyl-terminal hydrolase 51 OS=Homo sapiens OX=9606	277,34	0,1
1004	Q9C075	K1C23_HUMAN Keratin_ type I cytoskeletal 23 OS=Homo sapiens OX=9606 GN=KRT23	447,89	0,1
1005	Q96M91	CFA53_HUMAN Cilia- and flagella-associated protein 53 OS=Homo sapiens OX=9606 G	222,5	0,1
1006	P45985	MP2K4_HUMAN Dual specificity mitogen-activated protein kinase kinase 4 OS=Homo	268,51	0,1
1007	O95267	GRP1_HUMAN RAS guanyl-releasing protein 1 OS=Homo sapiens OX=9606 GN=RASGR	363,83	0,1
1008	P24390	ERD21_HUMAN ER lumen protein-retaining receptor 1 OS=Homo sapiens OX=9606 G	164,2	0,1
1009	P01877	IGHA2_HUMAN Immunoglobulin heavy constant alpha 2 OS=Homo sapiens OX=9606	316,31	0,1
1010	P01876	IGHA1_HUMAN Immunoglobulin heavy constant alpha 1 OS=Homo sapiens OX=9606	518,46	0,1
1011	Q9C029	TRIM7_HUMAN E3 ubiquitin-protein ligase TRIM7 OS=Homo sapiens OX=9606 GN=TR	175,5	0,1

1012	Q86XQ3	CTSR3_HUMAN Cation channel sperm-associated protein 3 OS=Homo sapiens OX=960	434,34	0,1
1013	Q5T2T1	MPP7_HUMAN MAGUK p55 subfamily member 7 OS=Homo sapiens OX=9606 GN=MP	268,51	0,1
1014	P30085	KCY_HUMAN UMP-CMP kinase OS=Homo sapiens OX=9606 GN=CMPK1 PE=1 SV=3	219,82	0,1
1015	P41223	BUD31_HUMAN Protein BUD31 homolog OS=Homo sapiens OX=9606 GN=BUD31 PE=	257,77	0,1
1016	P01834	IGKC_HUMAN Immunoglobulin kappa constant OS=Homo sapiens OX=9606 GN=IGKC	568,39	0,1
1017	Q07627	KRA11_HUMAN Keratin-associated protein 1-1 OS=Homo sapiens OX=9606 GN=KRTA	223,02	0,1
1018	Q86UF4	CC190_HUMAN Coiled-coil domain-containing protein 190 OS=Homo sapiens OX=960	200,86	0,1
1019	P49006	MRP_HUMAN MARCKS-related protein OS=Homo sapiens OX=9606 GN=MARCKSL1 PE	348,04	0,1
1020	A6NI15	MSGN1_HUMAN Mesogenin-1 OS=Homo sapiens OX=9606 GN=MSGN1 PE=3 SV=1	441,92	0,1
1021	Q2T9L4	CO059_HUMAN UPF0583 protein C15orf59 OS=Homo sapiens OX=9606 GN=C15orf59	301,81	0,1
1022	Q6ZRS4	CC129_HUMAN Coiled-coil domain-containing protein 129 OS=Homo sapiens OX=960	356,08	0,1
1023	P33991	MCM4_HUMAN DNA replication licensing factor MCM4 OS=Homo sapiens OX=9606 G	208,96	0,1
1024	P06681	CO2_HUMAN Complement C2 OS=Homo sapiens OX=9606 GN=C2 PE=1 SV=2	174,3	0,1
1025	P60903	S10AA_HUMAN Protein S100-A10 OS=Homo sapiens OX=9606 GN=S100A10 PE=1 SV=	321,6	0,1
1026	Q9H7D0	DOCK5_HUMAN Dedicator of cytokinesis protein 5 OS=Homo sapiens OX=9606 GN=D	194,32	0,1
1027	P33947	ERD22_HUMAN ER lumen protein-retaining receptor 2 OS=Homo sapiens OX=9606 G	199,34	0,1
1028	Q6ZUS5	CC121_HUMAN Coiled-coil domain-containing protein 121 OS=Homo sapiens OX=960	351,01	0,1
1029	P0DOX7	IGK_HUMAN Immunoglobulin kappa light chain OS=Homo sapiens OX=9606 PE=1 SV=	478,7	0,1
1030	P0DOX2	IGA2_HUMAN Immunoglobulin alpha-2 heavy chain OS=Homo sapiens OX=9606 PE=1	316,31	0,1
1031	O95816	BAG2_HUMAN BAG family molecular chaperone regulator 2 OS=Homo sapiens OX=96	183,54	0,1
1032	Q5VZM2	RRAGB_HUMAN Ras-related GTP-binding protein B OS=Homo sapiens OX=9606 GN=R	944,45	0,1
1033	Q9NSB4	KRT82_HUMAN Keratin_ type II cuticular Hb2 OS=Homo sapiens OX=9606 GN=KRT82	5167,44	0,1
1034	Q08050	FOXM1_HUMAN Forkhead box protein M1 OS=Homo sapiens OX=9606 GN=FOXM1 P	170,41	0,1
1035	Q8NHP7	EXD1_HUMAN piRNA biogenesis protein EXD1 OS=Homo sapiens OX=9606 GN=EXD1	322,48	0,1
1036	Q6A162	K1C40_HUMAN Keratin_ type I cytoskeletal 40 OS=Homo sapiens OX=9606 GN=KRT40	2466,55	0,1
1037	Q96HW7	INT4_HUMAN Integrator complex subunit 4 OS=Homo sapiens OX=9606 GN=INTS4 PE	111,65	0,1
1038	Q9BTF0	THUM2_HUMAN THUMP domain-containing protein 2 OS=Homo sapiens OX=9606 G	240,49	0,1
1039	P49913	CAMP_HUMAN Cathelicidin antimicrobial peptide OS=Homo sapiens OX=9606 GN=CA	209,47	0,1
1040	Q9Y3Z3	SAMH1_HUMAN Deoxynucleoside triphosphate triphosphohydrolase SAMHD1 OS=Ho	403,05	0,1
1041	Q9NP55	BPIA1_HUMAN BPI fold-containing family A member 1 OS=Homo sapiens OX=9606 G	413,26	0,1
1042	P28324	ELK4_HUMAN ETS domain-containing protein Elk-4 OS=Homo sapiens OX=9606 GN=E	222,5	0,1

1043	Q68DU8	KCD16_HUMAN BTB/POZ domain-containing protein KCTD16 OS=Homo sapiens OX=9	221,12	0,1
1044	Q96RF0	SNX18_HUMAN Sorting nexin-18 OS=Homo sapiens OX=9606 GN=SNX18 PE=1 SV=2	222,5	0,1
1045	Q8TDL5	BPIB1_HUMAN BPI fold-containing family B member 1 OS=Homo sapiens OX=9606 GN	1257,25	0,1
1046	O75915	PRAF3_HUMAN PRA1 family protein 3 OS=Homo sapiens OX=9606 GN=ARL6IP5 PE=1	201,46	0,1
1047	Q9H6R3	ACSS3_HUMAN Acyl-CoA synthetase short-chain family member 3_ mitochondrial OS=	250,77	0,1
1048	P59047	NALP5_HUMAN NACHT_ LRR and PYD domains-containing protein 5 OS=Homo sapien	280,1	0,1
1049	O43174	CP26A_HUMAN Cytochrome P450 26A1 OS=Homo sapiens OX=9606 GN=CYP26A1 PE=	235,25	0,1
1050	P33260	CP2CI_HUMAN Cytochrome P450 2C18 OS=Homo sapiens OX=9606 GN=CYP2C18 PE=	233,45	0,1
1051	Q16548	B2LA1_HUMAN Bcl-2-related protein A1 OS=Homo sapiens OX=9606 GN=BCL2A1 PE=	451,5	0,1
1052	A2RTY3	HEAT9_HUMAN Protein HEATR9 OS=Homo sapiens OX=9606 GN=HEATR9 PE=1 SV=2	197,13	0,1
1053	O43169	CYB5B_HUMAN Cytochrome b5 type B OS=Homo sapiens OX=9606 GN=CYB5B PE=1 S	219,94	0,1
1054	P61626	LYSC_HUMAN Lysozyme C OS=Homo sapiens OX=9606 GN=LYZ PE=1 SV=1	5660,62	0,1
1055	Q5JQS6	GSAML_HUMAN Germinal center-associated signaling and motility-like protein OS=Ho	191,65	0,1
1056	P78412	IRX6_HUMAN Iroquois-class homeodomain protein IRX-6 OS=Homo sapiens OX=9606	243,83	0,1
1057	Q92526	TCPW_HUMAN T-complex protein 1 subunit zeta-2 OS=Homo sapiens OX=9606 GN=C	241,31	0,1
1058	P02808	STAT_HUMAN Statherin OS=Homo sapiens OX=9606 GN=STATH PE=1 SV=2	3694,5	0,1
1059	P23142	FBLN1_HUMAN Fibulin-1 OS=Homo sapiens OX=9606 GN=FBLN1 PE=1 SV=4	237,66	0,1
1060	O15091	MRPP3_HUMAN Mitochondrial ribonuclease P catalytic subunit OS=Homo sapiens OX	400,86	0,1
1061	Q8NDX1	PSD4_HUMAN PH and SEC7 domain-containing protein 4 OS=Homo sapiens OX=9606	170,19	0,1
1062	O95445	APOM_HUMAN Apolipoprotein M OS=Homo sapiens OX=9606 GN=APOM PE=1 SV=2	252,66	0,1
1063	Q9BT78	CSN4_HUMAN COP9 signalosome complex subunit 4 OS=Homo sapiens OX=9606 GN=	205,17	0,1
1064	Q8TDB6	DTX3L_HUMAN E3 ubiquitin-protein ligase DTX3L OS=Homo sapiens OX=9606 GN=DT	290,12	0,1
1065	O60318	GANP_HUMAN Germinal-center associated nuclear protein OS=Homo sapiens OX=960	225,2	0,1
1066	O76011	KRT34_HUMAN Keratin_ type I cuticular Ha4 OS=Homo sapiens OX=9606 GN=KRT34 P	4891,75	0,1
1067	O76009	KT33A_HUMAN Keratin_ type I cuticular Ha3-I OS=Homo sapiens OX=9606 GN=KRT33	6496,27	0,1
