# Supplementary material for: Inhibition of the mTOR pathway and reprogramming of protein synthesis by MDM4 reduce ovarian cancer metastatic properties
Source: Cell Death Dis. 2021 May 29;12(6):558. doi: 10.1038/s41419-021-03828-z (PMC8164635; doi:10.1038/s41419-021-03828-z)
Supplement: Supplementary file 2 — Supplementary Table S2 [file 41419_2021_3828_MOESM2_ESM.pdf]

**Table S2. Mdm4-ID8 vs Empty Vector-ID8 MCTSs**

Accession * Unique protein sequence identifier according to UniProtKB/Swiss-Prot Protein Knowledgebase, release 2011_06 of 31-May-11.

PLGS Score§ ProteinLynx Global Server score.

Mdm4/EMPTY Ratio§§ Ratio of expression between the two experimental groups. An arbitrary ratio value of 10 or 0.1 is attributed to a protein found "highly represented" in the numerator (Mdm4) or in the denominator (Empty) of the fraction, respectively.

| | Accession* | Description | PLGS Score§ | Mdm4/EMPTY Ratio§§ |
|---|---|---|---|---|
| 1 | Q64436 | ATP4A_MOUSE Potassium-transporting ATPase alpha chain 1 OS=Mus | 303,29 | 10 |
| 2 | Q80U62 | RUBIC_MOUSE Run domain Beclin-1-interacting and cysteine-rich dor | 223,22 | 10 |
| 3 | Q6A0A2 | LAR4B_MOUSE La-related protein 4B OS=Mus musculus OX=10090 GN | 273,08 | 10 |
| 4 | Q78XF5 | OSTC_MOUSE Oligosaccharyltransferase complex subunit OSTC OS=M | 218,3 | 10 |
| 5 | P17710 | HXK1_MOUSE Hexokinase-1 OS=Mus musculus OX=10090 GN=Hk1 PE | 273,42 | 10 |
| 6 | P67871 | CSK2B_MOUSE Casein kinase II subunit beta OS=Mus musculus OX=1( | 252,1 | 10 |
| 7 | P63166 | SUMO1_MOUSE Small ubiquitin-related modifier 1 OS=Mus musculus | 439,59 | 10 |
| 8 | Q80X95 | RRAGA_MOUSE Ras-related GTP-binding protein A OS=Mus musculus | 228,16 | 10 |
| 9 | Q8K268 | ABCF3_MOUSE ATP-binding cassette sub-family F member 3 OS=Mus | 464,5 | 10 |
| 10 | Q6ZWY6 | U2D2B_MOUSE Ubiquitin-conjugating enzyme E2 D2B OS=Mus muscu | 253,91 | 10 |
| 11 | P22682 | CBL_MOUSE E3 ubiquitin-protein ligase CBL OS=Mus musculus OX=10 | 255,58 | 10 |
| 12 | Q9CZY3 | UB2V1_MOUSE Ubiquitin-conjugating enzyme E2 variant 1 OS=Mus m | 829,1 | 10 |
| 13 | Q9QXF8 | GNMT_MOUSE Glycine N-methyltransferase OS=Mus musculus OX=1( | 198,97 | 10 |
| 14 | Q9D937 | CK098_MOUSE Uncharacterized protein C11orf98 homolog OS=Mus r | 327,75 | 10 |
| 15 | Q8BUV8 | GP107_MOUSE Protein GPR107 OS=Mus musculus OX=10090 GN=Gp | 315,2 | 10 |
| 16 | Q01237 | HMDH_MOUSE 3-hydroxy-3-methylglutaryl-coenzyme A reductase OS | 429,38 | 10 |
| 17 | G5E8Z2 | TAF4B_MOUSE Transcription initiation factor TFIID subunit 4B OS=Mu | 385,82 | 10 |
| 18 | Q3UD01 | A7L3B_MOUSE Ataxin-7-like protein 3B OS=Mus musculus OX=10090 | 350,63 | 10 |
| 19 | Q3UPH7 | ARH40_MOUSE Rho guanine nucleotide exchange factor 40 OS=Mus r | 223,46 | 10 |
| 20 | O08528 | HXK2_MOUSE Hexokinase-2 OS=Mus musculus OX=10090 GN=Hk2 PE | 213,18 | 10 |
| 21 | Q9D8P4 | RM17_MOUSE 39S ribosomal protein L17_ mitochondrial OS=Mus mu | 228,46 | 10 |
| 22 | O55060 | TPMT_MOUSE Thiopurine S-methyltransferase OS=Mus musculus OX= | 216,8 | 10 |
| 23 | Q9Z185 | PADI1_MOUSE Protein-arginine deiminase type-1 OS=Mus musculus ( | 228,53 | 10 |
| 24 | P28271 | ACOC_MOUSE Cytoplasmic aconitate hydratase OS=Mus musculus OX | 224,91 | 10 |
| 25 | P14733 | LMNB1_MOUSE Lamin-B1 OS=Mus musculus OX=10090 GN=Lmnb1 P | 208,23 | 10 |
| 26 | Q80ZW0 | STK35_MOUSE Serine/threonine-protein kinase 35 OS=Mus musculus | 260,42 | 10 |
| 27 | P59900 | EMIL3_MOUSE EMILIN-3 OS=Mus musculus OX=10090 GN=Emilin3 PE | 338,07 | 10 |
| 28 | Q8K1M6 | DNM1L_MOUSE Dynamin-1-like protein OS=Mus musculus OX=10090 | 217,22 | 10 |
| 29 | Q91YK2 | RRP1B_MOUSE Ribosomal RNA processing protein 1 homolog B OS=M | 215,98 | 10 |
| 30 | P70248 | MYO1F_MOUSE Unconventional myosin-If OS=Mus musculus OX=100 | 204,69 | 10 |
| 31 | Q6PDL0 | DC1L2_MOUSE Cytoplasmic dynein 1 light intermediate chain 2 OS=M | 312,69 | 10 |
| 32 | Q8C0D7 | ING4_MOUSE Inhibitor of growth protein 4 OS=Mus musculus OX=10( | 453,55 | 10 |
| 33 | O55023 | IMPA1_MOUSE Inositol monophosphatase 1 OS=Mus musculus OX=1( | 239,84 | 10 |
| 34 | P53368 | 8ODP_MOUSE 7_8-dihydro-8-oxoguanine triphosphatase OS=Mus mu | 331,85 | 10 |
| 35 | Q91YI6 | GLD2_MOUSE Poly(A) RNA polymerase GLD2 OS=Mus musculus OX=1 | 194,01 | 10 |
| 36 | Q9Z140 | CPNE6_MOUSE Copine-6 OS=Mus musculus OX=10090 GN=Cpne6 PE= | 280,43 | 10 |
| 37 | Q2NL51 | GSK3A_MOUSE Glycogen synthase kinase-3 alpha OS=Mus musculus ( | 267,46 | 10 |
| 38 | Q0VE82 | CPNE7_MOUSE Copine-7 OS=Mus musculus OX=10090 GN=Cpne7 PE= | 284,37 | 10 |
| 39 | O08810 | U5S1_MOUSE 116 kDa U5 small nuclear ribonucleoprotein componer | 394,86 | 10 |
| 40 | Q8VEM1 | GOLI_MOUSE E3 ubiquitin-protein ligase RNF130 OS=Mus musculus C | 262,61 | 10 |
| 41 | Q3UZ39 | LRRF1_MOUSE Leucine-rich repeat flightless-interacting protein 1 OS= | 366,02 | 10 |
| 42 | Q8K1I3 | SPP24_MOUSE Secreted phosphoprotein 24 OS=Mus musculus OX=1( | 297,67 | 10 |
| 43 | Q3TXS7 | PSMD1_MOUSE 26S proteasome non-ATPase regulatory subunit 1 OS | 324,4 | 10 |
| 44 | Q9Z0P5 | TWF2_MOUSE Twinfilin-2 OS=Mus musculus OX=10090 GN=Twf2 PE= | 252,31 | 10 |
| 45 | Q4KWH5 | PLCH1_MOUSE 1-phosphatidylinositol 4_5-bisphosphate phosphodie: | 258,4 | 10 |

| | | | | |
|---|---|---|---|---|
| 46 | Q8R1Q8 | DC1L1_MOUSE Cytoplasmic dynein 1 light intermediate chain 1 OS=M | 208,83 | 10 |
| 47 | P08071 | TRFL_MOUSE Lactotransferrin OS=Mus musculus OX=10090 GN=Ltf PI | 192,53 | 10 |
| 48 | P60122 | RUVB1_MOUSE RuvB-like 1 OS=Mus musculus OX=10090 GN=Ruvbl1 | 626,29 | 10 |
| 49 | Q8BXQ2 | PIGT_MOUSE GPI transamidase component PIG-T OS=Mus musculus ( | 275,18 | 10 |
| 50 | P61202 | CSN2_MOUSE COP9 signalosome complex subunit 2 OS=Mus musculu | 292,04 | 10 |
| 51 | Q8CFI5 | SYPM_MOUSE Probable proline--tRNA ligase_ mitochondrial OS=Mus | 198,63 | 10 |
| 52 | Q920P5 | KAD5_MOUSE Adenylate kinase isoenzyme 5 OS=Mus musculus OX=1 | 244,65 | 10 |
| 53 | Q4G0F8 | UBN1_MOUSE Ubinuclein-1 OS=Mus musculus OX=10090 GN=Ubn1 F | 570,43 | 10 |
| 54 | P29621 | SPA3C_MOUSE Serine protease inhibitor A3C OS=Mus musculus OX=1 | 234,75 | 10 |
| 55 | Q60973 | RBBP7_MOUSE Histone-binding protein RBBP7 OS=Mus musculus OX= | 317,01 | 10 |
| 56 | Q60972 | RBBP4_MOUSE Histone-binding protein RBBP4 OS=Mus musculus OX= | 205,59 | 10 |
| 57 | P97742 | CPT1A_MOUSE Carnitine O-palmitoyltransferase 1_ liver isoform OS= | 321,56 | 10 |
| 58 | P10648 | GSTA2_MOUSE Glutathione S-transferase A2 OS=Mus musculus OX=1 | 324,95 | 10 |
| 59 | A3KGS3 | RGPA2_MOUSE Ral GTPase-activating protein subunit alpha-2 OS=Mu | 306,36 | 10 |
| 60 | Q8BKT7 | THOC5_MOUSE THO complex subunit 5 homolog OS=Mus musculus C | 591,66 | 10 |
| 61 | Q9CQ60 | 6PGL_MOUSE 6-phosphogluconolactonase OS=Mus musculus OX=10( | 806,8 | 10 |
| 62 | P62960 | YBOX1_MOUSE Nuclease-sensitive element-binding protein 1 OS=Mu | 596,28 | 10 |
| 63 | P53986 | MOT1_MOUSE Monocarboxylate transporter 1 OS=Mus musculus OX= | 285,08 | 10 |
| 64 | Q8K4G1 | LTBP4_MOUSE Latent-transforming growth factor beta-binding protei | 282,89 | 10 |
| 65 | O35143 | ATIF1_MOUSE ATPase inhibitor_ mitochondrial OS=Mus musculus OX | 694,26 | 10 |
| 66 | P57080 | UBP25_MOUSE Ubiquitin carboxyl-terminal hydrolase 25 OS=Mus mu | 124,48 | 10 |
| 67 | Q8VHI7 | DRC2_MOUSE Dynein regulatory complex subunit 2 OS=Mus musculu | 569,09 | 10 |
| 68 | Q8CII2 | CD123_MOUSE Cell division cycle protein 123 homolog OS=Mus musc | 1088,9 | 10 |
| 69 | Q4KML4 | ABRAL_MOUSE Costars family protein ABRACL OS=Mus musculus OX= | 196,81 | 10 |
| 70 | Q8VHH7 | ADCY3_MOUSE Adenylate cyclase type 3 OS=Mus musculus OX=1009( | 320,95 | 10 |
| 71 | Q9DB15 | RM12_MOUSE 39S ribosomal protein L12_ mitochondrial OS=Mus mu | 272,96 | 10 |
| 72 | Q3UFB2 | BCD1_MOUSE Box C/D snoRNA protein 1 OS=Mus musculus OX=1009 | 212,69 | 10 |
| 73 | Q8BHI4 | KBTB3_MOUSE Kelch repeat and BTB domain-containing protein 3 OS | 228,63 | 10 |
| 74 | O88544 | CSN4_MOUSE COP9 signalosome complex subunit 4 OS=Mus musculu | 230,72 | 10 |
| 75 | Q6GQT9 | NOMO1_MOUSE Nodal modulator 1 OS=Mus musculus OX=10090 GN | 230,85 | 10 |
| 76 | O88531 | PPT1_MOUSE Palmitoyl-protein thioesterase 1 OS=Mus musculus OX= | 196,67 | 10 |
| 77 | Q6IFX3 | K1C40_MOUSE Keratin_ type I cytoskeletal 40 OS=Mus musculus OX= | 592,68 | 10 |
| 78 | Q6PGB8 | SMCA1_MOUSE Probable global transcription activator SNF2L1 OS=M | 357,3 | 10 |
| 79 | Q56A07 | SCN2B_MOUSE Sodium channel subunit beta-2 OS=Mus musculus OX | 198,51 | 10 |
| 80 | P10925 | ZFY1_MOUSE Zinc finger Y-chromosomal protein 1 OS=Mus musculus | 233,22 | 10 |
| 81 | P40240 | CD9_MOUSE CD9 antigen OS=Mus musculus OX=10090 GN=Cd9 PE=1 | 395,95 | 10 |
| 82 | O35459 | ECH1_MOUSE Delta(3_5)-Delta(2_4)-dienoyl-CoA isomerase_ mitocho | 201,44 | 10 |
| 83 | Q8VHE0 | SEC63_MOUSE Translocation protein SEC63 homolog OS=Mus muscul | 206,95 | 10 |
| 84 | Q99JB2 | STML2_MOUSE Stomatin-like protein 2_ mitochondrial OS=Mus musc | 267,15 | 10 |
| 85 | Q3UP24 | NLRC4_MOUSE NLR family CARD domain-containing protein 4 OS=Mu | 247,95 | 10 |
| 86 | Q8BR90 | CE051_MOUSE UPF0600 protein C5orf51 homolog OS=Mus musculus | 207,96 | 10 |
| 87 | P61804 | DAD1_MOUSE Dolichyl-diphosphooligosaccharide--protein glycosyltra | 1624,78 | 10 |
| 88 | Q80T74 | KLH29_MOUSE Kelch-like protein 29 OS=Mus musculus OX=10090 GN | 606,61 | 10 |
| 89 | Q61081 | CDC37_MOUSE Hsp90 co-chaperone Cdc37 OS=Mus musculus OX=10( | 635,5 | 10 |
| 90 | O88866 | HUNK_MOUSE Hormonally up-regulated neu tumor-associated kinase | 303,61 | 10 |
| 91 | O54833 | CSK22_MOUSE Casein kinase II subunit alpha' OS=Mus musculus OX=1 | 207,29 | 10 |
| 92 | Q61074 | PPM1G_MOUSE Protein phosphatase 1G OS=Mus musculus OX=1009 | 202,61 | 10 |
| 93 | Q91XV3 | BASP1_MOUSE Brain acid soluble protein 1 OS=Mus musculus OX=10( | 214,32 | 10 |
| 94 | Q6ZQ12 | NINL_MOUSE Ninein-like protein OS=Mus musculus OX=10090 GN=Ni | 297,16 | 10 |
| 95 | Q5SUV5 | MYLK4_MOUSE Myosin light chain kinase family member 4 OS=Mus n | 289,9 | 10 |
| 96 | P48024 | EIF1_MOUSE Eukaryotic translation initiation factor 1 OS=Mus muscu | 361,23 | 10 |
| 97 | Q69ZN7 | MYOF_MOUSE Myoferlin OS=Mus musculus OX=10090 GN=Myof PE= | 266,22 | 10 |
| 98 | Q64674 | SPEE_MOUSE Spermidine synthase OS=Mus musculus OX=10090 GN= | 251,14 | 10 |
| 99 | Q8BHA3 | DTD2_MOUSE D-aminoacyl-tRNA deacylase 2 OS=Mus musculus OX=1 | 275,71 | 10 |
| 100 | Q9QZN4 | FBX6_MOUSE F-box only protein 6 OS=Mus musculus OX=10090 GN=I | 209,75 | 10 |

| | | | | |
|---|---|---|---|---|
| 101 | Q3V3R1 | C1TM_MOUSE Monofunctional C1-tetrahydrofolate synthase_ mitoch | 204,95 | 10 |
| 102 | Q9ERE7 | MESD_MOUSE LRP chaperone MESD OS=Mus musculus OX=10090 GN | 246,65 | 10 |
| 103 | Q69ZL1 | FGD6_MOUSE FYVE_ RhoGEF and PH domain-containing protein 6 OS | 145,47 | 10 |
| 104 | Q61035 | SYHC_MOUSE Histidine--tRNA ligase_ cytoplasmic OS=Mus musculus | 260,25 | 10 |
| 105 | Q61390 | TCPW_MOUSE T-complex protein 1 subunit zeta-2 OS=Mus musculus | 371,83 | 10 |
| 106 | P06797 | CATL1_MOUSE Cathepsin L1 OS=Mus musculus OX=10090 GN=Ctsl PE | 299,94 | 10 |
| 107 | Q8VDW0 | DX39A_MOUSE ATP-dependent RNA helicase DDX39A OS=Mus muscu | 239,86 | 10 |
| 108 | E9PY46 | IF140_MOUSE Intraflagellar transport protein 140 homolog OS=Mus r | 258,4 | 10 |
| 109 | Q69ZK0 | PREX1_MOUSE Phosphatidylinositol 3_4_5-trisphosphate-dependent | 322,63 | 10 |
| 110 | Q61024 | ASNS_MOUSE Asparagine synthetase [glutamine-hydrolyzing] OS=Mu | 339,25 | 10 |
| 111 | O08792 | COE2_MOUSE Transcription factor COE2 OS=Mus musculus OX=1009( | 378,29 | 10 |
| 112 | Q8BKC5 | IPO5_MOUSE Importin-5 OS=Mus musculus OX=10090 GN=Ipo5 PE=1 | 254,01 | 10 |
| 113 | Q32MW3 | ACO10_MOUSE Acyl-coenzyme A thioesterase 10_ mitochondrial OS= | 364,73 | 10 |
| 114 | Q62448 | IF4G2_MOUSE Eukaryotic translation initiation factor 4 gamma 2 OS= | 199,77 | 10 |
| 115 | Q66JS6 | EI3JB_MOUSE Eukaryotic translation initiation factor 3 subunit J-B OS | 293,82 | 10 |
| 116 | B1AQ75 | KRT36_MOUSE Keratin_ type I cuticular Ha6 OS=Mus musculus OX=1( | 607,53 | 10 |
| 117 | Q62446 | FKBP3_MOUSE Peptidyl-prolyl cis-trans isomerase FKBP3 OS=Mus mu | 723,47 | 10 |
| 118 | Q02566 | MYH6_MOUSE Myosin-6 OS=Mus musculus OX=10090 GN=Myh6 PE= | 201,57 | 10 |
| 119 | P63005 | LIS1_MOUSE Platelet-activating factor acetylhydrolase IB subunit alph | 233,92 | 10 |
| 120 | Q9WVM1 | RGAP1_MOUSE Rac GTPase-activating protein 1 OS=Mus musculus O | 219,82 | 10 |
| 121 | Q8BGZ4 | CDC23_MOUSE Cell division cycle protein 23 homolog OS=Mus muscu | 1136,72 | 10 |
| 122 | Q9D4H1 | EXOC2_MOUSE Exocyst complex component 2 OS=Mus musculus OX= | 208,99 | 10 |
| 123 | P0DN34 | NDUB1_MOUSE NADH dehydrogenase [ubiquinone] 1 beta subcompl | 1279,88 | 10 |
| 124 | P13542 | MYH8_MOUSE Myosin-8 OS=Mus musculus OX=10090 GN=Myh8 PE= | 273,68 | 10 |
| 125 | P13541 | MYH3_MOUSE Myosin-3 OS=Mus musculus OX=10090 GN=Myh3 PE= | 284,42 | 10 |
| 126 | Q8R180 | ERO1A_MOUSE ERO1-like protein alpha OS=Mus musculus OX=10090 | 362,11 | 10 |
| 127 | Q8BGX0 | TRI23_MOUSE E3 ubiquitin-protein ligase TRIM23 OS=Mus musculus ( | 308,8 | 10 |
| 128 | Q8VDP6 | CDIPT_MOUSE CDP-diacylglycerol--inositol 3-phosphatidyltransferase | 233,49 | 10 |
| 129 | P11352 | GPX1_MOUSE Glutathione peroxidase 1 OS=Mus musculus OX=10090 | 296,11 | 10 |
| 130 | Q32Q92 | ACOT6_MOUSE Acyl-coenzyme A thioesterase 6 OS=Mus musculus O | 259,72 | 10 |
| 131 | P32921 | SYWC_MOUSE Tryptophan--tRNA ligase_ cytoplasmic OS=Mus muscu | 274,8 | 10 |
| 132 | Q9QZD9 | EIF3I_MOUSE Eukaryotic translation initiation factor 3 subunit I OS=M | 394,43 | 10 |
| 133 | Q8R151 | ZNFX1_MOUSE NFX1-type zinc finger-containing protein 1 OS=Mus m | 280,94 | 10 |
| 134 | Q8CI51 | PDLI5_MOUSE PDZ and LIM domain protein 5 OS=Mus musculus OX= | 335,56 | 10 |
| 135 | Q80VM7 | ANR24_MOUSE Ankyrin repeat domain-containing protein 24 OS=Mu | 326,24 | 10 |
| 136 | Q8K411 | PREP_MOUSE Presequence protease_ mitochondrial OS=Mus muscul | 205,84 | 10 |
| 137 | Q8BZZ3 | WWP1_MOUSE NEDD4-like E3 ubiquitin-protein ligase WWP1 OS=Mu | 265,87 | 10 |
| 138 | P20662 | ZFY2_MOUSE Zinc finger Y-chromosomal protein 2 OS=Mus musculus | 233,22 | 10 |
| 139 | Q9Z2W1 | STK25_MOUSE Serine/threonine-protein kinase 25 OS=Mus musculus | 248,79 | 10 |
| 140 | Q80Z25 | OFD1_MOUSE Oral-facial-digital syndrome 1 protein homolog OS=Mu | 581,18 | 10 |
| 141 | P54310 | LIPS_MOUSE Hormone-sensitive lipase OS=Mus musculus OX=10090 ( | 303,87 | 10 |
| 142 | P97310 | MCM2_MOUSE DNA replication licensing factor MCM2 OS=Mus musc | 258,11 | 10 |
| 143 | Q8CHP8 | PGP_MOUSE Glycerol-3-phosphate phosphatase OS=Mus musculus O | 239,82 | 10 |
| 144 | Q9ER72 | SYCC_MOUSE Cysteine--tRNA ligase_ cytoplasmic OS=Mus musculus ( | 315,51 | 10 |
| 145 | P47857 | PFKAM_MOUSE ATP-dependent 6-phosphofructokinase_ muscle type | 203,88 | 10 |
| 146 | Q99LS3 | SERB_MOUSE Phosphoserine phosphatase OS=Mus musculus OX=10( | 752,81 | 10 |
| 147 | Q6P9R4 | ARHGI_MOUSE Rho guanine nucleotide exchange factor 18 OS=Mus r | 278,48 | 10 |
| 148 | Q6P6L0 | FIL1L_MOUSE Filamin A-interacting protein 1-like OS=Mus musculus ( | 226,08 | 10 |
| 149 | Q6P9R2 | OXSR1_MOUSE Serine/threonine-protein kinase OSR1 OS=Mus muscu | 755,42 | 10 |
| 150 | Q569L8 | CENPJ_MOUSE Centromere protein J OS=Mus musculus OX=10090 GN | 194,08 | 10 |
| 151 | O35085 | ARX_MOUSE Homeobox protein ARX OS=Mus musculus OX=10090 GN | 341,74 | 10 |
| 152 | Q60875 | ARHG2_MOUSE Rho guanine nucleotide exchange factor 2 OS=Mus m | 204,89 | 10 |
| 153 | Q8BWN8 | ACOT4_MOUSE Acyl-coenzyme A thioesterase 4 OS=Mus musculus O | 194,64 | 10 |
| 154 | A2A690 | TANC2_MOUSE Protein TANC2 OS=Mus musculus OX=10090 GN=Tan( | 287,93 | 10 |
| 155 | Q9CYI4 | LUC7L_MOUSE Putative RNA-binding protein Luc7-like 1 OS=Mus mus | 245,46 | 10 |

| | | | | |
|---|---|---|---|---|
| 156 | Q4VA61 | DSCL1_MOUSE Down syndrome cell adhesion molecule-like protein 1 | 319,81 | 10 |
| 157 | Q9Z2Q5 | RM40_MOUSE 39S ribosomal protein L40_ mitochondrial OS=Mus mu | 217,94 | 10 |
| 158 | Q99P72 | RTN4_MOUSE Reticulon-4 OS=Mus musculus OX=10090 GN=Rtn4 PE= | 268,07 | 10 |
| 159 | Q8R3R8 | GBRL1_MOUSE Gamma-aminobutyric acid receptor-associated protei | 257,59 | 10 |
| 160 | Q4VA53 | PDS5B_MOUSE Sister chromatid cohesion protein PDS5 homolog B OS | 200,23 | 10 |
| 161 | Q8BJS8 | MTBP_MOUSE Mdm2-binding protein OS=Mus musculus OX=10090 G | 251,95 | 10 |
| 162 | P60334 | CDO1_MOUSE Cysteine dioxygenase type 1 OS=Mus musculus OX=10( | 441,5 | 10 |
| 163 | Q9D7A6 | SRP19_MOUSE Signal recognition particle 19 kDa protein OS=Mus mu | 337,65 | 10 |
| 164 | O88487 | DC1I2_MOUSE Cytoplasmic dynein 1 intermediate chain 2 OS=Mus m | 248,97 | 10 |
| 165 | Q69Z99 | ZN512_MOUSE Zinc finger protein 512 OS=Mus musculus OX=10090 ( | 235,1 | 10 |
| 166 | A1L317 | K1C24_MOUSE Keratin_ type I cytoskeletal 24 OS=Mus musculus OX= | 582,91 | 10 |
| 167 | Q922Q4 | P5CR2_MOUSE Pyrroline-5-carboxylate reductase 2 OS=Mus musculu | 363,51 | 10 |
| 168 | Q7TNC4 | LC7L2_MOUSE Putative RNA-binding protein Luc7-like 2 OS=Mus mus | 240,57 | 10 |
| 169 | Q810U5 | CCD50_MOUSE Coiled-coil domain-containing protein 50 OS=Mus mu | 390,27 | 10 |
| 170 | Q9D125 | RT25_MOUSE 28S ribosomal protein S25_ mitochondrial OS=Mus mu: | 303,17 | 10 |
| 171 | P10852 | 4F2_MOUSE 4F2 cell-surface antigen heavy chain OS=Mus musculus ( | 395,22 | 10 |
| 172 | Q2KN98 | CYTSA_MOUSE Cytospin-A OS=Mus musculus OX=10090 GN=Specc1l I | 239,12 | 10 |
| 173 | Q9D3V5 | FSIP1_MOUSE Fibrous sheath-interacting protein 1 OS=Mus musculus | 429,05 | 10 |
| 174 | O54774 | AP3D1_MOUSE AP-3 complex subunit delta-1 OS=Mus musculus OX=: | 163,98 | 10 |
| 175 | Q64261 | CDK6_MOUSE Cyclin-dependent kinase 6 OS=Mus musculus OX=1009 | 213,39 | 10 |
| 176 | Q62083 | PICK1_MOUSE PRKCA-binding protein OS=Mus musculus OX=10090 G | 302,25 | 10 |
| 177 | O54754 | AOXA_MOUSE Aldehyde oxidase 1 OS=Mus musculus OX=10090 GN=/ | 266,94 | 10 |
| 178 | P59108 | CPNE2_MOUSE Copine-2 OS=Mus musculus OX=10090 GN=Cpne2 PE= | 195,23 | 10 |
| 179 | Q9Z2I0 | LETM1_MOUSE Mitochondrial proton/calcium exchanger protein OS= | 208,58 | 10 |
| 180 | Q9D0M3 | CY1_MOUSE Cytochrome c1_ heme protein_ mitochondrial OS=Mus r | 299,39 | 10 |
| 181 | Q9CS42 | PRPS2_MOUSE Ribose-phosphate pyrophosphokinase 2 OS=Mus mus | 280,29 | 10 |
| 182 | Q9EQJ9 | MAGI3_MOUSE Membrane-associated guanylate kinase_ WW and PD | 258,01 | 10 |
| 183 | Q9JM14 | NT5C_MOUSE 5'(3')-deoxyribonucleotidase_ cytosolic type OS=Mus m | 285,47 | 10 |
| 184 | O54749 | CP2J5_MOUSE Cytochrome P450 2J5 OS=Mus musculus OX=10090 GN | 358,17 | 10 |
| 185 | P53810 | PIPNA_MOUSE Phosphatidylinositol transfer protein alpha isoform OS | 308,41 | 10 |
| 186 | Q8BGE6 | ATG4B_MOUSE Cysteine protease ATG4B OS=Mus musculus OX=1009 | 238,27 | 10 |
| 187 | E9Q634 | MYO1E_MOUSE Unconventional myosin-Ie OS=Mus musculus OX=10( | 203,62 | 10 |
| 188 | Q99LF4 | RTCB_MOUSE tRNA-splicing ligase RtcB homolog OS=Mus musculus O | 248,94 | 10 |
| 189 | Q6NTA4 | RRAGB_MOUSE Ras-related GTP-binding protein B OS=Mus musculus | 228,16 | 10 |
| 190 | Q9WUV0 | ORC5_MOUSE Origin recognition complex subunit 5 OS=Mus musculu | 209,63 | 10 |
| 191 | Q3U1V6 | UEVLD_MOUSE Ubiquitin-conjugating enzyme E2 variant 3 OS=Mus m | 230,24 | 10 |
| 192 | P01811 | HVM41_MOUSE Ig heavy chain V region UPC10 OS=Mus musculus OX | 396,53 | 10 |
| 193 | P01810 | HVM40_MOUSE Ig heavy chain V region J539 OS=Mus musculus OX=1 | 396,53 | 10 |
| 194 | Q9WV60 | GSK3B_MOUSE Glycogen synthase kinase-3 beta OS=Mus musculus O | 259,46 | 10 |
| 195 | P01808 | HVM38_MOUSE Ig heavy chain V region T601 OS=Mus musculus OX=: | 396,53 | 10 |
| 196 | P01807 | HVM37_MOUSE Ig heavy chain V region X44 OS=Mus musculus OX=1( | 396,53 | 10 |
| 197 | P01806 | HVM36_MOUSE Ig heavy chain V region 441 OS=Mus musculus OX=1( | 396,53 | 10 |
| 198 | P23591 | FCL_MOUSE GDP-L-fucose synthase OS=Mus musculus OX=10090 GN= | 279,72 | 10 |
| 199 | Q64213 | SF01_MOUSE Splicing factor 1 OS=Mus musculus OX=10090 GN=Sf1 P | 221,52 | 10 |
| 200 | Q6QD59 | SEC20_MOUSE Vesicle transport protein SEC20 OS=Mus musculus OX | 203,39 | 10 |
| 201 | Q62398 | CNGA2_MOUSE Cyclic nucleotide-gated olfactory channel OS=Mus mu | 206,97 | 10 |
| 202 | Q04692 | SMRCD_MOUSE SWI/SNF-related matrix-associated actin-dependent | 224,79 | 10 |
| 203 | O35660 | GSTM6_MOUSE Glutathione S-transferase Mu 6 OS=Mus musculus OX | 264,3 | 10 |
| 204 | Q6NZJ6 | IF4G1_MOUSE Eukaryotic translation initiation factor 4 gamma 1 OS= | 197,42 | 10 |
| 205 | Q91WS0 | CISD1_MOUSE CDGSH iron-sulfur domain-containing protein 1 OS=Mu | 259,81 | 10 |
| 206 | Q9QYM8 | CENPH_MOUSE Centromere protein H OS=Mus musculus OX=10090 ( | 278,59 | 10 |
| 207 | Q91ZX6 | SENP2_MOUSE Sentrin-specific protease 2 OS=Mus musculus OX=100 | 338,48 | 10 |
| 208 | Q05769 | PGH2_MOUSE Prostaglandin G/H synthase 2 OS=Mus musculus OX=1( | 222,67 | 10 |
| 209 | Q62376 | RU17_MOUSE U1 small nuclear ribonucleoprotein 70 kDa OS=Mus mu | 272,49 | 10 |
| 210 | A6PWD2 | FHAD1_MOUSE Forkhead-associated domain-containing protein 1 OS | 341,98 | 10 |

| | | | | |
|---|---|---|---|---|
| 211 | Q9R0Y5 | KAD1_MOUSE Adenylate kinase isoenzyme 1 OS=Mus musculus OX=1 | 307,4 | 10 |
| 212 | Q9D0F9 | PGM1_MOUSE Phosphoglucomutase-1 OS=Mus musculus OX=10090 | 208,79 | 10 |
| 213 | Q91WQ3 | SYYC_MOUSE Tyrosine--tRNA ligase_ cytoplasmic OS=Mus musculus C | 210,94 | 10 |
| 214 | Q9D735 | TRIR_MOUSE Telomerase RNA component interacting RNase OS=Mus | 198,97 | 10 |
| 215 | O35638 | STAG2_MOUSE Cohesin subunit SA-2 OS=Mus musculus OX=10090 GI | 436,43 | 10 |
| 216 | O88700 | BLM_MOUSE Bloom syndrome protein homolog OS=Mus musculus O | 162,02 | 10 |
| 217 | Q922D8 | C1TC_MOUSE C-1-tetrahydrofolate synthase_ cytoplasmic OS=Mus m | 218,4 | 10 |
| 218 | Q9R190 | MTA2_MOUSE Metastasis-associated protein MTA2 OS=Mus musculu | 202 | 10 |
| 219 | Q059Y8 | DCST1_MOUSE E3 ubiquitin-protein ligase DCST1 OS=Mus musculus C | 195,8 | 10 |
| 220 | Q9D9W6 | F217A_MOUSE Protein FAM217A OS=Mus musculus OX=10090 GN=Fa | 258,4 | 10 |
| 221 | P07759 | SPA3K_MOUSE Serine protease inhibitor A3K OS=Mus musculus OX=1 | 231,36 | 10 |
| 222 | A2A8L5 | PTPRF_MOUSE Receptor-type tyrosine-protein phosphatase F OS=Mu | 303,66 | 10 |
| 223 | Q8K019 | BCLF1_MOUSE Bcl-2-associated transcription factor 1 OS=Mus muscu | 244,21 | 10 |
| 224 | Q9CXU9 | EIF1B_MOUSE Eukaryotic translation initiation factor 1b OS=Mus mus | 380,19 | 10 |
| 225 | Q8K009 | AL1L2_MOUSE Mitochondrial 10-formyltetrahydrofolate dehydrogena | 216,67 | 10 |
| 226 | Q9D6N5 | NC2A_MOUSE Dr1-associated corepressor OS=Mus musculus OX=100 | 236 | 10 |
| 227 | Q8VCR8 | MYLK2_MOUSE Myosin light chain kinase 2_ skeletal/cardiac muscle ( | 289,9 | 10 |
| 228 | Q5SX40 | MYH1_MOUSE Myosin-1 OS=Mus musculus OX=10090 GN=Myh1 PE= | 277,97 | 10 |
| 229 | Q5SX39 | MYH4_MOUSE Myosin-4 OS=Mus musculus OX=10090 GN=Myh4 PE= | 255,43 | 10 |
| 230 | E9Q5G3 | KIF23_MOUSE Kinesin-like protein KIF23 OS=Mus musculus OX=1009( | 301,26 | 10 |
| 231 | Q91WK0 | LRRF2_MOUSE Leucine-rich repeat flightless-interacting protein 2 OS= | 359,78 | 10 |
| 232 | Q61581 | IBP7_MOUSE Insulin-like growth factor-binding protein 7 OS=Mus mu | 344,53 | 10 |
| 233 | A7XUZ6 | SKIT6_MOUSE Selection and upkeep of intraepithelial T-cells protein 6 | 552,08 | 10 |
| 234 | Q587J6 | LITD1_MOUSE LINE-1 type transposase domain-containing protein 1 C | 391,04 | 10 |
| 235 | Q8BW75 | AOFB_MOUSE Amine oxidase [flavin-containing] B OS=Mus musculus | 251,47 | 10 |
| 236 | Q8VD04 | GRAP1_MOUSE GRIP1-associated protein 1 OS=Mus musculus OX=10( | 195,56 | 10 |
| 237 | Q9DCD6 | GBRAP_MOUSE Gamma-aminobutyric acid receptor-associated prote | 257,59 | 10 |
| 238 | P49615 | CDK5_MOUSE Cyclin-dependent-like kinase 5 OS=Mus musculus OX=1 | 241,85 | 10 |
| 239 | Q8BT07 | CEP55_MOUSE Centrosomal protein of 55 kDa OS=Mus musculus OX= | 750,5 | 10 |
| 240 | A7XUY5 | SKIT5_MOUSE Selection and upkeep of intraepithelial T-cells protein 5 | 1032 | 10 |
| 241 | Q3UZZ4 | OLFM4_MOUSE Olfactomedin-4 OS=Mus musculus OX=10090 GN=Olf | 258,97 | 10 |
| 242 | Q8K2Q2 | GSTO2_MOUSE Glutathione S-transferase omega-2 OS=Mus musculus | 205,06 | 10 |
| 243 | P47791 | GSHR_MOUSE Glutathione reductase_ mitochondrial OS=Mus muscul | 376,3 | 10 |
| 244 | A7XUX6 | SKIT2_MOUSE Selection and upkeep of intraepithelial T-cells protein 2 | 526,13 | 10 |
| 245 | Q8R2Y8 | PTH2_MOUSE Peptidyl-tRNA hydrolase 2_ mitochondrial OS=Mus mu | 209,84 | 10 |
| 246 | Q8BG51 | MIRO1_MOUSE Mitochondrial Rho GTPase 1 OS=Mus musculus OX=1 | 319,54 | 10 |
| 247 | Q9CRB9 | MIC19_MOUSE MICOS complex subunit Mic19 OS=Mus musculus OX= | 366,08 | 10 |
| 248 | Q497I4 | KRT35_MOUSE Keratin_ type I cuticular Ha5 OS=Mus musculus OX=1( | 580,54 | 10 |
| 249 | Q8R035 | ICT1_MOUSE Peptidyl-tRNA hydrolase ICT1_ mitochondrial OS=Mus m | 345,61 | 10 |
| 250 | Q3UGR5 | HDHD2_MOUSE Haloacid dehalogenase-like hydrolase domain-contai | 319,19 | 10 |
| 251 | Q9QYA2 | TOM40_MOUSE Mitochondrial import receptor subunit TOM40 homo | 212,65 | 10 |
| 252 | Q3U7R1 | ESYT1_MOUSE Extended synaptotagmin-1 OS=Mus musculus OX=100 | 201,06 | 10 |
| 253 | A4Q9E8 | TTLL6_MOUSE Tubulin polyglutamylase TTLL6 OS=Mus musculus OX= | 193,6 | 10 |
| 254 | Q8BFR4 | GNS_MOUSE N-acetylglucosamine-6-sulfatase OS=Mus musculus OX= | 212,89 | 10 |
| 255 | Q3TIX9 | SNUT2_MOUSE U4/U6.U5 tri-snRNP-associated protein 2 OS=Mus mu | 246 | 10 |
| 256 | O55137 | ACOT1_MOUSE Acyl-coenzyme A thioesterase 1 OS=Mus musculus O) | 685,03 | 10 |
| 257 | Q9JI75 | NQO2_MOUSE Ribosyldihydronicotinamide dehydrogenase [quinone] | 642,04 | 10 |
| 258 | P21619 | LMNB2_MOUSE Lamin-B2 OS=Mus musculus OX=10090 GN=Lmnb2 P | 240,61 | 10 |
| 259 | Q91WD5 | NDUS2_MOUSE NADH dehydrogenase [ubiquinone] iron-sulfur protei | 194,48 | 10 |
| 260 | Q5SGK3 | AOXB_MOUSE Aldehyde oxidase 2 OS=Mus musculus OX=10090 GN=/ | 312,91 | 10 |
| 261 | P47740 | AL3A2_MOUSE Fatty aldehyde dehydrogenase OS=Mus musculus OX= | 227,55 | 10 |
| 262 | Q9CXJ1 | SYEM_MOUSE Probable glutamate--tRNA ligase_ mitochondrial OS=M | 262,51 | 10 |
| 263 | Q8VCH0 | THIKB_MOUSE 3-ketoacyl-CoA thiolase B_ peroxisomal OS=Mus musc | 277,47 | 10 |
| 264 | P47739 | AL3A1_MOUSE Aldehyde dehydrogenase_ dimeric NADP-preferring C | 284,67 | 10 |
| 265 | Q1RLL3 | CPNE9_MOUSE Copine-9 OS=Mus musculus OX=10090 GN=Cpne9 PE= | 300,9 | 10 |

| | | | | |
|---|---|---|---|---|
| 266 | Q60767 | LY75_MOUSE Lymphocyte antigen 75 OS=Mus musculus OX=10090 G | 236,84 | 10 |
| 267 | Q9WUA3 | PFKAP_MOUSE ATP-dependent 6-phosphofructokinase_ platelet type | 396,84 | 10 |
| 268 | Q9WUA2 | SYFB_MOUSE Phenylalanine--tRNA ligase beta subunit OS=Mus muscu | 200,84 | 10 |
| 269 | O70157 | TOP3A_MOUSE DNA topoisomerase 3-alpha OS=Mus musculus OX=1( | 247,68 | 10 |
| 270 | P28660 | NCKP1_MOUSE Nck-associated protein 1 OS=Mus musculus OX=1009( | 243,23 | 10 |
| 271 | Q924X2 | CPT1B_MOUSE Carnitine O-palmitoyltransferase 1_ muscle isoform O | 148 | 10 |
| 272 | P54869 | HMCS2_MOUSE Hydroxymethylglutaryl-CoA synthase_ mitochondrial | 290,2 | 10 |
| 273 | Q9D2Y5 | SNX20_MOUSE Sorting nexin-20 OS=Mus musculus OX=10090 GN=Sn | 278,83 | 10 |
| 274 | Q673U1 | HS3S2_MOUSE Heparan sulfate glucosamine 3-O-sulfotransferase 2 C | 217,1 | 10 |
| 275 | O35295 | PURB_MOUSE Transcriptional activator protein Pur-beta OS=Mus mu | 219,96 | 10 |
| 276 | Q9CQW5 | LEG2_MOUSE Galectin-2 OS=Mus musculus OX=10090 GN=Lgals2 PE= | 209,61 | 10 |
| 277 | Q8JZW4 | CPNE5_MOUSE Copine-5 OS=Mus musculus OX=10090 GN=Cpne5 PE= | 308,97 | 10 |
| 278 | P03930 | ATP8_MOUSE ATP synthase protein 8 OS=Mus musculus OX=10090 G | 1293,38 | 10 |
| 279 | Q921N6 | DDX27_MOUSE Probable ATP-dependent RNA helicase DDX27 OS=Mu | 260,82 | 10 |
| 280 | P24270 | CATA_MOUSE Catalase OS=Mus musculus OX=10090 GN=Cat PE=1 SV | 594,68 | 10 |
| 281 | P54822 | PUR8_MOUSE Adenylosuccinate lyase OS=Mus musculus OX=10090 G | 262,07 | 10 |
| 282 | Q9EPL8 | IPO7_MOUSE Importin-7 OS=Mus musculus OX=10090 GN=Ipo7 PE=1 | 206,07 | 10 |
| 283 | P42225 | STAT1_MOUSE Signal transducer and activator of transcription 1 OS=I | 207,18 | 10 |
| 284 | Q9DC53 | CPNE8_MOUSE Copine-8 OS=Mus musculus OX=10090 GN=Cpne8 PE= | 300,9 | 10 |
| 285 | Q9QXT0 | CNPY2_MOUSE Protein canopy homolog 2 OS=Mus musculus OX=100 | 644,84 | 10 |
| 286 | O88327 | CTNL1_MOUSE Alpha-catulin OS=Mus musculus OX=10090 GN=Ctnna | 171,84 | 10 |
| 287 | Q9JL26 | FMNL1_MOUSE Formin-like protein 1 OS=Mus musculus OX=10090 G | 269,57 | 10 |
| 288 | Q921L3 | TMCO1_MOUSE Calcium load-activated calcium channel OS=Mus mu | 343,05 | 10 |
| 289 | Q8BVE8 | NSD2_MOUSE Histone-lysine N-methyltransferase NSD2 OS=Mus mus | 214,19 | 10 |
| 290 | Q8BLR2 | CPNE4_MOUSE Copine-4 OS=Mus musculus OX=10090 GN=Cpne4 PE= | 280,43 | 10 |
| 291 | P60521 | GBRL2_MOUSE Gamma-aminobutyric acid receptor-associated protei | 359,5 | 10 |
| 292 | P10711 | TCEA1_MOUSE Transcription elongation factor A protein 1 OS=Mus m | 229,75 | 10 |
| 293 | Q8BVE3 | VATH_MOUSE V-type proton ATPase subunit H OS=Mus musculus OX= | 216,37 | 10 |
| 294 | Q3U186 | SYRM_MOUSE Probable arginine--tRNA ligase_ mitochondrial OS=Mu | 205,89 | 10 |
| 295 | Q9JHI5 | IVD_MOUSE Isovaleryl-CoA dehydrogenase_ mitochondrial OS=Mus n | 317,7 | 10 |
| 296 | Q9DC23 | DJC10_MOUSE DnaJ homolog subfamily C member 10 OS=Mus muscu | 244,53 | 10 |
| 297 | Q3UGC7 | EI3JA_MOUSE Eukaryotic translation initiation factor 3 subunit J-A OS | 293,82 | 10 |
| 298 | Q921J4 | UBE2S_MOUSE Ubiquitin-conjugating enzyme E2 S OS=Mus musculus | 531,17 | 10 |
| 299 | Q8BIJ6 | SYIM_MOUSE Isoleucine--tRNA ligase_ mitochondrial OS=Mus muscul | 220,3 | 10 |
| 300 | Q03173 | ENAH_MOUSE Protein enabled homolog OS=Mus musculus OX=1009( | 256,75 | 10 |
| 301 | Q6NVG5 | MREG_MOUSE Melanoregulin OS=Mus musculus OX=10090 GN=Mre | 421,53 | 10 |
| 302 | Q9Z1G3 | VATC1_MOUSE V-type proton ATPase subunit C 1 OS=Mus musculus ( | 208,02 | 10 |
| 303 | Q921J2 | RHEB_MOUSE GTP-binding protein Rheb OS=Mus musculus OX=1009( | 312,15 | 10 |
| 304 | Q7TSG3 | FBX5_MOUSE F-box only protein 5 OS=Mus musculus OX=10090 GN=I | 1060,78 | 10 |
| 305 | Q921H8 | THIKA_MOUSE 3-ketoacyl-CoA thiolase A_ peroxisomal OS=Mus musc | 273,25 | 10 |
| 306 | Q8QZY1 | EIF3L_MOUSE Eukaryotic translation initiation factor 3 subunit L OS=N | 320,39 | 10 |
| 307 | Q9QY06 | MYO9B_MOUSE Unconventional myosin-IXb OS=Mus musculus OX=1( | 179,38 | 10 |
| 308 | Q9D662 | SC23B_MOUSE Protein transport protein Sec23B OS=Mus musculus O | 214,3 | 10 |
| 309 | P51863 | VA0D1_MOUSE V-type proton ATPase subunit d 1 OS=Mus musculus ( | 288,67 | 10 |
| 310 | Q8VBX4 | CLC4K_MOUSE C-type lectin domain family 4 member K OS=Mus mus | 200,91 | 10 |
| 311 | Q9WTR1 | TRPV2_MOUSE Transient receptor potential cation channel subfamily | 309,72 | 10 |
| 312 | Q9D2M8 | UB2V2_MOUSE Ubiquitin-conjugating enzyme E2 variant 2 OS=Mus m | 746,67 | 10 |
| 313 | O54946 | DNJB6_MOUSE DnaJ homolog subfamily B member 6 OS=Mus muscul | 392,17 | 10 |
| 314 | Q8BLK9 | KS6C1_MOUSE Ribosomal protein S6 kinase delta-1 OS=Mus musculu | 318,87 | 10 |
| 315 | Q9EQP2 | EHD4_MOUSE EH domain-containing protein 4 OS=Mus musculus OX= | 227,6 | 2,51 |
| 316 | O70475 | UGDH_MOUSE UDP-glucose 6-dehydrogenase OS=Mus musculus OX= | 290,2 | 2,25 |
| 317 | P70336 | ROCK2_MOUSE Rho-associated protein kinase 2 OS=Mus musculus O) | 317,81 | 2,23 |
| 318 | P13745 | GSTA1_MOUSE Glutathione S-transferase A1 OS=Mus musculus OX=1 | 506,13 | 2,18 |
| 319 | P62984 | RL40_MOUSE Ubiquitin-60S ribosomal protein L40 OS=Mus musculus | 4877,87 | 2,16 |
| 320 | P0CG49 | UBB_MOUSE Polyubiquitin-B OS=Mus musculus OX=10090 GN=Ubb P | 4877,87 | 2,16 |

| | | | | |
|---|---|---|---|---|
| 321 | Q9DBS1 | TMM43_MOUSE Transmembrane protein 43 OS=Mus musculus OX=1 | 374,81 | 2,16 |
| 322 | P0CG50 | UBC_MOUSE Polyubiquitin-C OS=Mus musculus OX=10090 GN=Ubc P | 4877,87 | 2,14 |
| 323 | P62983 | RS27A_MOUSE Ubiquitin-40S ribosomal protein S27a OS=Mus muscu | 5470,85 | 2,12 |
| 324 | Q64669 | NQO1_MOUSE NAD(P)H dehydrogenase [quinone] 1 OS=Mus muscul | 3247,86 | 2,12 |
| 325 | P84244 | H33_MOUSE Histone H3.3 OS=Mus musculus OX=10090 GN=H3f3a PE | 806,83 | 2,10 |
| 326 | P84228 | H32_MOUSE Histone H3.2 OS=Mus musculus OX=10090 GN=Hist1h3b | 806,83 | 2,08 |
| 327 | Q8CGK3 | LONM_MOUSE Lon protease homolog_ mitochondrial OS=Mus muscu | 246,79 | 2,08 |
| 328 | P02301 | H3C_MOUSE Histone H3.3C OS=Mus musculus OX=10090 GN=H3f3c F | 806,83 | 2,05 |
| 329 | P68433 | H31_MOUSE Histone H3.1 OS=Mus musculus OX=10090 GN=Hist1h3a | 806,83 | 2,03 |
| 330 | P09405 | NUCL_MOUSE Nucleolin OS=Mus musculus OX=10090 GN=Ncl PE=1 S | 2741,33 | 1,95 |
| 331 | Q9JMH6 | TRXR1_MOUSE Thioredoxin reductase 1_ cytoplasmic OS=Mus muscu | 433,42 | 1,95 |
| 332 | Q8VC28 | AK1CD_MOUSE Aldo-keto reductase family 1 member C13 OS=Mus m | 597,44 | 1,92 |
| 333 | P34152 | FAK1_MOUSE Focal adhesion kinase 1 OS=Mus musculus OX=10090 G | 264,81 | 1,92 |
| 334 | P09528 | FRIH_MOUSE Ferritin heavy chain OS=Mus musculus OX=10090 GN=F | 485,74 | 1,88 |
| 335 | P70298 | CUX2_MOUSE Homeobox protein cut-like 2 OS=Mus musculus OX=10 | 237,77 | 1,86 |
| 336 | P99028 | QCR6_MOUSE Cytochrome b-c1 complex subunit 6_ mitochondrial OS | 350,17 | 1,84 |
| 337 | P38647 | GRP75_MOUSE Stress-70 protein_ mitochondrial OS=Mus musculus C | 4001,73 | 1,84 |
| 338 | P30115 | GSTA3_MOUSE Glutathione S-transferase A3 OS=Mus musculus OX=1 | 253,97 | 1,84 |
| 339 | Q9QYR9 | ACOT2_MOUSE Acyl-coenzyme A thioesterase 2_ mitochondrial OS=M | 216,65 | 1,79 |
| 340 | Q9CZN7 | GLYM_MOUSE Serine hydroxymethyltransferase_ mitochondrial OS=M | 2312,48 | 1,77 |
| 341 | Q99LP6 | GRPE1_MOUSE GrpE protein homolog 1_ mitochondrial OS=Mus mus | 672,79 | 1,77 |
| 342 | Q3UQU0 | BRD9_MOUSE Bromodomain-containing protein 9 OS=Mus musculus | 332,65 | 1,77 |
| 343 | P21300 | ALD1_MOUSE Aldose reductase-related protein 1 OS=Mus musculus ( | 520,05 | 1,75 |
| 344 | P61255 | RL26_MOUSE 60S ribosomal protein L26 OS=Mus musculus OX=1009( | 866,31 | 1,72 |
| 345 | Q9ES00 | UBE4B_MOUSE Ubiquitin conjugation factor E4 B OS=Mus musculus C | 1064,89 | 1,72 |
| 346 | P45376 | ALDR_MOUSE Aldose reductase OS=Mus musculus OX=10090 GN=Akr | 5596,45 | 1,68 |
| 347 | O09131 | GSTO1_MOUSE Glutathione S-transferase omega-1 OS=Mus musculus | 994,64 | 1,68 |
| 348 | P24472 | GSTA4_MOUSE Glutathione S-transferase A4 OS=Mus musculus OX=1 | 2258,91 | 1,67 |
| 349 | P70349 | HINT1_MOUSE Histidine triad nucleotide-binding protein 1 OS=Mus m | 2443,69 | 1,67 |
| 350 | Q791V5 | MTCH2_MOUSE Mitochondrial carrier homolog 2 OS=Mus musculus ( | 355,69 | 1,67 |
| 351 | Q61699 | HS105_MOUSE Heat shock protein 105 kDa OS=Mus musculus OX=10 | 991,71 | 1,65 |
| 352 | P62806 | H4_MOUSE Histone H4 OS=Mus musculus OX=10090 GN=Hist1h4a PE | 10418,57 | 1,65 |
| 353 | Q9R0P3 | ESTD_MOUSE S-formylglutathione hydrolase OS=Mus musculus OX=1 | 5410,91 | 1,65 |
| 354 | P17427 | AP2A2_MOUSE AP-2 complex subunit alpha-2 OS=Mus musculus OX= | 234,94 | 1,65 |
| 355 | Q64522 | H2A2B_MOUSE Histone H2A type 2-B OS=Mus musculus OX=10090 G | 1980,89 | 1,62 |
| 356 | Q9CY50 | SSRA_MOUSE Translocon-associated protein subunit alpha OS=Mus m | 653,8 | 1,62 |
| 357 | Q9WTP6 | KAD2_MOUSE Adenylate kinase 2_ mitochondrial OS=Mus musculus ( | 375,03 | 1,62 |
| 358 | Q61937 | NPM_MOUSE Nucleophosmin OS=Mus musculus OX=10090 GN=Npm | 6856,67 | 1,60 |
| 359 | P10853 | H2B1F_MOUSE Histone H2B type 1-F/J/L OS=Mus musculus OX=1009( | 2156,78 | 1,60 |
| 360 | Q8CGP1 | H2B1K_MOUSE Histone H2B type 1-K OS=Mus musculus OX=10090 GI | 2156,78 | 1,60 |
| 361 | Q64478 | H2B1H_MOUSE Histone H2B type 1-H OS=Mus musculus OX=10090 G | 2156,78 | 1,60 |
| 362 | Q60930 | VDAC2_MOUSE Voltage-dependent anion-selective channel protein 2 | 2688,89 | 1,58 |
| 363 | Q64525 | H2B2B_MOUSE Histone H2B type 2-B OS=Mus musculus OX=10090 GI | 2156,78 | 1,58 |
| 364 | Q8CGP2 | H2B1P_MOUSE Histone H2B type 1-P OS=Mus musculus OX=10090 GI | 2156,78 | 1,58 |
| 365 | Q64475 | H2B1B_MOUSE Histone H2B type 1-B OS=Mus musculus OX=10090 GI | 2156,78 | 1,58 |
| 366 | Q6ZWY9 | H2B1C_MOUSE Histone H2B type 1-C/E/G OS=Mus musculus OX=1009 | 2156,78 | 1,57 |
| 367 | O08756 | HCD2_MOUSE 3-hydroxyacyl-CoA dehydrogenase type-2 OS=Mus mu | 820,3 | 1,57 |
| 368 | P10854 | H2B1M_MOUSE Histone H2B type 1-M OS=Mus musculus OX=10090 ( | 2156,78 | 1,57 |
| 369 | P46978 | STT3A_MOUSE Dolichyl-diphosphooligosaccharide--protein glycosyltr | 447,96 | 1,57 |
| 370 | Q3THW5 | H2AV_MOUSE Histone H2A.V OS=Mus musculus OX=10090 GN=H2afv | 1906,76 | 1,55 |
| 371 | C0HKE9 | H2A1P_MOUSE Histone H2A type 1-P OS=Mus musculus OX=10090 GI | 15777,27 | 1,55 |
| 372 | C0HKE8 | H2A1O_MOUSE Histone H2A type 1-O OS=Mus musculus OX=10090 G | 15777,27 | 1,55 |
| 373 | C0HKE5 | H2A1G_MOUSE Histone H2A type 1-G OS=Mus musculus OX=10090 G | 15777,27 | 1,55 |
| 374 | C0HKE2 | H2A1C_MOUSE Histone H2A type 1-C OS=Mus musculus OX=10090 GI | 15777,27 | 1,55 |
| 375 | Q64523 | H2A2C_MOUSE Histone H2A type 2-C OS=Mus musculus OX=10090 GI | 15777,27 | 1,55 |

| | | | | |
|---|---|---|---|---|
| 376 | Q8CGP7 | H2A1K_MOUSE Histone H2A type 1-K OS=Mus musculus OX=10090 GI | 15777,27 | 1,55 |
| 377 | Q8CGP6 | H2A1H_MOUSE Histone H2A type 1-H OS=Mus musculus OX=10090 G | 15777,27 | 1,55 |
| 378 | P97807 | FUMH_MOUSE Fumarate hydratase_ mitochondrial OS=Mus musculu | 582,28 | 1,55 |
| 379 | P26516 | PSMD7_MOUSE 26S proteasome non-ATPase regulatory subunit 7 OS | 341,68 | 1,55 |
| 380 | P0C0S6 | H2AZ_MOUSE Histone H2A.Z OS=Mus musculus OX=10090 GN=H2afz | 1906,76 | 1,54 |
| 381 | P24452 | CAPG_MOUSE Macrophage-capping protein OS=Mus musculus OX=1( | 1310,54 | 1,54 |
| 382 | P31230 | AIMP1_MOUSE Aminoacyl tRNA synthase complex-interacting multifu | 467,23 | 1,54 |
| 383 | C0HKE7 | H2A1N_MOUSE Histone H2A type 1-N OS=Mus musculus OX=10090 G | 15777,27 | 1,54 |
| 384 | C0HKE6 | H2A1I_MOUSE Histone H2A type 1-I OS=Mus musculus OX=10090 GN | 15777,27 | 1,54 |
| 385 | C0HKE4 | H2A1E_MOUSE Histone H2A type 1-E OS=Mus musculus OX=10090 GI | 15777,27 | 1,54 |
| 386 | C0HKE3 | H2A1D_MOUSE Histone H2A type 1-D OS=Mus musculus OX=10090 G | 15777,27 | 1,54 |
| 387 | C0HKE1 | H2A1B_MOUSE Histone H2A type 1-B OS=Mus musculus OX=10090 G | 15777,27 | 1,54 |
| 388 | P35564 | CALX_MOUSE Calnexin OS=Mus musculus OX=10090 GN=Canx PE=1 S | 2064,47 | 1,54 |
| 389 | P27661 | H2AX_MOUSE Histone H2AX OS=Mus musculus OX=10090 GN=H2afx | 2074,2 | 1,54 |
| 390 | Q6GSS7 | H2A2A_MOUSE Histone H2A type 2-A OS=Mus musculus OX=10090 G | 15777,27 | 1,54 |
| 391 | Q8BFU2 | H2A3_MOUSE Histone H2A type 3 OS=Mus musculus OX=10090 GN=I | 15777,27 | 1,54 |
| 392 | Q8CGP5 | H2A1F_MOUSE Histone H2A type 1-F OS=Mus musculus OX=10090 GI | 15777,27 | 1,54 |
| 393 | P70694 | DHB5_MOUSE Estradiol 17 beta-dehydrogenase 5 OS=Mus musculus ( | 305,14 | 1,54 |
| 394 | Q8R1M2 | H2AJ_MOUSE Histone H2A.J OS=Mus musculus OX=10090 GN=H2afj F | 15777,27 | 1,52 |
| 395 | P14094 | AT1B1_MOUSE Sodium/potassium-transporting ATPase subunit beta- | 504,35 | 1,52 |
| 396 | Q9CXW4 | RL11_MOUSE 60S ribosomal protein L11 OS=Mus musculus OX=1009( | 4110,19 | 1,52 |
| 397 | P70696 | H2B1A_MOUSE Histone H2B type 1-A OS=Mus musculus OX=10090 G | 436,9 | 1,52 |
| 398 | Q9D379 | HYEP_MOUSE Epoxide hydrolase 1 OS=Mus musculus OX=10090 GN= | 2082,41 | 1,52 |
| 399 | Q61753 | SERA_MOUSE D-3-phosphoglycerate dehydrogenase OS=Mus muscul | 1246,57 | 1,51 |
| 400 | Q8R5J9 | PRAF3_MOUSE PRA1 family protein 3 OS=Mus musculus OX=10090 G | 1051,96 | 1,51 |
| 401 | Q80UU9 | PGRC2_MOUSE Membrane-associated progesterone receptor compo | 446,07 | 1,49 |
| 402 | Q9WVK4 | EHD1_MOUSE EH domain-containing protein 1 OS=Mus musculus OX= | 286,73 | 1,49 |
| 403 | Q64524 | H2B2E_MOUSE Histone H2B type 2-E OS=Mus musculus OX=10090 GI | 1545,92 | 1,48 |
| 404 | Q9D2U9 | H2B3A_MOUSE Histone H2B type 3-A OS=Mus musculus OX=10090 G | 1545,92 | 1,48 |
| 405 | P00015 | CYC2_MOUSE Cytochrome c_ testis-specific OS=Mus musculus OX=10 | 967,72 | 1,48 |
| 406 | Q9DCD0 | 6PGD_MOUSE 6-phosphogluconate dehydrogenase_ decarboxylating | 2808,01 | 1,46 |
| 407 | Q8CGP0 | H2B3B_MOUSE Histone H2B type 3-B OS=Mus musculus OX=10090 GI | 1545,92 | 1,46 |
| 408 | Q7TMY4 | THOC7_MOUSE THO complex subunit 7 homolog OS=Mus musculus C | 327,08 | 1,46 |
| 409 | Q9D3D9 | ATPD_MOUSE ATP synthase subunit delta_ mitochondrial OS=Mus mu | 1199,46 | 1,45 |
| 410 | Q8BH04 | PCKGM_MOUSE Phosphoenolpyruvate carboxykinase [GTP]_ mitocho | 277,53 | 1,45 |
| 411 | Q6PDM2 | SRSF1_MOUSE Serine/arginine-rich splicing factor 1 OS=Mus musculu | 1134,34 | 1,43 |
| 412 | P63038 | CH60_MOUSE 60 kDa heat shock protein_ mitochondrial OS=Mus mu | 8334,73 | 1,43 |
| 413 | P45377 | ALD2_MOUSE Aldose reductase-related protein 2 OS=Mus musculus ( | 814,61 | 1,43 |
| 414 | Q9CZ13 | QCR1_MOUSE Cytochrome b-c1 complex subunit 1_ mitochondrial OS | 1109,71 | 1,43 |
| 415 | Q8R429 | AT2A1_MOUSE Sarcoplasmic/endoplasmic reticulum calcium ATPase | 216,01 | 1,42 |
| 416 | O70251 | EF1B_MOUSE Elongation factor 1-beta OS=Mus musculus OX=10090 ( | 2085,14 | 1,40 |
| 417 | O09167 | RL21_MOUSE 60S ribosomal protein L21 OS=Mus musculus OX=1009( | 631,6 | 1,40 |
| 418 | Q8CAQ8 | MIC60_MOUSE MICOS complex subunit Mic60 OS=Mus musculus OX= | 1231,63 | 1,40 |
| 419 | O55022 | PGRC1_MOUSE Membrane-associated progesterone receptor compo | 414,81 | 1,40 |
| 420 | P41731 | CD63_MOUSE CD63 antigen OS=Mus musculus OX=10090 GN=Cd63 P | 918,44 | 1,39 |
| 421 | P48722 | HS74L_MOUSE Heat shock 70 kDa protein 4L OS=Mus musculus OX=1 | 794,26 | 1,39 |
| 422 | Q60931 | VDAC3_MOUSE Voltage-dependent anion-selective channel protein 3 | 2769,58 | 1,39 |
| 423 | P26443 | DHE3_MOUSE Glutamate dehydrogenase 1_ mitochondrial OS=Mus r | 1757,25 | 1,39 |
| 424 | Q3TEA8 | HP1B3_MOUSE Heterochromatin protein 1-binding protein 3 OS=Mus | 359,02 | 1,39 |
| 425 | P53657 | KPYR_MOUSE Pyruvate kinase PKLR OS=Mus musculus OX=10090 GN= | 1501,01 | 1,38 |
| 426 | Q60932 | VDAC1_MOUSE Voltage-dependent anion-selective channel protein 1 | 5176,23 | 1,38 |
| 427 | O35129 | PHB2_MOUSE Prohibitin-2 OS=Mus musculus OX=10090 GN=Phb2 PE | 3230,7 | 1,38 |
| 428 | Q6PIC6 | AT1A3_MOUSE Sodium/potassium-transporting ATPase subunit alpha | 519,66 | 1,38 |
| 429 | P67984 | RL22_MOUSE 60S ribosomal protein L22 OS=Mus musculus OX=1009( | 3933,92 | 1,38 |
| 430 | P50431 | GLYC_MOUSE Serine hydroxymethyltransferase_ cytosolic OS=Mus m | 902,63 | 1,38 |

| | | | | |
|---|---|---|---|---|
| 431 | Q8VEM8 | MPCP_MOUSE Phosphate carrier protein_ mitochondrial OS=Mus mu | 2328,77 | 1,36 |
| 432 | Q60854 | SPB6_MOUSE Serpin B6 OS=Mus musculus OX=10090 GN=Serpinb6 P | 3648,48 | 1,36 |
| 433 | Q8CGC7 | SYEP_MOUSE Bifunctional glutamate/proline--tRNA ligase OS=Mus m | 675,95 | 1,36 |
| 434 | P51410 | RL9_MOUSE 60S ribosomal protein L9 OS=Mus musculus OX=10090 G | 4157,67 | 1,35 |
| 435 | O08795 | GLU2B_MOUSE Glucosidase 2 subunit beta OS=Mus musculus OX=10( | 525,01 | 1,35 |
| 436 | P62897 | CYC_MOUSE Cytochrome c_ somatic OS=Mus musculus OX=10090 GN | 2952 | 1,35 |
| 437 | Q62093 | SRSF2_MOUSE Serine/arginine-rich splicing factor 2 OS=Mus musculu | 4874,54 | 1,35 |
| 438 | P45591 | COF2_MOUSE Cofilin-2 OS=Mus musculus OX=10090 GN=Cfl2 PE=1 S\ | 2279,52 | 1,35 |
| 439 | P35700 | PRDX1_MOUSE Peroxiredoxin-1 OS=Mus musculus OX=10090 GN=Prc | 9554,97 | 1,35 |
| 440 | Q02819 | NUCB1_MOUSE Nucleobindin-1 OS=Mus musculus OX=10090 GN=Nu | 619,61 | 1,35 |
| 441 | O88947 | FA10_MOUSE Coagulation factor X OS=Mus musculus OX=10090 GN= | 567,38 | 1,34 |
| 442 | Q9DBG6 | RPN2_MOUSE Dolichyl-diphosphooligosaccharide--protein glycosyltra | 1052,2 | 1,34 |
| 443 | Q6PIE5 | AT1A2_MOUSE Sodium/potassium-transporting ATPase subunit alpha | 547,99 | 1,34 |
| 444 | P05202 | AATM_MOUSE Aspartate aminotransferase_ mitochondrial OS=Mus r | 4836,67 | 1,34 |
| 445 | Q91YQ5 | RPN1_MOUSE Dolichyl-diphosphooligosaccharide--protein glycosyltra | 1374,59 | 1,32 |
| 446 | Q62465 | VAT1_MOUSE Synaptic vesicle membrane protein VAT-1 homolog OS: | 1916,5 | 1,32 |
| 447 | P48678 | LMNA_MOUSE Prelamin-A/C OS=Mus musculus OX=10090 GN=Lmna | 1851,3 | 1,32 |
| 448 | O08709 | PRDX6_MOUSE Peroxiredoxin-6 OS=Mus musculus OX=10090 GN=Prc | 2013,95 | 1,32 |
| 449 | O35658 | C1QBP_MOUSE Complement component 1 Q subcomponent-binding | 616,51 | 1,32 |
| 450 | Q9R112 | SQOR_MOUSE Sulfide:quinone oxidoreductase_ mitochondrial OS=M | 734,35 | 1,32 |
| 451 | Q91YR9 | PTGR1_MOUSE Prostaglandin reductase 1 OS=Mus musculus OX=100! | 914,48 | 1,31 |
| 452 | P67778 | PHB_MOUSE Prohibitin OS=Mus musculus OX=10090 GN=Phb PE=1 S\ | 8833,21 | 1,31 |
| 453 | Q9D1R9 | RL34_MOUSE 60S ribosomal protein L34 OS=Mus musculus OX=1009( | 1372,15 | 1,31 |
| 454 | Q9CQX2 | CYB5B_MOUSE Cytochrome b5 type B OS=Mus musculus OX=10090 G | 1081,16 | 1,31 |
| 455 | Q9WVJ2 | PSD13_MOUSE 26S proteasome non-ATPase regulatory subunit 13 OS | 229,01 | 1,31 |
| 456 | Q9CZD3 | GARS_MOUSE Glycine--tRNA ligase OS=Mus musculus OX=10090 GN= | 425,55 | 1,30 |
| 457 | O54734 | OST48_MOUSE Dolichyl-diphosphooligosaccharide--protein glycosyltr | 1282,42 | 1,30 |
| 458 | Q9WV27 | AT1A4_MOUSE Sodium/potassium-transporting ATPase subunit alpha | 376,91 | 1,30 |
| 459 | O55143 | AT2A2_MOUSE Sarcoplasmic/endoplasmic reticulum calcium ATPase | 355,65 | 1,30 |
| 460 | Q64310 | SURF4_MOUSE Surfeit locus protein 4 OS=Mus musculus OX=10090 G | 1189,73 | 1,30 |
| 461 | P51859 | HDGF_MOUSE Hepatoma-derived growth factor OS=Mus musculus O) | 528,63 | 1,30 |
| 462 | Q9Z1Q9 | SYVC_MOUSE Valine--tRNA ligase OS=Mus musculus OX=10090 GN=V | 398,63 | 1,30 |
| 463 | P10518 | HEM2_MOUSE Delta-aminolevulinic acid dehydratase OS=Mus muscu | 698,75 | 0,70 |
| 464 | Q9ESK9 | RBCC1_MOUSE RB1-inducible coiled-coil protein 1 OS=Mus musculus | 466,34 | 0,70 |
| 465 | P62835 | RAP1A_MOUSE Ras-related protein Rap-1A OS=Mus musculus OX=10( | 789,61 | 0,70 |
| 466 | P16125 | LDHB_MOUSE L-lactate dehydrogenase B chain OS=Mus musculus OX | 8634,05 | 0,70 |
| 467 | P26645 | MARCS_MOUSE Myristoylated alanine-rich C-kinase substrate OS=Mu | 627,21 | 0,69 |
| 468 | Q9JM76 | ARPC3_MOUSE Actin-related protein 2/3 complex subunit 3 OS=Mus | 1931,37 | 0,69 |
| 469 | P47757 | CAPZB_MOUSE F-actin-capping protein subunit beta OS=Mus muscul | 820,83 | 0,69 |
| 470 | Q9WUL7 | ARL3_MOUSE ADP-ribosylation factor-like protein 3 OS=Mus musculu | 1056,98 | 0,68 |
| 471 | Q5DTY9 | KCD16_MOUSE BTB/POZ domain-containing protein KCTD16 OS=Mus | 438,39 | 0,68 |
| 472 | P48771 | CX7A2_MOUSE Cytochrome c oxidase subunit 7A2_ mitochondrial OS | 3136,07 | 0,68 |
| 473 | P0DP28 | CALM3_MOUSE Calmodulin-3 OS=Mus musculus OX=10090 GN=Calm | 1882,43 | 0,68 |
| 474 | Q99JI6 | RAP1B_MOUSE Ras-related protein Rap-1b OS=Mus musculus OX=10( | 955,35 | 0,68 |
| 475 | Q8BH95 | ECHM_MOUSE Enoyl-CoA hydratase_ mitochondrial OS=Mus musculu | 941,57 | 0,68 |
| 476 | Q9D312 | K1C20_MOUSE Keratin_ type I cytoskeletal 20 OS=Mus musculus OX= | 1107,62 | 0,68 |
| 477 | P39749 | FEN1_MOUSE Flap endonuclease 1 OS=Mus musculus OX=10090 GN= | 419,21 | 0,67 |
| 478 | P0DP27 | CALM2_MOUSE Calmodulin-2 OS=Mus musculus OX=10090 GN=Calm | 1882,43 | 0,67 |
| 479 | P0DP26 | CALM1_MOUSE Calmodulin-1 OS=Mus musculus OX=10090 GN=Calm | 1882,43 | 0,67 |
| 480 | Q8BWT1 | THIM_MOUSE 3-ketoacyl-CoA thiolase_ mitochondrial OS=Mus muscu | 507,2 | 0,66 |
| 481 | P29699 | FETUA_MOUSE Alpha-2-HS-glycoprotein OS=Mus musculus OX=1009( | 1750,39 | 0,66 |
| 482 | Q6IFX2 | K1C42_MOUSE Keratin_ type I cytoskeletal 42 OS=Mus musculus OX= | 1358,45 | 0,66 |
| 483 | Q9QUM9 | PSA6_MOUSE Proteasome subunit alpha type-6 OS=Mus musculus O) | 1540,65 | 0,66 |
| 484 | Q80UG5 | SEPT9_MOUSE Septin-9 OS=Mus musculus OX=10090 GN=Sept9 PE=1 | 529,57 | 0,64 |
| 485 | P19536 | COX5B_MOUSE Cytochrome c oxidase subunit 5B_ mitochondrial OS= | 1879,63 | 0,64 |

| | | | | |
|---|---|---|---|---|
| 486 | P25085 | IL1RA_MOUSE Interleukin-1 receptor antagonist protein OS=Mus mus | 1715,99 | 0,64 |
| 487 | Q8CI43 | MYL6B_MOUSE Myosin light chain 6B OS=Mus musculus OX=10090 G | 1542,77 | 0,64 |
| 488 | Q99KE1 | MAOM_MOUSE NAD-dependent malic enzyme_ mitochondrial OS=M | 3681,11 | 0,64 |
| 489 | Q9Z1Z0 | USO1_MOUSE General vesicular transport factor p115 OS=Mus muscu | 213,34 | 0,63 |
| 490 | Q9WVA4 | TAGL2_MOUSE Transgelin-2 OS=Mus musculus OX=10090 GN=Tagln2 | 5047,33 | 0,63 |
| 491 | Q8BMF3 | MAON_MOUSE NADP-dependent malic enzyme_ mitochondrial OS=M | 368,88 | 0,62 |
| 492 | P61089 | UBE2N_MOUSE Ubiquitin-conjugating enzyme E2 N OS=Mus musculu | 1787,86 | 0,62 |
| 493 | P20918 | PLMN_MOUSE Plasminogen OS=Mus musculus OX=10090 GN=Plg PE= | 277,5 | 0,61 |
| 494 | P16045 | LEG1_MOUSE Galectin-1 OS=Mus musculus OX=10090 GN=Lgals1 PE= | 27973,26 | 0,61 |
| 495 | Q9JJU8 | SH3L1_MOUSE SH3 domain-binding glutamic acid-rich-like protein OS | 2113,09 | 0,61 |
| 496 | P29416 | HEXA_MOUSE Beta-hexosaminidase subunit alpha OS=Mus musculus | 452,88 | 0,59 |
| 497 | O89086 | RBM3_MOUSE RNA-binding protein 3 OS=Mus musculus OX=10090 G | 1041,62 | 0,59 |
| 498 | P11103 | PARP1_MOUSE Poly [ADP-ribose] polymerase 1 OS=Mus musculus OX | 796,79 | 0,59 |
| 499 | Q8VED5 | K2C79_MOUSE Keratin_ type II cytoskeletal 79 OS=Mus musculus OX= | 214,19 | 0,58 |
| 500 | P56391 | CX6B1_MOUSE Cytochrome c oxidase subunit 6B1 OS=Mus musculus | 1733,67 | 0,57 |
| 501 | Q61425 | HCDH_MOUSE Hydroxyacyl-coenzyme A dehydrogenase_ mitochondr | 441,71 | 0,56 |
| 502 | Q6A068 | CDC5L_MOUSE Cell division cycle 5-like protein OS=Mus musculus OX | 803,45 | 0,56 |
| 503 | Q05816 | FABP5_MOUSE Fatty acid-binding protein_ epidermal OS=Mus muscu | 656,65 | 0,55 |
| 504 | P50543 | S10AB_MOUSE Protein S100-A11 OS=Mus musculus OX=10090 GN=S1 | 22532,69 | 0,53 |
| 505 | O54974 | LEG7_MOUSE Galectin-7 OS=Mus musculus OX=10090 GN=Lgals7 PE= | 4091,43 | 0,51 |
| 506 | O08739 | AMPD3_MOUSE AMP deaminase 3 OS=Mus musculus OX=10090 GN= | 707,53 | 0,50 |
| 507 | P02089 | HBB2_MOUSE Hemoglobin subunit beta-2 OS=Mus musculus OX=100 | 1416,55 | 0,48 |
| 508 | P02088 | HBB1_MOUSE Hemoglobin subunit beta-1 OS=Mus musculus OX=100 | 1416,55 | 0,48 |
| 509 | P48964 | MPIP1_MOUSE M-phase inducer phosphatase 1 OS=Mus musculus OX | 310,74 | 0,47 |
| 510 | Q64FW2 | RETST_MOUSE All-trans-retinol 13_14-reductase OS=Mus musculus O | 804,37 | 0,44 |
| 511 | P02104 | HBE_MOUSE Hemoglobin subunit epsilon-Y2 OS=Mus musculus OX=1 | 624,37 | 0,44 |
| 512 | Q5SVR0 | TBC9B_MOUSE TBC1 domain family member 9B OS=Mus musculus OX | 560,74 | 0,40 |
| 513 | P97447 | FHL1_MOUSE Four and a half LIM domains protein 1 OS=Mus muscul | 341 | 0,40 |
| 514 | Q3UKK2 | CEAM5_MOUSE Carcinoembryonic antigen-related cell adhesion mol | 810,53 | 0,40 |
| 515 | P70441 | NHRF1_MOUSE Na(+)/H(+) exchange regulatory cofactor NHE-RF1 OS | 800,1 | 0,39 |
| 516 | P07724 | ALBU_MOUSE Serum albumin OS=Mus musculus OX=10090 GN=Alb P | 5660,6 | 0,38 |
| 517 | Q920F6 | SMC1B_MOUSE Structural maintenance of chromosomes protein 1B ( | 793,69 | 0,38 |
| 518 | Q8VC30 | TKFC_MOUSE Triokinase/FMN cyclase OS=Mus musculus OX=10090 G | 317,93 | 0,37 |
| 519 | Q99LI7 | CSTF3_MOUSE Cleavage stimulation factor subunit 3 OS=Mus muscul | 672,89 | 0,37 |
| 520 | Q9CUL5 | DRC11_MOUSE Dynein regulatory complex protein 11 OS=Mus muscu | 476,11 | 0,37 |
| 521 | Q8BGY2 | IF5A2_MOUSE Eukaryotic translation initiation factor 5A-2 OS=Mus m | 1327,52 | 0,36 |
| 522 | Q91VN4 | MIC25_MOUSE MICOS complex subunit Mic25 OS=Mus musculus OX= | 778,78 | 0,36 |
| 523 | Q9CQF9 | PCYOX_MOUSE Prenylcysteine oxidase OS=Mus musculus OX=10090 ( | 232,52 | 0,35 |
| 524 | Q8BI22 | CE128_MOUSE Centrosomal protein of 128 kDa OS=Mus musculus OX | 1081,41 | 0,34 |
| 525 | Q5SNZ0 | GRDN_MOUSE Girdin OS=Mus musculus OX=10090 GN=Ccdc88a PE=1 | 819,49 | 0,34 |
| 526 | Q6PCM1 | KDM3A_MOUSE Lysine-specific demethylase 3A OS=Mus musculus OX | 834,53 | 0,33 |
| 527 | Q8C0T5 | SI1L1_MOUSE Signal-induced proliferation-associated 1-like protein 1 | 799,2 | 0,33 |
| 528 | Q9CQ75 | NDUA2_MOUSE NADH dehydrogenase [ubiquinone] 1 alpha subcomp | 666,66 | 0,33 |
| 529 | P21278 | GNA11_MOUSE Guanine nucleotide-binding protein subunit alpha-11 | 241,36 | 0,32 |
| 530 | A2AJI0 | MA7D1_MOUSE MAP7 domain-containing protein 1 OS=Mus musculu | 577,95 | 0,32 |
| 531 | Q80V31 | CE104_MOUSE Centrosomal protein of 104 kDa OS=Mus musculus OX | 852,64 | 0,32 |
| 532 | P28738 | KIF5C_MOUSE Kinesin heavy chain isoform 5C OS=Mus musculus OX= | 861,84 | 0,31 |
| 533 | Q0VGT4 | ZGRF1_MOUSE Protein ZGRF1 OS=Mus musculus OX=10090 GN=Zgrf1 | 800,24 | 0,30 |
| 534 | P59759 | MKL2_MOUSE MKL/myocardin-like protein 2 OS=Mus musculus OX=1 | 910,82 | 0,30 |
| 535 | P01027 | CO3_MOUSE Complement C3 OS=Mus musculus OX=10090 GN=C3 PE | 522,19 | 0,28 |
| 536 | Q6A009 | LTN1_MOUSE E3 ubiquitin-protein ligase listerin OS=Mus musculus O | 795,67 | 0,26 |
| 537 | Q8R1F1 | NIBL1_MOUSE Niban-like protein 1 OS=Mus musculus OX=10090 GN= | 254,65 | 0,26 |
| 538 | P01942 | HBA_MOUSE Hemoglobin subunit alpha OS=Mus musculus OX=10090 | 1520,14 | 0,21 |
| 539 | P08207 | S10AA_MOUSE Protein S100-A10 OS=Mus musculus OX=10090 GN=S1 | 957,81 | 0,21 |
| 540 | Q6GQT1 | A2MG_MOUSE Alpha-2-macroglobulin-P OS=Mus musculus OX=1009( | 601,91 | 0,20 |

| | | | | |
|---|---|---|---|---|
| 541 | Q61043 | NIN_MOUSE Ninein OS=Mus musculus OX=10090 GN=Nin PE=1 SV=4 | 337,22 | 0,19 |
| 542 | Q9EP71 | RAI14_MOUSE Ankycorbin OS=Mus musculus OX=10090 GN=Rai14 PE | 256,89 | 0,17 |
| 543 | Q6AXC6 | DDX11_MOUSE ATP-dependent DNA helicase DDX11 OS=Mus muscul | 258,99 | 0,17 |
| 544 | E9PX95 | ABCAH_MOUSE ATP-binding cassette sub-family A member 17 OS=Mu | 217,37 | 0,16 |
| 545 | Q32NZ6 | TMC5_MOUSE Transmembrane channel-like protein 5 OS=Mus muscu | 220,3 | 0,16 |
| 546 | Q3UWM4 | KDM7A_MOUSE Lysine-specific demethylase 7A OS=Mus musculus OX | 270,38 | 0,16 |
| 547 | Q921I1 | TRFE_MOUSE Serotransferrin OS=Mus musculus OX=10090 GN=Tf PE= | 1006,34 | 0,16 |
| 548 | Q9Z1K7 | APCL_MOUSE Adenomatous polyposis coli protein 2 OS=Mus musculu | 231,72 | 0,16 |
| 549 | Q9R269 | PEPL_MOUSE Periplakin OS=Mus musculus OX=10090 GN=Ppl PE=1 SV | 118,22 | 0,16 |
| 550 | O08648 | M3K4_MOUSE Mitogen-activated protein kinase kinase kinase 4 OS=M | 225,77 | 0,16 |
| 551 | Q6A0A9 | F120A_MOUSE Constitutive coactivator of PPAR-gamma-like protein 1 | 255,66 | 0,1 |
| 552 | Q91W10 | S39A8_MOUSE Zinc transporter ZIP8 OS=Mus musculus OX=10090 GN | 862,18 | 0,1 |
| 553 | Q9WU01 | KHDR2_MOUSE KH domain-containing_ RNA-binding_ signal transduc | 258,78 | 0,1 |
| 554 | Q8VBT0 | TMX1_MOUSE Thioredoxin-related transmembrane protein 1 OS=Mu | 276,74 | 0,1 |
| 555 | Q9CQI6 | COTL1_MOUSE Coactosin-like protein OS=Mus musculus OX=10090 G | 293,26 | 0,1 |
| 556 | P31315 | GSX1_MOUSE GS homeobox 1 OS=Mus musculus OX=10090 GN=Gsx1 | 281,41 | 0,1 |
| 557 | P23780 | BGAL_MOUSE Beta-galactosidase OS=Mus musculus OX=10090 GN=G | 212,28 | 0,1 |
| 558 | Q9CQH3 | NDUB5_MOUSE NADH dehydrogenase [ubiquinone] 1 beta subcompl | 239,22 | 0,1 |
| 559 | O35855 | BCAT2_MOUSE Branched-chain-amino-acid aminotransferase_ mitocl | 229,81 | 0,1 |
| 560 | Q61136 | PRP4B_MOUSE Serine/threonine-protein kinase PRP4 homolog OS=M | 226,52 | 0,1 |
| 561 | Q91Z31 | PTBP2_MOUSE Polypyrimidine tract-binding protein 2 OS=Mus muscu | 316,43 | 0,1 |
| 562 | P48455 | PP2BC_MOUSE Serine/threonine-protein phosphatase 2B catalytic sul | 339,63 | 0,1 |
| 563 | Q8K1S6 | SPIR2_MOUSE Protein spire homolog 2 OS=Mus musculus OX=10090 | 412,52 | 0,1 |
| 564 | Q9CQF3 | CPSF5_MOUSE Cleavage and polyadenylation specificity factor subuni | 319,05 | 0,1 |
| 565 | Q9D5K4 | S1PBP_MOUSE S100P-binding protein OS=Mus musculus OX=10090 G | 214,35 | 0,1 |
| 566 | Q61107 | GBP4_MOUSE Guanylate-binding protein 4 OS=Mus musculus OX=10( | 249,99 | 0,1 |
| 567 | Q9DBC7 | KAP0_MOUSE cAMP-dependent protein kinase type I-alpha regulator | 264,82 | 0,1 |
| 568 | Q99JW4 | LIMS1_MOUSE LIM and senescent cell antigen-like-containing domair | 313,63 | 0,1 |
| 569 | P48774 | GSTM5_MOUSE Glutathione S-transferase Mu 5 OS=Mus musculus O) | 290,61 | 0,1 |
| 570 | Q91YM2 | RHG35_MOUSE Rho GTPase-activating protein 35 OS=Mus musculus ( | 332,53 | 0,1 |
| 571 | Q9Z172 | SUMO3_MOUSE Small ubiquitin-related modifier 3 OS=Mus musculus | 2260,07 | 0,1 |
| 572 | Q9Z0U1 | ZO2_MOUSE Tight junction protein ZO-2 OS=Mus musculus OX=1009( | 156,57 | 0,1 |
| 573 | Q3UPC7 | K0825_MOUSE Uncharacterized protein KIAA0825 homolog OS=Mus r | 225,13 | 0,1 |
| 574 | Q3LAC4 | PREX2_MOUSE Phosphatidylinositol 3_4_5-trisphosphate-dependent | 312,6 | 0,1 |
| 575 | O55029 | COPB2_MOUSE Coatomer subunit beta' OS=Mus musculus OX=10090 | 328,47 | 0,1 |
| 576 | Q3UIU2 | NDUB6_MOUSE NADH dehydrogenase [ubiquinone] 1 beta subcompl | 390,98 | 0,1 |
| 577 | Q6A044 | F1891_MOUSE Protein FAM189A1 OS=Mus musculus OX=10090 GN=I | 230,35 | 0,1 |
| 578 | E9Q7D5 | ARHG5_MOUSE Rho guanine nucleotide exchange factor 5 OS=Mus m | 252,23 | 0,1 |
| 579 | Q9JJY4 | DDX20_MOUSE Probable ATP-dependent RNA helicase DDX20 OS=Mu | 219,82 | 0,1 |
| 580 | O08808 | DIAP1_MOUSE Protein diaphanous homolog 1 OS=Mus musculus OX= | 213,7 | 0,1 |
| 581 | P61226 | RAP2B_MOUSE Ras-related protein Rap-2b OS=Mus musculus OX=10( | 224,41 | 0,1 |
| 582 | O89017 | LGMN_MOUSE Legumain OS=Mus musculus OX=10090 GN=Lgmn PE= | 207,6 | 0,1 |
| 583 | P62305 | RUXE_MOUSE Small nuclear ribonucleoprotein E OS=Mus musculus O | 565,63 | 0,1 |
| 584 | G5E8P0 | GCP6_MOUSE Gamma-tubulin complex component 6 OS=Mus muscu | 207,54 | 0,1 |
| 585 | Q920Q2 | REV1_MOUSE DNA repair protein REV1 OS=Mus musculus OX=10090 | 263,04 | 0,1 |
| 586 | Q9DAW9 | CNN3_MOUSE Calponin-3 OS=Mus musculus OX=10090 GN=Cnn3 PE= | 209,67 | 0,1 |
| 587 | P33175 | KIF5A_MOUSE Kinesin heavy chain isoform 5A OS=Mus musculus OX= | 350,31 | 0,1 |
| 588 | Q61704 | ITIH3_MOUSE Inter-alpha-trypsin inhibitor heavy chain H3 OS=Mus m | 224,87 | 0,1 |
| 589 | Q61703 | ITIH2_MOUSE Inter-alpha-trypsin inhibitor heavy chain H2 OS=Mus m | 453,98 | 0,1 |
| 590 | Q9JJT2 | GFRA4_MOUSE GDNF family receptor alpha-4 OS=Mus musculus OX= | 250,8 | 0,1 |
| 591 | Q9D8B3 | CHM4B_MOUSE Charged multivesicular body protein 4b OS=Mus mus | 455,63 | 0,1 |
| 592 | O35166 | GOSR2_MOUSE Golgi SNAP receptor complex member 2 OS=Mus mus | 327,8 | 0,1 |
| 593 | Q6PGH1 | BUD31_MOUSE Protein BUD31 homolog OS=Mus musculus OX=1009( | 747,26 | 0,1 |
| 594 | Q9CPT4 | MYDGF_MOUSE Myeloid-derived growth factor OS=Mus musculus OX | 340,94 | 0,1 |
| 595 | Q3UYK3 | TBCD9_MOUSE TBC1 domain family member 9 OS=Mus musculus OX= | 566,48 | 0,1 |

| | | | | |
|---|---|---|---|---|
| 596 | Q9DAS9 | GBG12_MOUSE Guanine nucleotide-binding protein G(I)/G(S)/G(O) su | 512,12 | 0,1 |
| 597 | Q80ZJ1 | RAP2A_MOUSE Ras-related protein Rap-2a OS=Mus musculus OX=100 | 258,96 | 0,1 |
| 598 | Q8C6E0 | CFA36_MOUSE Cilia- and flagella-associated protein 36 OS=Mus musc | 782,71 | 0,1 |
| 599 | A2AG50 | MA7D2_MOUSE MAP7 domain-containing protein 2 OS=Mus musculu | 122,37 | 0,1 |
| 600 | Q9Z0H8 | CLIP2_MOUSE CAP-Gly domain-containing linker protein 2 OS=Mus m | 257,43 | 0,1 |
| 601 | O88559 | MEN1_MOUSE Menin OS=Mus musculus OX=10090 GN=Men1 PE=1 S | 199,89 | 0,1 |
| 602 | P52503 | NDUS6_MOUSE NADH dehydrogenase [ubiquinone] iron-sulfur protei | 494,4 | 0,1 |
| 603 | Q8BL06 | UBP54_MOUSE Inactive ubiquitin carboxyl-terminal hydrolase 54 OS= | 436 | 0,1 |
| 604 | Q8CIG3 | KDM1B_MOUSE Lysine-specific histone demethylase 1B OS=Mus mus | 321,59 | 0,1 |
| 605 | Q9CZB0 | C560_MOUSE Succinate dehydrogenase cytochrome b560 subunit_ m | 275,48 | 0,1 |
| 606 | Q2XU92 | ACBG2_MOUSE Long-chain-fatty-acid--CoA ligase ACSBG2 OS=Mus mu | 227,93 | 0,1 |
| 607 | P10922 | H10_MOUSE Histone H1.0 OS=Mus musculus OX=10090 GN=H1f0 PE= | 296,96 | 0,1 |
| 608 | B2RRF6 | Z518A_MOUSE Zinc finger protein 518A OS=Mus musculus OX=10090 | 358,11 | 0,1 |
| 609 | Q8R4I4 | TF2AY_MOUSE TFIIA-alpha and beta-like factor OS=Mus musculus OX | 216,34 | 0,1 |
| 610 | Q62165 | DAG1_MOUSE Dystroglycan OS=Mus musculus OX=10090 GN=Dag1 P | 283,39 | 0,1 |
| 611 | Q8BXC6 | COMD2_MOUSE COMM domain-containing protein 2 OS=Mus muscu | 262,48 | 0,1 |
| 612 | P06467 | HBAZ_MOUSE Hemoglobin subunit zeta OS=Mus musculus OX=10090 | 553,99 | 0,1 |
| 613 | Q64302 | T4S1_MOUSE Transmembrane 4 L6 family member 1 OS=Mus muscul | 486,37 | 0,1 |
| 614 | Q91XS1 | MTMR4_MOUSE Myotubularin-related protein 4 OS=Mus musculus O | 216,61 | 0,1 |
| 615 | Q9ERE8 | TLRN1_MOUSE Talin rod domain-containing protein 1 OS=Mus muscu | 283,06 | 0,1 |
| 616 | Q9CYZ2 | TPD54_MOUSE Tumor protein D54 OS=Mus musculus OX=10090 GN= | 331,1 | 0,1 |
| 617 | Q9WVQ5 | MTNB_MOUSE Methylthioribulose-1-phosphate dehydratase OS=Mus | 278,03 | 0,1 |
| 618 | Q8BU85 | MSRB3_MOUSE Methionine-R-sulfoxide reductase B3_ mitochondrial | 237,76 | 0,1 |
| 619 | Q62468 | VILI_MOUSE Villin-1 OS=Mus musculus OX=10090 GN=Vil1 PE=1 SV=3 | 347,77 | 0,1 |
| 620 | Q61011 | GBB3_MOUSE Guanine nucleotide-binding protein G(I)/G(S)/G(T) sub | 288,14 | 0,1 |
| 621 | O35723 | DNJB3_MOUSE DnaJ homolog subfamily B member 3 OS=Mus muscul | 252,6 | 0,1 |
| 622 | Q8BR07 | BICD1_MOUSE Protein bicaudal D homolog 1 OS=Mus musculus OX=1 | 292,6 | 0,1 |
| 623 | Q9D7P9 | SPB12_MOUSE Serpin B12 OS=Mus musculus OX=10090 GN=Serpinb1 | 238,44 | 0,1 |
| 624 | Q9CW03 | SMC3_MOUSE Structural maintenance of chromosomes protein 3 OS= | 192,36 | 0,1 |
| 625 | P47226 | TES_MOUSE Testin OS=Mus musculus OX=10090 GN=Tes PE=1 SV=1 | 543,05 | 0,1 |
| 626 | Q99J77 | SIAS_MOUSE Sialic acid synthase OS=Mus musculus OX=10090 GN=Na | 254,48 | 0,1 |
| 627 | Q8BU33 | ILVBL_MOUSE Acetolactate synthase-like protein OS=Mus musculus C | 487,96 | 0,1 |
| 628 | Q8BU31 | RAP2C_MOUSE Ras-related protein Rap-2c OS=Mus musculus OX=100 | 258,96 | 0,1 |
| 629 | Q7TNP2 | 2AAB_MOUSE Serine/threonine-protein phosphatase 2A 65 kDa regul | 633,71 | 0,1 |
| 630 | P97363 | SPTC2_MOUSE Serine palmitoyltransferase 2 OS=Mus musculus OX=1 | 384,65 | 0,1 |
| 631 | Q9WVJ3 | CBPQ_MOUSE Carboxypeptidase Q OS=Mus musculus OX=10090 GN= | 243,95 | 0,1 |
| 632 | Q8C5P7 | TDRP_MOUSE Testis development-related protein OS=Mus musculus | 266,43 | 0,1 |
| 633 | Q3TDQ1 | STT3B_MOUSE Dolichyl-diphosphooligosaccharide--protein glycosyltr | 233,61 | 0,1 |
| 634 | Q78ZA7 | NP1L4_MOUSE Nucleosome assembly protein 1-like 4 OS=Mus muscu | 214,77 | 0,1 |
| 635 | Q9R1Q8 | TAGL3_MOUSE Transgelin-3 OS=Mus musculus OX=10090 GN=Tagln3 | 257,58 | 0,1 |
| 636 | Q9R226 | KHDR3_MOUSE KH domain-containing_ RNA-binding_ signal transduc | 285,02 | 0,1 |
| 637 | P63321 | RALA_MOUSE Ras-related protein Ral-A OS=Mus musculus OX=10090 | 314,84 | 0,1 |
| 638 | P05977 | MYL1_MOUSE Myosin light chain 1/3_ skeletal muscle isoform OS=Mu | 582,85 | 0,1 |
| 639 | Q8K419 | LEG4_MOUSE Galectin-4 OS=Mus musculus OX=10090 GN=Lgals4 PE= | 219,66 | 0,1 |
| 640 | O08716 | FABP9_MOUSE Fatty acid-binding protein 9 OS=Mus musculus OX=10 | 451,6 | 0,1 |
| 641 | P48972 | MYBB_MOUSE Myb-related protein B OS=Mus musculus OX=10090 G | 235,06 | 0,1 |
| 642 | P35282 | RAB21_MOUSE Ras-related protein Rab-21 OS=Mus musculus OX=100 | 244,63 | 0,1 |
| 643 | Q8BWS5 | GRIN3_MOUSE G protein-regulated inducer of neurite outgrowth 3 O | 265,84 | 0,1 |
| 644 | Q8BJY1 | PSMD5_MOUSE 26S proteasome non-ATPase regulatory subunit 5 OS | 291,55 | 0,1 |
| 645 | Q8R480 | NUP85_MOUSE Nuclear pore complex protein Nup85 OS=Mus muscu | 311,74 | 0,1 |
| 646 | Q5SUF2 | LC7L3_MOUSE Luc7-like protein 3 OS=Mus musculus OX=10090 GN=L | 204,9 | 0,1 |
| 647 | Q9R1M5 | NALP5_MOUSE NACHT_ LRR and PYD domains-containing protein 5 O | 189,19 | 0,1 |
| 648 | Q91XD2 | LIMS2_MOUSE LIM and senescent cell antigen-like-containing domair | 253,99 | 0,1 |
| 649 | Q8BX17 | GEMI5_MOUSE Gem-associated protein 5 OS=Mus musculus OX=1009 | 210,63 | 0,1 |
| 650 | P53569 | CEBPZ_MOUSE CCAAT/enhancer-binding protein zeta OS=Mus muscu | 275,18 | 0,1 |

| | | | | |
|---|---|---|---|---|
| 651 | Q7TNH6 | NPHP3_MOUSE Nephrocystin-3 OS=Mus musculus OX=10090 GN=Npl | 271 | 0,1 |
| 652 | Q99M31 | HSP7E_MOUSE Heat shock 70 kDa protein 14 OS=Mus musculus OX=1 | 333,91 | 0,1 |
| 653 | Q3UKU4 | FA83F_MOUSE Protein FAM83F OS=Mus musculus OX=10090 GN=Far | 198,86 | 0,1 |
| 654 | Q9D0W5 | PPIL1_MOUSE Peptidyl-prolyl cis-trans isomerase-like 1 OS=Mus musc | 324,59 | 0,1 |
| 655 | P62892 | RL39_MOUSE 60S ribosomal protein L39 OS=Mus musculus OX=1009( | 6785,47 | 0,1 |
| 656 | Q9WVB0 | RBPMS_MOUSE RNA-binding protein with multiple splicing OS=Mus n | 212,06 | 0,1 |
| 657 | Q8BK64 | AHSA1_MOUSE Activator of 90 kDa heat shock protein ATPase homol | 423,06 | 0,1 |
| 658 | Q8K3J1 | NDUS8_MOUSE NADH dehydrogenase [ubiquinone] iron-sulfur protei | 445,06 | 0,1 |
| 659 | P11985 | TC1D3_MOUSE Tctex1 domain-containing protein 3 OS=Mus musculu | 475,38 | 0,1 |
| 660 | Q810Y8 | PRAL7_MOUSE Preferentially expressed antigen in melanoma-like prc | 215,2 | 0,1 |
| 661 | P35585 | AP1M1_MOUSE AP-1 complex subunit mu-1 OS=Mus musculus OX=1( | 233,07 | 0,1 |
| 662 | Q8K0C8 | COX19_MOUSE Cytochrome c oxidase assembly protein COX19 OS=M | 225,83 | 0,1 |
| 663 | Q9DCZ1 | GMPR1_MOUSE GMP reductase 1 OS=Mus musculus OX=10090 GN=( | 265,9 | 0,1 |
| 664 | Q9Z315 | SNUT1_MOUSE U4/U6.U5 tri-snRNP-associated protein 1 OS=Mus mu | 288,06 | 0,1 |
| 665 | O70591 | PFD2_MOUSE Prefoldin subunit 2 OS=Mus musculus OX=10090 GN=P | 294,93 | 0,1 |
| 666 | Q9DA37 | SAMD8_MOUSE Sphingomyelin synthase-related protein 1 OS=Mus m | 295,99 | 0,1 |
| 667 | Q8R404 | MIC13_MOUSE MICOS complex subunit MIC13 OS=Mus musculus OX= | 275,82 | 0,1 |
| 668 | Q6P9L4 | UBP49_MOUSE Ubiquitin carboxyl-terminal hydrolase 49 OS=Mus mu | 281,69 | 0,1 |
| 669 | Q9DCV7 | K2C7_MOUSE Keratin_ type II cytoskeletal 7 OS=Mus musculus OX=1C | 287,71 | 0,1 |
| 670 | Q3UEB3 | PUF60_MOUSE Poly(U)-binding-splicing factor PUF60 OS=Mus muscul | 254,43 | 0,1 |
| 671 | Q6PFD6 | KI18B_MOUSE Kinesin-like protein KIF18B OS=Mus musculus OX=100! | 311,2 | 0,1 |
| 672 | Q6PFD5 | DLGP3_MOUSE Disks large-associated protein 3 OS=Mus musculus OX | 236,05 | 0,1 |
| 673 | P32261 | ANT3_MOUSE Antithrombin-III OS=Mus musculus OX=10090 GN=Serp | 748,76 | 0,1 |
| 674 | Q8BWG8 | ARRB1_MOUSE Beta-arrestin-1 OS=Mus musculus OX=10090 GN=Arrk | 334,66 | 0,1 |
| 675 | Q9D3U0 | PUS10_MOUSE Putative tRNA pseudouridine synthase Pus10 OS=Mus | 300,97 | 0,1 |
| 676 | Q9DCT2 | NDUS3_MOUSE NADH dehydrogenase [ubiquinone] iron-sulfur protei | 257,67 | 0,1 |
| 677 | Q6P069 | SORCN_MOUSE Sorcin OS=Mus musculus OX=10090 GN=Sri PE=1 SV= | 265,06 | 0,1 |
| 678 | P58389 | PTPA_MOUSE Serine/threonine-protein phosphatase 2A activator OS= | 640,3 | 0,1 |
| 679 | Q9D0M5 | DYL2_MOUSE Dynein light chain 2_ cytoplasmic OS=Mus musculus OX | 259,65 | 0,1 |
| 680 | Q6UJY2 | SL9C1_MOUSE Sodium/hydrogen exchanger 10 OS=Mus musculus OX | 263,98 | 0,1 |
| 681 | Q9Z2H7 | GIPC2_MOUSE PDZ domain-containing protein GIPC2 OS=Mus muscul | 267,98 | 0,1 |
| 682 | Q8BGE5 | FANCM_MOUSE Fanconi anemia group M protein homolog OS=Mus r | 204,74 | 0,1 |
| 683 | Q8BGD8 | COA6_MOUSE Cytochrome c oxidase assembly factor 6 homolog OS=I | 283,06 | 0,1 |
| 684 | Q8K099 | LRIT1_MOUSE Leucine-rich repeat_ immunoglobulin-like domain and | 214,58 | 0,1 |
| 685 | A6X935 | ITIH4_MOUSE Inter alpha-trypsin inhibitor_ heavy chain 4 OS=Mus mu | 281,14 | 0,1 |
| 686 | Q9D3P8 | PLRKT_MOUSE Plasminogen receptor (KT) OS=Mus musculus OX=100! | 256,36 | 0,1 |
| 687 | Q9WV55 | VAPA_MOUSE Vesicle-associated membrane protein-associated prote | 484,73 | 0,1 |
| 688 | P15331 | PERI_MOUSE Peripherin OS=Mus musculus OX=10090 GN=Prph PE=1 | 409,33 | 0,1 |
| 689 | O88735 | MAP7_MOUSE Ensconsin OS=Mus musculus OX=10090 GN=Map7 PE= | 384,39 | 0,1 |
| 690 | P50518 | VATE1_MOUSE V-type proton ATPase subunit E 1 OS=Mus musculus ( | 419,57 | 0,1 |
| 691 | Q8BSY0 | ASPH_MOUSE Aspartyl/asparaginyl beta-hydroxylase OS=Mus muscul | 300,39 | 0,1 |
| 692 | Q9CXZ1 | NDUS4_MOUSE NADH dehydrogenase [ubiquinone] iron-sulfur protei | 712,78 | 0,1 |
| 693 | Q9CXY9 | GPI8_MOUSE GPI-anchor transamidase OS=Mus musculus OX=10090 | 226,33 | 0,1 |
| 694 | Q80UV9 | TAF1_MOUSE Transcription initiation factor TFIID subunit 1 OS=Mus r | 245,66 | 0,1 |
| 695 | P06684 | CO5_MOUSE Complement C5 OS=Mus musculus OX=10090 GN=C5 PE | 242,74 | 0,1 |
| 696 | Q80V62 | FACD2_MOUSE Fanconi anemia group D2 protein homolog OS=Mus n | 471,94 | 0,1 |
| 697 | P63280 | UBC9_MOUSE SUMO-conjugating enzyme UBC9 OS=Mus musculus O) | 364,9 | 0,1 |
| 698 | Q925I1 | ATAD3_MOUSE ATPase family AAA domain-containing protein 3 OS=N | 278,25 | 0,1 |
| 699 | P31786 | ACBP_MOUSE Acyl-CoA-binding protein OS=Mus musculus OX=10090 | 293,17 | 0,1 |
| 700 | P51910 | APOD_MOUSE Apolipoprotein D OS=Mus musculus OX=10090 GN=Ap | 224,16 | 0,1 |
| 701 | Q922B6 | TRAF7_MOUSE E3 ubiquitin-protein ligase TRAF7 OS=Mus musculus C | 333,89 | 0,1 |
| 702 | Q64516 | GLPK_MOUSE Glycerol kinase OS=Mus musculus OX=10090 GN=Gk PE | 216,17 | 0,1 |
| 703 | Q8K010 | OPLA_MOUSE 5-oxoprolinase OS=Mus musculus OX=10090 GN=Oplal | 273,33 | 0,1 |
| 704 | P97298 | PEDF_MOUSE Pigment epithelium-derived factor OS=Mus musculus C | 214,71 | 0,1 |
| 705 | Q8CGY8 | OGT1_MOUSE UDP-N-acetylglucosamine--peptide N-acetylglucosamii | 247,02 | 0,1 |

| | | | | |
|---|---|---|---|---|
| 706 | Q9CXU0 | MED10_MOUSE Mediator of RNA polymerase II transcription subunit | 710,55 | 0,1 |
| 707 | Q8BFZ9 | ERLN2_MOUSE Erlin-2 OS=Mus musculus OX=10090 GN=Erlin2 PE=1 S | 278,3 | 0,1 |
| 708 | P63254 | CRIP1_MOUSE Cysteine-rich protein 1 OS=Mus musculus OX=10090 G | 1167,26 | 0,1 |
| 709 | Q80V24 | VGLL4_MOUSE Transcription cofactor vestigial-like protein 4 OS=Mus | 216,53 | 0,1 |
| 710 | Q91WL5 | CP4CA_MOUSE Cytochrome P450 4A12A OS=Mus musculus OX=1009 | 210,77 | 0,1 |
| 711 | Q62315 | JARD2_MOUSE Protein Jumonji OS=Mus musculus OX=10090 GN=Jari | 192,74 | 0,1 |
| 712 | Q91WK2 | EIF3H_MOUSE Eukaryotic translation initiation factor 3 subunit H OS= | 369,72 | 0,1 |
| 713 | Q8C525 | M21D2_MOUSE Protein MB21D2 OS=Mus musculus OX=10090 GN=M | 357,01 | 0,1 |
| 714 | Q9Z1Z2 | STRAP_MOUSE Serine-threonine kinase receptor-associated protein C | 437,65 | 0,1 |
| 715 | Q7TQ62 | PODN_MOUSE Podocan OS=Mus musculus OX=10090 GN=Podn PE=2 | 307,16 | 0,1 |
| 716 | Q01339 | APOH_MOUSE Beta-2-glycoprotein 1 OS=Mus musculus OX=10090 GN | 211,98 | 0,1 |
| 717 | Q61205 | PA1B3_MOUSE Platelet-activating factor acetylhydrolase IB subunit ga | 610,41 | 0,1 |
| 718 | B2RPU2 | PLHD1_MOUSE Pleckstrin homology domain-containing family D mem | 556,59 | 0,1 |
| 719 | Q9QYB5 | ADDG_MOUSE Gamma-adducin OS=Mus musculus OX=10090 GN=Ad | 479,39 | 0,1 |
| 720 | Q7SIG6 | ASAP2_MOUSE Arf-GAP with SH3 domain_ ANK repeat and PH domai | 435,91 | 0,1 |
| 721 | Q9R0N5 | SYT5_MOUSE Synaptotagmin-5 OS=Mus musculus OX=10090 GN=Syt5 | 260,09 | 0,1 |
| 722 | Q80UK7 | SAS6_MOUSE Spindle assembly abnormal protein 6 homolog OS=Mus | 399,47 | 0,1 |
| 723 | P49945 | FRIL2_MOUSE Ferritin light chain 2 OS=Mus musculus OX=10090 GN= | 433,78 | 0,1 |
| 724 | Q9R0M6 | RAB9A_MOUSE Ras-related protein Rab-9A OS=Mus musculus OX=10 | 243,9 | 0,1 |
| 725 | Q8R010 | AIMP2_MOUSE Aminoacyl tRNA synthase complex-interacting multifu | 301,08 | 0,1 |
| 726 | Q60790 | RASA3_MOUSE Ras GTPase-activating protein 3 OS=Mus musculus OX | 188,9 | 0,1 |
| 727 | Q91WD4 | CG025_MOUSE UPF0415 protein C7orf25 homolog OS=Mus musculus | 287,22 | 0,1 |
| 728 | P86049 | RBM46_MOUSE Probable RNA-binding protein 46 OS=Mus musculus | 225,5 | 0,1 |
| 729 | Q9Z1S3 | GRP1_MOUSE RAS guanyl-releasing protein 1 OS=Mus musculus OX=1 | 340,63 | 0,1 |
| 730 | Q99KP6 | PRP19_MOUSE Pre-mRNA-processing factor 19 OS=Mus musculus OX | 218,85 | 0,1 |
| 731 | A2AQP0 | MYH7B_MOUSE Myosin-7B OS=Mus musculus OX=10090 GN=Myh7b | 92,07 | 0,1 |
| 732 | Q7TMF3 | NDUAC_MOUSE NADH dehydrogenase [ubiquinone] 1 alpha subcomp | 297,56 | 0,1 |
| 733 | P07091 | S10A4_MOUSE Protein S100-A4 OS=Mus musculus OX=10090 GN=S10 | 283,49 | 0,1 |
| 734 | Q60749 | KHDR1_MOUSE KH domain-containing_ RNA-binding_ signal transduc | 410,36 | 0,1 |
| 735 | P28667 | MRP_MOUSE MARCKS-related protein OS=Mus musculus OX=10090 G | 330,5 | 0,1 |
| 736 | Q3TIR3 | RIC8A_MOUSE Synembryn-A OS=Mus musculus OX=10090 GN=Ric8a | 795,76 | 0,1 |
| 737 | Q8JZY2 | COMDA_MOUSE COMM domain-containing protein 10 OS=Mus musc | 265,84 | 0,1 |
| 738 | P16056 | MET_MOUSE Hepatocyte growth factor receptor OS=Mus musculus C | 209,38 | 0,1 |
| 739 | Q3UN70 | MRFL_MOUSE Myelin regulatory factor-like protein OS=Mus musculu | 297,42 | 0,1 |
| 740 | Q8R307 | VPS18_MOUSE Vacuolar protein sorting-associated protein 18 homol | 256,3 | 0,1 |
| 741 | Q5SPW0 | VPS54_MOUSE Vacuolar protein sorting-associated protein 54 OS=Mu | 297,63 | 0,1 |
| 742 | Q9DC70 | NDUS7_MOUSE NADH dehydrogenase [ubiquinone] iron-sulfur protei | 359,55 | 0,1 |
| 743 | Q9QY76 | VAPB_MOUSE Vesicle-associated membrane protein-associated prote | 345,08 | 0,1 |
| 744 | P52651 | RHOX5_MOUSE Homeobox protein Rhox5 OS=Mus musculus OX=100 | 284,63 | 0,1 |
| 745 | Q9JL35 | HMGN5_MOUSE High mobility group nucleosome-binding domain-co | 267,89 | 0,1 |
| 746 | Q9EPK6 | SIL1_MOUSE Nucleotide exchange factor SIL1 OS=Mus musculus OX=1 | 498,1 | 0,1 |
| 747 | Q80XD1 | CHIO_MOUSE Beta-chimaerin OS=Mus musculus OX=10090 GN=Chn2 | 221,8 | 0,1 |
| 748 | O88668 | CREG1_MOUSE Protein CREG1 OS=Mus musculus OX=10090 GN=Creg | 230,17 | 0,1 |
| 749 | P09542 | MYL3_MOUSE Myosin light chain 3 OS=Mus musculus OX=10090 GN= | 342,4 | 0,1 |
| 750 | Q8VII3 | CST14_MOUSE Cystatin-14 OS=Mus musculus OX=10090 GN=Cst14 PE | 242,18 | 0,1 |
| 751 | P61957 | SUMO2_MOUSE Small ubiquitin-related modifier 2 OS=Mus musculus | 2678,73 | 0,1 |
| 752 | Q9D328 | TM35A_MOUSE Transmembrane protein 35A OS=Mus musculus OX=1 | 387,76 | 0,1 |
| 753 | Q9CR25 | DPH2_MOUSE 2-(3-amino-3-carboxypropyl)histidine synthase subunit | 260,39 | 0,1 |
| 754 | O35226 | PSMD4_MOUSE 26S proteasome non-ATPase regulatory subunit 4 OS | 272,34 | 0,1 |
| 755 | Q9WTU6 | MK09_MOUSE Mitogen-activated protein kinase 9 OS=Mus musculus | 221,77 | 0,1 |
| 756 | Q9QUJ7 | ACSL4_MOUSE Long-chain-fatty-acid--CoA ligase 4 OS=Mus musculus | 209,59 | 0,1 |
| 757 | Q8CCX5 | KT222_MOUSE Keratin-like protein KRT222 OS=Mus musculus OX=10 | 662,65 | 0,1 |
| 758 | P24549 | AL1A1_MOUSE Retinal dehydrogenase 1 OS=Mus musculus OX=1009 | 274,12 | 0,1 |
| 759 | Q6PB44 | PTN23_MOUSE Tyrosine-protein phosphatase non-receptor type 23 C | 298,62 | 0,1 |
| 760 | P07310 | KCRM_MOUSE Creatine kinase M-type OS=Mus musculus OX=10090 G | 809,41 | 0,1 |

| | | | | |
|---|---|---|---|---|
| 761 | Q02395 | MTF2_MOUSE Metal-response element-binding transcription factor 2 | 404,19 | 0,1 |
| 762 | Q5MJS3 | FA20C_MOUSE Extracellular serine/threonine protein kinase FAM20C | 290,63 | 0,1 |
