# Supplementary material for: Inhibition of the mTOR pathway and reprogramming of protein synthesis by MDM4 reduce ovarian cancer metastatic properties
Source: Cell Death Dis. 2021 May 29;12(6):558. doi: 10.1038/s41419-021-03828-z (PMC8164635; doi:10.1038/s41419-021-03828-z)
Supplement: Supplementary file 3 — Supplemental Figures [file 41419_2021_3828_MOESM3_ESM.pdf]

Online supplemental material

Supplementary Table S3

Top 8 canonical pathways for SK-OV-3 MCTSs

Canonical Pathway Name	p-value
EIF2 Signaling	3,28E01
Signaling by Rho Family GTPases	1,57E01
RhoGDI Signaling	1,53E01
Glucocorticoid Receptor Signaling	1,53E01
Mitochondrial Dysfunction	1,3E01
mTOR Signaling	1,24E01
Regulation of eIF4 and p70S6K Signaling	1,19E01
Protein Ubiquitination Pathway	1,13E01

Top 8 canonical pathways for ID8 MCTSs

Canonical Pathway Name	p-value
Mitochondrial Dysfunction	1,2E01
Protein Ubiquitination Pathway	9,63E00
Sirtuin Signaling Pathway	9,53E00
tRNA Charging	8,75E00
NRF2-mediated Oxidative Stress Response	8,06E00
EIF2 Signaling	4,48E00
mTOR Signaling	3,4E00
Regulation of eIF4 and p70S6K Signaling	3,32E00

Supplementary figure legends

Figure S1. MDM4 decreases ovarian cancer nodules dissemination and improves overall survival probability. **a**, Western blot (WB) showing MDM4 overexpression in SK-OV-3 ovarian cancer cells. **b,** Representative pictures of peritoneal nodules, black arrows indicate peritoneal nodules. **c**, WB of ID8 murine ovarian cancer cell line as in (a). **d**, Representative pictures of mice with ascites (left panels) and nodules in peritoneum membranes, i.e., diaphragm, mesenterium, and peritoneum. **e**, Correlation of MDM2 expression with OS in 1,656 patients with epithelial ovarian cancer followed for 15y (log-rank test).

Figure S2. Regulation of cell migration by different levels of MDM4. **a**, Left panels: representative micrographs of migration of *Empty Vector* and *Mdm4*-ID8 cells evaluated through wound healing assay. Pictures were taken at time 0 and 18h after the scratch. Scale bars= 200µM. Right panel shows the quantification of 3 independent biological replicates (Mean ±SD, n=3 Student's t-test p<0,0001). **b**, WB analysis of MDM4 levels following silencing of MDM4 by Stealth-RNA in A-2780 ovarian cancer cells. **c**, Left panels: same as in (a) for A-2780. Right panel shows quantification of 3 independent biological replicates (Mean ±SD, n=3, Student's t-test p<0,05).

Figure S3. Cell invasion is impaired by high levels of MDM4. **a**, Left panels: representative micrographs of transwell cell invasion assay through matrigel by *Empty Vector*- or *MDM4-ID8* cells. Right panel shows quantification of 3 independent experiments (Mean ±SD, n=3, Student's t-test p<0,0001); **b,** Representative images of multicellular tumor spheroids (MCTS) formed by *Empty Vector*- or *Mdm4-ID8* after 24 hrs of invasion.

Figure S4. Proteomic analysis of murine MCTSs reveals decreased protein translation in Mdm4 expressing cells. **a**, Venn diagram showing overlap of protein expressed in *Empty Vector*- and *Mdm4*-

ID8 cells. **b**, IPA biofunctional analysis of proteins differently expressed in Mdm4 cells. **c**, Left panel: SunSet assay in *Empty Vector*- and *Mdm4*-ID8 cells: representative WB analysis of puromycin marked protein levels. Right panel: quantification of 3 independent experiments (Mean ±SD, n=3, Student's t-test p<0,0001). **d**, Pathway analysis of mTOR signaling in ID8 cells

Figure S5. Rapamycin treatment is ineffective in MDM4 expressing cells **a**, Scheme of full-length and MDM4 deletion mutants used in SK-OV-3 cells. BD= p53 binding domain, RF= Ring Finger domain, Centr= central region lacking both BD and RF domains. **b**, Representative images of cell migration of SKOV-3 cells Empty Vector and MDM4 using Rapamycin (100nM) or DMSO at T0 and 15h after the scratch.

Video S1. Rapamycin treatment is ineffective in MDM4 expressing cells. Representative images of cell migration of *Empty Vector* and *MDM4*-SK-OV-3 cells using Rapamycin (100nM) or DMSO, 15h after the scratch. Upper panels: *Empty Vector* (left) and *MDM4* (right) SK-OV-3 cells treated with DMSO; lower panels: *Empty Vector* (left) and *MDM4* (right) SK-OV-3 cells treated with Rapamycin (100nM).

a

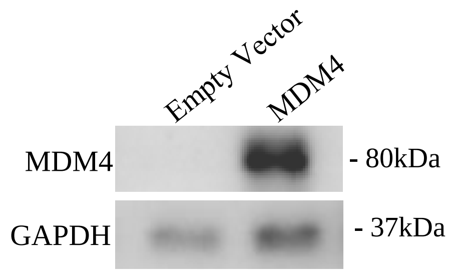
Empty Vector
MDM4
MDM4
- 80kDa
GAPDH
- 37kDa

b

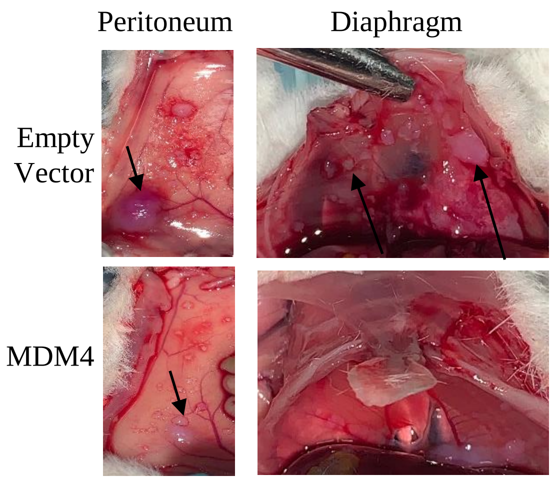
Peritoneum
Diaphragm
Empty Vector
MDM4

c

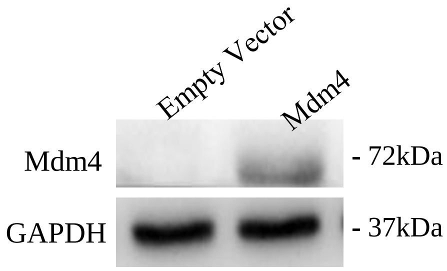
Empty Vector
Mdm4
Mdm4
- 72kDa
GAPDH
- 37kDa

d

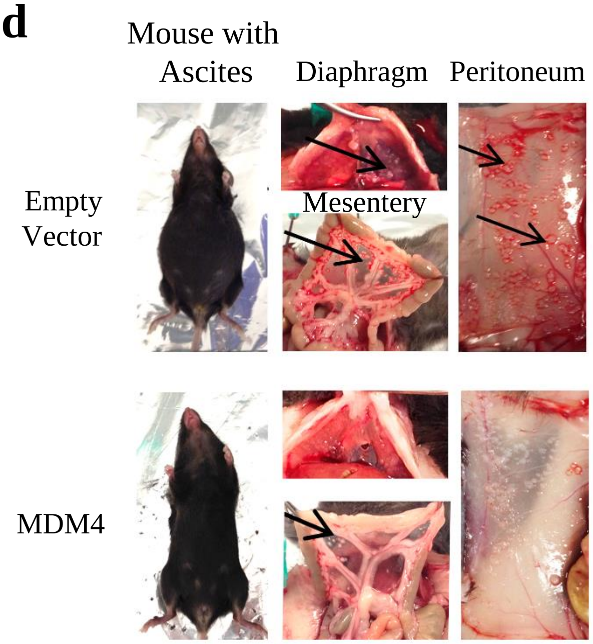
Mouse with Ascites
Diaphragm
Peritoneum
Empty Vector
Mesentery
MDM4

e

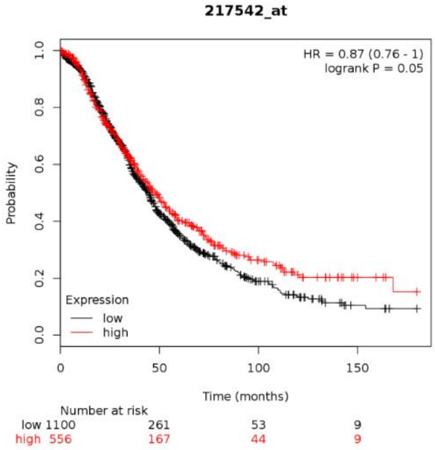
217542_at
HR = 0.87 (0.76 - 1)
logrank P = 0.05
Probability
Expression
low
high
Time (months)
Number at risk
low 1100 261 53 9
high 556 167 44 9

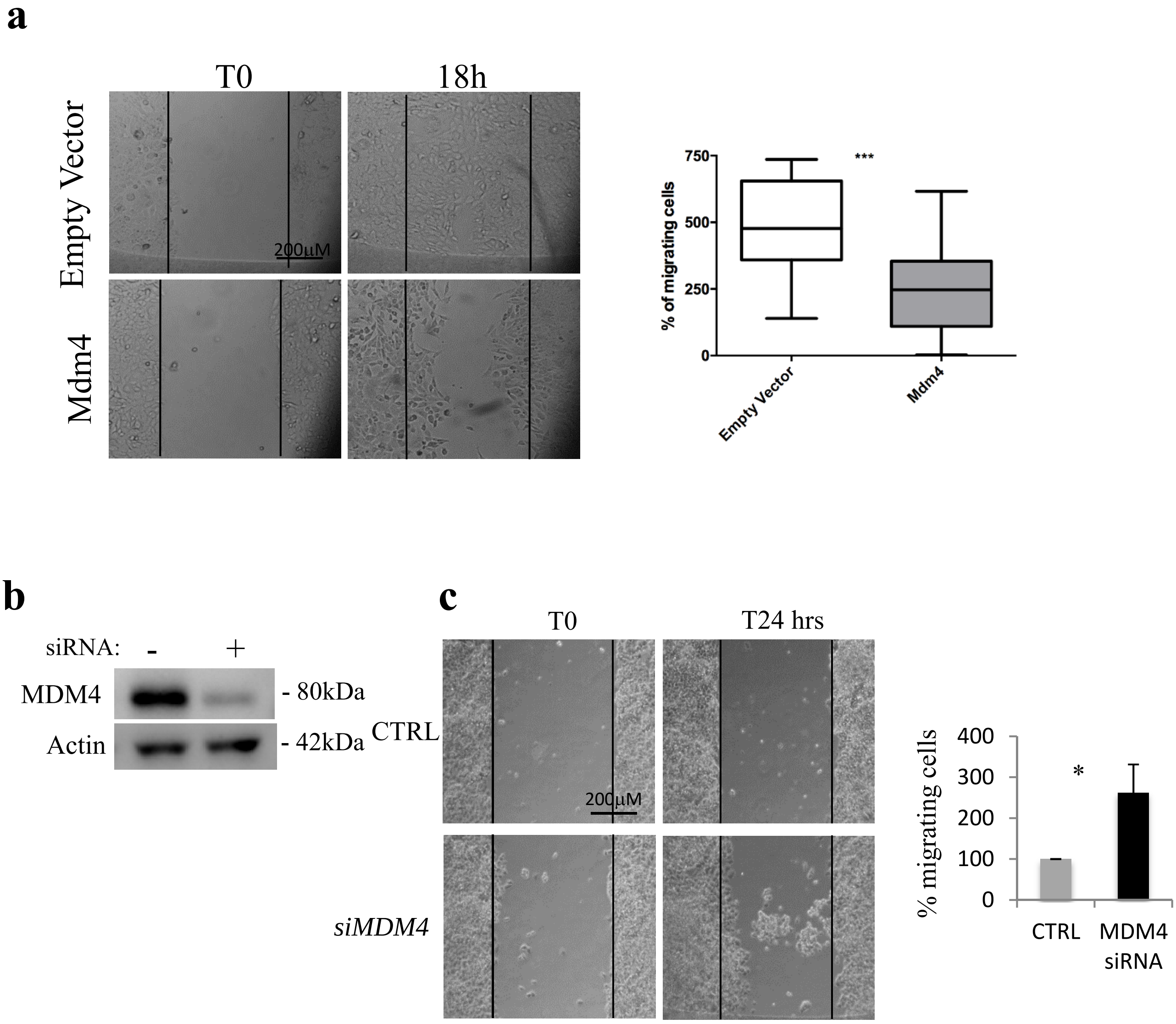
a
T0
18h
Empty Vector
Mdm4
200μM
% of migrating cells
750
500
250
0
***
Empty Vector
Mdm4
b
siRNA:
-
+
MDM4
Actin
- 80kDa
- 42kDa
c
T0
T24 hrs
CTRL
siMDM4
200μM
% migrating cells
400
300
200
100
0
*
CTRL
MDM4
siRNA

a

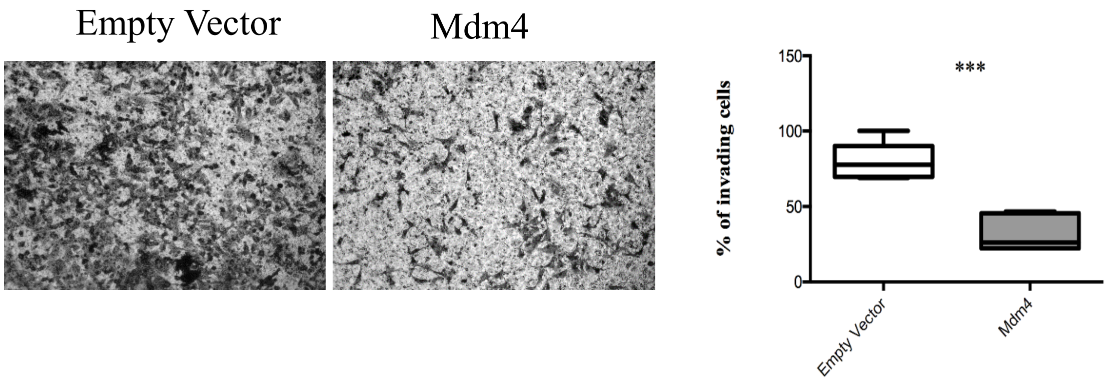
Empty Vector
Mdm4
% of invading cells
150
100
50
0
***
Empty Vector
Mdm4

b

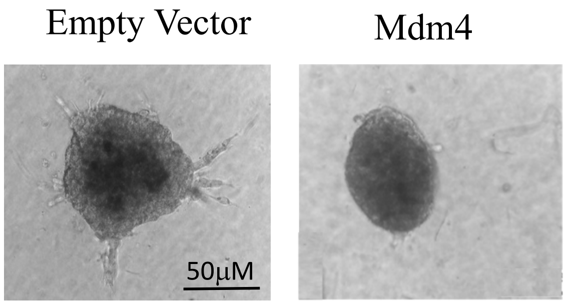
Empty Vector
Mdm4
50μM

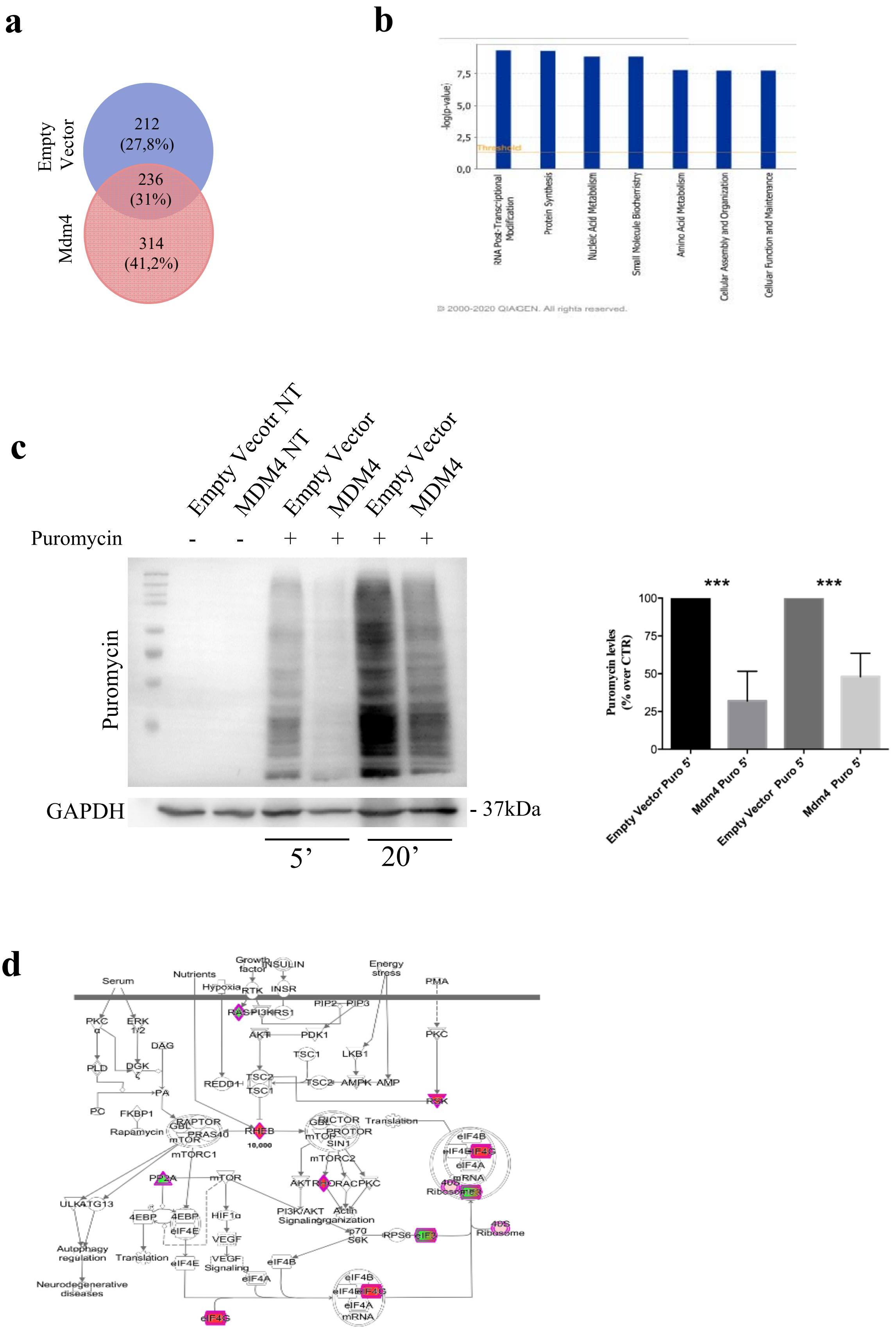
a
Empty Vector
Mdm4
212 (27,8%)
236 (31%)
314 (41,2%)
b
-log(p-value)
7,5
5,0
2,5
0,0
Threshold
RNA Post-Transcriptional Modification
Protein Synthesis
Nucleic Acid Metabolism
Small Molecule Biochemistry
Amino Acid Metabolism
Cellular Assembly and Organization
Cellular Function and Maintenance
© 2000-2020 QIAGEN. All rights reserved.
c
Empty Vecotr NT
MDM4 NT
Empty Vector
MDM4
Empty Vector
MDM4
Puromycin - - + + + +
Puromycin
GAPDH
- 37kDa
5'
20'
Puromycin levles (% over CTR)
100
75
50
25
0
***
***
Empty Vector Puro 5'
Mdm4 Puro 5'
Empty Vector Puro 5'
Mdm4 Puro 5'
d
Serum
Nutrients
Growth factor
INSULIN
Energy stress
PMA
Hypoxia
RTK
INSR
RAS
PI3K
IRS1
PIP2
PIP3
PKC α
ERK 1/2
AKT
PDK1
PKC
DAG
TSC1
LKB1
PLD
DGK ζ
REDD1
TSC2
TSC1
TSC2
AMPK
AMP
PA
PC
FKBP1
RAPTOR
GBL
Rapamycin
PRAS40
mTOR
mTORC1
RHEB
RICTOR
GBL
mTOR
PROTOR
SIN1
mTORC2
Translation
eIF4B
eIF4A
mRNA
40S
PP2A
mTOR
AKT
RHO
RAC
PKC
ULK
ATG13
4EBP
4EBP
eIF4E
HIF1α
VEGF
PI3K/AKT Signaling
Actin organization
p70 S6K
RPS6
40S Ribosome
Autophagy regulation
Translation
eIF4E
VEGF Signaling
eIF4B
eIF4A
Neurodegenerative diseases
eIF4B
eIF4A
mRNA

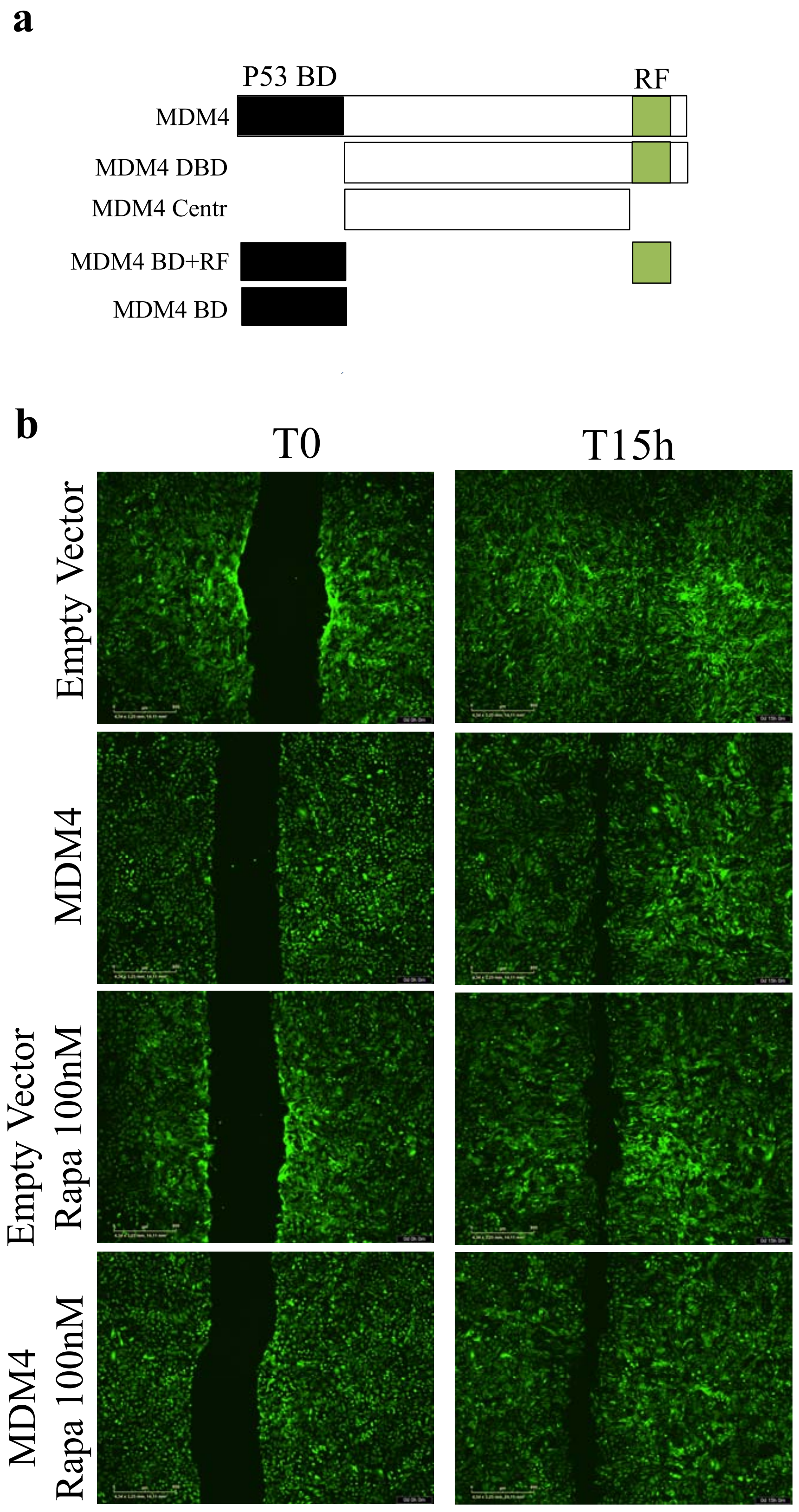
a
P53 BD
RF
MDM4
MDM4 DBD
MDM4 Centr
MDM4 BD+RF
MDM4 BD
b
T0
T15h
Empty Vector
MDM4
Empty Vector
Rapa 100nM
MDM4
Rapa 100nM
